# Comparative gene expression profiling between optic nerve and spinal cord injury in *Xenopus laevis* reveals a core set of genes inherent in successful regeneration of vertebrate central nervous system axons

**DOI:** 10.1186/s12864-020-06954-8

**Published:** 2020-08-05

**Authors:** Jamie L. Belrose, Aparna Prasad, Morgan A. Sammons, Kurt M. Gibbs, Ben G. Szaro

**Affiliations:** 1 Department of Biological Sciences, University at Albany, State University of New York, 1400 Washington Avenue, Albany, NY 12222 USA; 2 Center for Neuroscience Research, University at Albany, State University of New York, 1400 Washington Avenue, Albany, NY 12222 USA; 3 Department of Biology and Chemistry, Morehead State University, Morehead, KY 40351 USA

**Keywords:** *Xenopus laevis*, Spinal cord injury, Optic nerve injury, Axon regeneration, Central nervous system, RNA-seq

## Abstract

**Background:**

The South African claw-toed frog, *Xenopus laevis*, is uniquely suited for studying differences between regenerative and non-regenerative responses to CNS injury within the same organism, because some CNS neurons (e.g., retinal ganglion cells after optic nerve crush (ONC)) regenerate axons throughout life, whereas others (e.g., hindbrain neurons after spinal cord injury (SCI)) lose this capacity as tadpoles metamorphose into frogs. Tissues from these CNS regions (frog ONC eye, tadpole SCI hindbrain, frog SCI hindbrain) were used in a three-way RNA-seq study of axotomized CNS axons to identify potential core gene expression programs for successful CNS axon regeneration.

**Results:**

Despite tissue-specific changes in expression dominating the injury responses of each tissue, injury-induced changes in gene expression were nonetheless shared between the two axon-regenerative CNS regions that were not shared with the non-regenerative region. These included similar temporal patterns of gene expression and over 300 injury-responsive genes. Many of these genes and their associated cellular functions had previously been associated with injury responses of multiple tissues, both neural and non-neural, from different species, thereby demonstrating deep phylogenetically conserved commonalities between successful CNS axon regeneration and tissue regeneration in general. Further analyses implicated the KEGG adipocytokine signaling pathway, which links leptin with metabolic and gene regulatory pathways, and a novel gene regulatory network with genes regulating chromatin accessibility at its core, as important hubs in the larger network of injury response genes involved in successful CNS axon regeneration.

**Conclusions:**

This study identifies deep, phylogenetically conserved commonalities between CNS axon regeneration and other examples of successful tissue regeneration and provides new targets for studying the molecular underpinnings of successful CNS axon regeneration, as well as a guide for distinguishing pro-regenerative injury-induced changes in gene expression from detrimental ones in mammals.

## Background

**Fig. 1:**
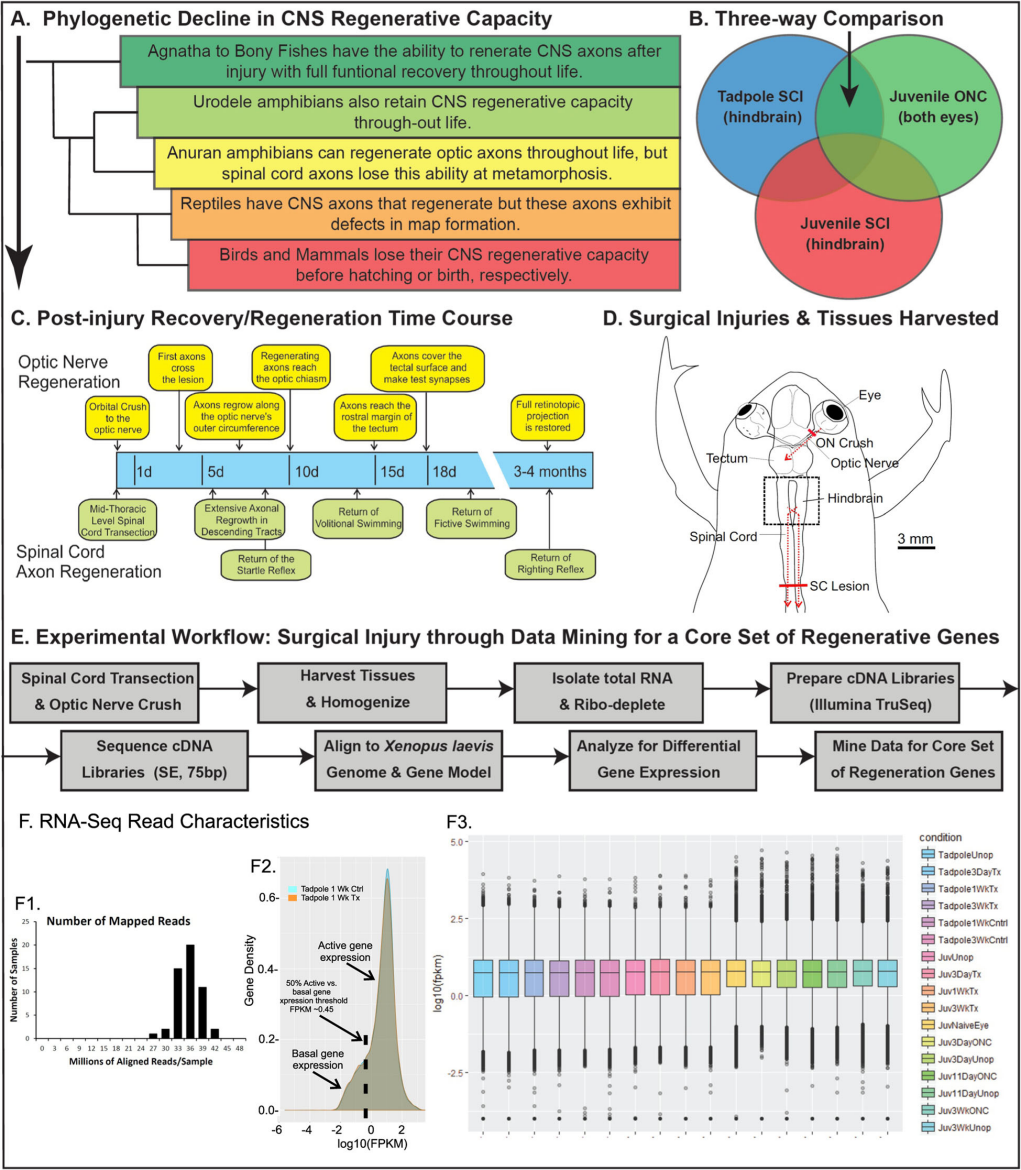
Using *Xenopus laevis* to discover prospective core genetic programs for functional recovery from central nervous system (CNS) injury. **a***Xenopus laevis* occupies a transition point in the phylogenetic decline (green to red) of functional recovery after CNS injury in vertebrates. Like other anurans (yellow), *X. laevis* regenerates optic axons to restore vision throughout life, but only successfully regenerates spinal cord axons as tadpoles. **b** A three-tissue comparison was designed to parse out core sets of genes most closely associated with successful CNS axon regeneration. Injury-induced gene expression profiles (RNA-seq) were compared between two regenerative tissues [stage 53 tadpole hindbrain after spinal cord transection (SCI) and 1–3 month, post-metamorphic, juvenile frog eye after optic nerve crush (ONC)] and a non-regenerative tissue [1–3 month, post-metamorphic, juvenile frog hindbrain after SCI] to find injury-induced genes that were uniquely shared between the regenerative CNS tissues but not with the non-regenerative one. **c** Previous histological, electrophysiological, and behavioral studies in *X. laevis* were consulted to select three time points after optic nerve crush (ONC) and spinal cord transection (SCI) for making suitable comparisons among the tissues - an early trauma phase, when damaged axons first begin to cross the lesion site (3 days), a peak period of maximal regenerative axon outgrowth (7 days for SCI & 11 days for ONC), and a late period, after regenerative axon regrowth is largely completed, but synaptic refinement and behavioral recovery continues (3 weeks) [11, 33, 40, 42, 43, 98, 126, 128, 152, 160]. **d** Scale drawing of the CNS superimposed on the outline of a juvenile frog, to illustrate the injury sites and harvested tissues (ONC and SCI surgeries were done in separate animals; the tadpole is not illustrated, but its hindbrain and spinal cord transection site were similar in location to those of juvenile frog). For tadpole SCI, hindbrains were harvested from operated animals and age-matched unoperated controls (5 pooled hindbrains per biological replicate, with 3 paired injury and control replicates per time point). For juvenile frog SCI, the same unoperated controls were used for all three SCI time points (5 animals pooled into each of 3 biological replicates). For ONC, both eyes of juvenile frogs receiving an orbital nerve crush on the right side were harvested; the right eye provided the ONC samples and the left, contralateral, unoperated eye provided the control (6 pooled eyes for each of three biological replicates per time point). Three biological replicates of surgically naive eyes were also collected (see text). Red bars indicate the anatomical locations of the optic nerve crush and spinal cord lesions. Dotted red arrows indicate the trajectories of the axons injured by the surgeries whose cell bodies are located in the tissues sampled for RNA-seq. **e** Diagram summarizing the workflow of the study (see text for details). **f1**–**3** Summary characteristics of the RNA-seq data. **f1,** a histogram of the number of successfully aligned reads in each of the 51 samples (17 conditions, 3 biological replicates each). **f2,** an example of histograms of expression values [log_10_(FPKM)] per gene, averaged across the biological replicates, normalized for the total number of genes assayed (Gene Density). Data for the 1 week SCI tadpole hindbrain (gray) is superimposed upon that of its age-matched control (blue). The inflection point (dotted vertical line) was used to set a threshold for the fpkm of actively expressed genes. Values below this were categorized as representing no expression. **f3,** Whisker plot summarizing the data dispersion for all 17 conditions (3 biological replicates per condition). The median log_10_(FPKM) is represented as a horizontal line through the box, which in turn delimits the 2nd (lower) and 3rd (upper) quartiles of the data. Whiskers illustrate the 1st and 4th quartiles, with their minimum and maximum values, respectively. Abbreviations: CNS, central nervous system; Cntrl, control; FPKM, fragments per kilobase of exon mapped; Juv., juvenile frog; ONC, optic nerve crush; SCI, spinal cord injury; Tad., tadpole; Tx, transection; Unop, unoperated

The capacity for regeneration is an ancient characteristic of metazoa. Although many invertebrates can reconstitute an entire body from residual tissue fragments [7, 60], vertebrates regenerate only tissues and organs. This capacity becomes more restricted both phylogenetically in the progression from anamniote to amniote and developmentally during the transition from embryo to adult (Fig. 1a). For example, most vertebrates are able to regenerate peripheral nervous system (PNS) axons throughout life and central nervous system (CNS) axons as embryos, but only anamniotes can also regenerate CNS axons as adults. Anuran amphibians, such as the frog *Xenopus laevis*, occupy a transition point in this progression [105]. Like other anamniotes, *Xenopus* regenerates optic axons sufficiently to restore vision throughout life [12, 39, 135], but like amniotes, it loses its capacity to functionally regenerate spinal cord axons developmentally, because of the surge of thyroid hormone that drives metamorphosis in frogs [42] and late fetal development in mammals [8]. Thus, *Xenopus* offers a unique opportunity for exploring within the same organism why some regions of the CNS lose their ability to regenerate axons in development, whereas others retain it. In this study, we exploited these features of *Xenopus* in a novel three-way comparison of RNA-seq data from two regions of the CNS that regenerate axons, one throughout life (juvenile frog eye after optic nerve crush (ONC) [39, 127, 135]) and one that does so only transiently, before and after this transition (tadpole vs. juvenile frog hindbrain after spinal cord injury (SCI) [11, 43]), to identify potential core features that distinguish CNS axon-regenerative responses from failed ones.

With the exception of an mRNA-seq study in lamprey, which demonstrated that the response of brain and spinal cord to SCI indeed differ [48], previous genome-wide expression studies on spinal cord regeneration have focused on either the spinal cord itself, with the lesion at the epicenter (*Xenopus laevis* tadpole and frog [70], turtle [142], zebrafish [50]), or on a regenerating tail (e.g., salamander [87], *Xenopus tropicalis* tadpole [21, 55, 80, 102]). Such regeneration involves not only axon regeneration, but also considerable wound repair and tissue restoration. *Xenopus laevis* hindbrain, which is the principal source of descending axons that project from brain to spinal cord [43, 143], provides an opportunity to compare transcriptional responses to SCI between axon-regenerative and non-regenerative states of CNS neurons and their support cells, separately from the lesion site and its attendant wound repair and tissue restoration. Moreover, hindbrain neurons that regenerate descending axons after SCI (i.e.*,* reticular and raphe nuclei) in the tadpole are the very same cells that fail to do so in the frog [43]. The *Xenopus* eye after ONC provides a similar opportunity to study an injury response, separately from the injury site (Fig. 1d), but after metamorphosis. To date, genome wide studies in the regenerating *Xenopus* visual system have been limited to SDS PAGE [126, 128] and TRAP (RNA-Seq after Translating Ribosome Affinity Purification of mRNAs) studies [151]. Thus, both systems would benefit from baseline RNA-Seq studies of their responses to axotomy.

In performing such an RNA-Seq study, we found that the injury responses of the two regenerative tissues (frog eye after ONC and tadpole hindbrain after SCI) shared features in common with each other not seen in the non-regenerative tissue (frog hindbrain after SCI). Injury-induced genes uniquely shared between the two regenerative tissues included many previously implicated in promoting cell survival and regeneration of neural and non-neural tissues alike, and revealed cellular and physiological processes potentially at the core of a successful regenerative response to CNS injury. We offer these results and the insights gained from their analyses as a resource to stimulate new avenues of investigation into the molecular underpinnings of successful recovery from CNS traumatic injury.

## Results

### Experimental design – animal procedures, sample and data collection, and Bioinformatic analyses

Studies documenting regeneration of CNS axons with return of function after ONC in frogs and the transient nature of functional recovery from SCI injury in tadpoles go back decades [36, 125]. Although initially done in other species, subsequent studies extended these findings to *Xenopus laevis* [11, 39]. Electrophysiological [40, 128], anatomical [33, 128, 152, 160], and ultrastructural [98] studies of *X. laevis* frog optic axon regeneration, as well as behavioral and anatomical studies of tadpole SCI [11, 42, 43], both established the time course of recovery and demonstrated true regeneration of cut axons from surviving neurons, laying the foundation for much subsequent work (e.g., reviewed in [68, 69, 127]). From these studies, three time points can be gleaned for making appropriate comparisons between the two systems (Fig. 1c): 1) an early phase of recovery at three days, when histological studies observed regenerating axons in both systems first penetrate the lesion [11, 160]; 2) the peak phase of regenerative axon outgrowth at seven days for SCI and eleven days for ONC, when the density of regenerating hindbrain axons crossing the lesion nears its peak [11, 42], and when regenerating optic axons fill the optic tract but have not yet arrived at their targets in the tectum [160], respectively; 3) a late recovery phase at three weeks, when regenerating optic axons cover the tectum, but have not yet fully restored a retinotopic map [128, 160], and behavioral recovery is largely but not yet complete in SCI [11, 42]. These same time points were used for the non-regenerative juvenile frog SCI to obtain data on kinetic differences.

In planning the workflow for this study (Fig. 1d), we used the entire tissue encompassing the axotomized neurons, because the cells responding to CNS injuries also included interneurons [2], glia [84], macrophages and other myeloid cells [45, 152], as well as the axotomized neurons themselves. Analyzing complex tissues requires efforts to reduce technical variation as much as possible. By using animals of the same strain and from the same supplier, preparing RNA and cDNA libraries in parallel, and then mixing and sequencing all samples together on the same flow cells, we sought to minimize technical variability, which is otherwise inherent in post hoc comparisons derived from separate studies. To balance budgetary tradeoffs between read depth and the statistical power gained from multiple biological replicates, we sequenced at a nominal depth of 30 million reads per sample, each of which was derived from either five pooled hindbrains or six pooled eyes from co-reared animals, with three such pooled biological replicates for each condition at each time point [25]. This yielded 51 samples representing 17 conditions (Fig. 1e, F3). Because SCI tadpoles were still developing, the unoperated controls for each time point were age-matched and co-reared. For juvenile frog SCI hindbrain, a single control group sufficed for all three time points. For ONC, the left, contralateral unoperated eye served as controls for each time point to further distinguish effects directly associated with axon regeneration from indirect injury responses [126, 128]. This is important because the unoperated contralateral eye also responds to a unilateral nerve crush through indirect effects on retinal circuitry [3, 41, 127]. To assay this indirect response, we included for eye an extra control group of surgically naive, unoperated animals for future studies; this group was included in the PCA but was not used otherwise for differential gene expression analysis (see below).

Fastq files containing the sorted raw sequences were aligned against the *Xenopus laevis* genome (Xenbase v9.1; http://www.xenbase.org/ RRID:SCR_003280) using Bowtie2 (v2.2.9) [66] and TopHat (v2.1.1) [138], and annotated using the Mayball gene model [24, 106, 108]. The alignments yielded 34.4 ± 3.1 (S.D.) million successfully aligned reads per sample (Fig. 1f1), with only 9.6 ± 2.9%, (S.D.) reads initially flagged as potentially duplicate alignments. Such potential duplicates can occur in *Xenopus laevis* due to its ancestrally (~ 30 Mya) duplicated genome [121]. The vast majority of these potential duplicates were resolved by assigning the alignment with the higher score to separate genes on different chromosomes (referred to in *X. laevis* as S and L homeologs). For the small remainder (< 10% of potentially duplicate alignments), reads were randomly distributed between the two homeologs. To confirm the accuracy of this procedure, we visualized a subset directly (Integrative Genomics Viewer (IGV), v2.3.88 [115, 136]). The fastq files and gene counts are both available in Gene Expression Omnibus (GEO; https://www.ncbi.nlm.nih.gov/geo/) under accession number GSE137844. Note here that gene nomenclature in *Xenopus laevis* is based on prospective orthology with human genes [121]. Due to the ancient genome duplication and long evolutionary divergence time between *X. laevis* and human (> 300 Mya), the L and S *X. laevis* homeologs may have more or fewer paralogs than their human orthologs. For the initial gene counts, we enumerated these separately, because duplicate genes can have different expression patterns, potentially leading to different functions [96, 116]. Generally speaking, L homeologs are expressed at higher levels than S homeologs [121], and not surprisingly, they appeared more frequently than S homeologs in our gene lists, since efficiency of gene calls tends to correlate with expression level. Functional comparisons necessarily combined homeologs and paralogs under a single gene term, since most functional studies are done in other species and we cannot a priori evaluate the significance of homeologs and paralogs for these genes. In addition, for this study, we looked only at genes that have been annotated in *X. laevis*, since the principal aim was to evaluate these genes with regard to what is known from other models about function. We left the analysis of fastq files for the biological significance of multiple homeologs and paralogs, as well as of novel genes lacking human orthologs and other gene features such as long non-coding RNAs, to future studies. Nonetheless, for the interested reader, we have noted in Additional_File4_DESR_Data.xlsm the expression behavior of remaining homeologs and paralogs that were not differentially expressed in our current analysis.

**Fig. 2:**
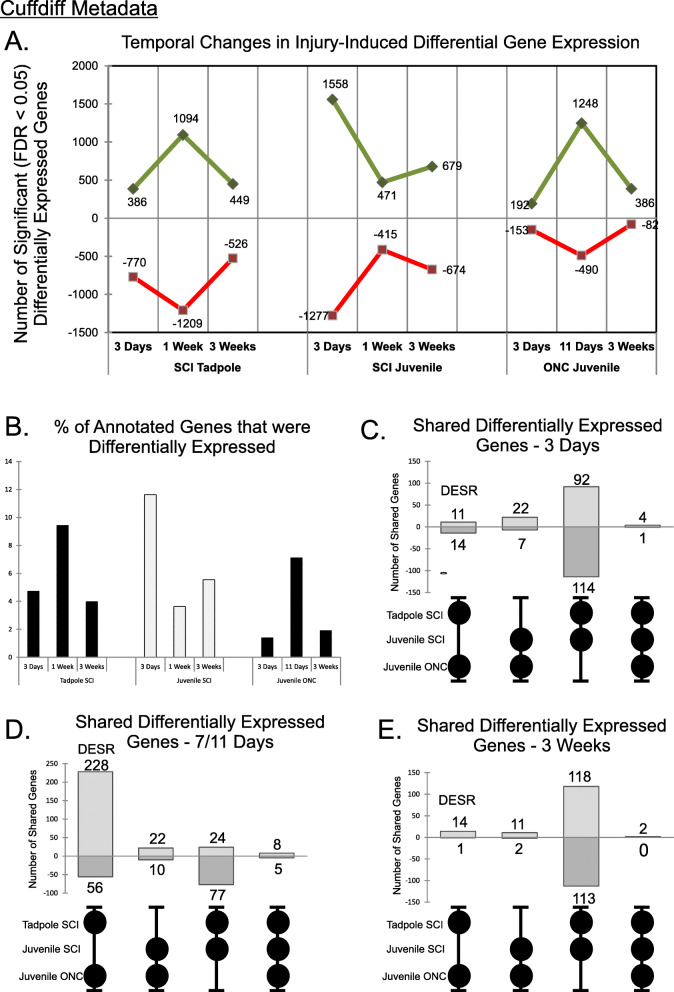
Temporal patterns of gene expression and shared injury-response genes between regenerative vs. non-regenerative tissues. **a** Regenerative tissues [i.e.*,* SCI tadpole hindbrain (SCI Tadpole) and ONC juvenile frog eye (ONC Juvenile)] shared similar temporal patterns of numbers of significant (FDR < 0.05) differentially expressed genes, which differed markedly from that of the non-regenerative tissue [SCI juvenile frog hindbrain (SCI Juvenile)]. Whereas the expression response of the two regenerative tissues peaked during the mid recovery phase (1 week/11 days), that of the non-regenerative tissue peaked at the early, post trauma phase (3 days). Up- and down-regulated genes are shown in green and red, respectively; S & L gene homeologs were tallied separately. **b** Plot illustrating the percentage of annotated genes that were significantly (FDR < 0.05) differentially expressed with injury (100% = 24,382 genes). Additional_File1_Differential_Expression_Analysis_by_Cuffdif.xlsm contains the CuffDiff2 output files from which A and B were derived. **c - e** UpSet plots illustrating the number of genes overlapping between the samples indicated by the circles below each bar at 3 days (**c**), 7/11 days (**d**), and 3 weeks (**e**) after injury. Numbers of shared up- and down-regulated genes are indicated above and below each bar, respectively. The maximum number of overlapping genes between the two successfully regenerative tissues (DESR: Differentially Expressed in Successful Regeneration) occurred during the peak phase of regenerative CNS axon outgrowth. Additional_File4_DESR_Data.xlsm contains the DESR data

To generate gene lists for downstream data analysis of differentially expressed genes (Fig. 1e), we used Cufflinks/CuffDiff2 [113, 114, 137, 139, 140] and the associated utilities of CummeRbund (v2.16.0) [139]. We made this choice primarily because CuffDiff2 uses a paired means test to estimate Type I errors (*p-*values) for each gene pair, independently of other genes. For each pair-wise comparison, these unadjusted *p-*values were then ranked among the other pair-wise comparisons to assess the probability of false discovery (*q*) [14]. Because samples were complex tissues, with multiple cell types potentially responding differently to the lesion, we felt that this procedure of evaluating each gene pair independently was biologically more appropriate for our study than other programs, such as DESeq2, which use a difference of variance test that incorporates the variance of all the genes in a sample into its calculations [4, 79]. In addition, unlike DESeq2, CuffDiff2 normalizes genes for transcript length, making it possible to compare expression levels (FPKM) among different genes. Although we used the CuffDiff2 outputs for downstream analyses (Additional_File1_Differential_Expression_Analysis_by_Cuffdif.xlsm), we provide DESeq2 outputs for the interested reader (Additional_File2_Differential_Expression_Analysis_by_DESeq2.xlsm). Note that the overall trends in the data with respect to temporal patterns of differentially expressed genes and the degree of overlap between different experiments at each time point matched well between the two programs (seen by comparing the CuffDiff2 data of Fig. 2 with that of DESeq2 in Additional_File3_DESeq2_Metadata.pdf), except that DESeq2 identified fewer differentially expressed genes than CuffDiff2, with > 85% of them also called by CuffDiff2.

We used an FDR ≤ 0.05 as the criterion for selecting differentially expressed genes without regard to fold-change, since cell types responding to axotomy represent only a subset of cells in each tissue, and regulatory genes typically exhibit smaller fold-changes in expression than structural genes. We did, however, filter out genes with expressions that fell below the minimal threshold in both the experimental and paired control samples (FPKM = 0.45; Fig. 1f2 [47]). Under these criteria and sampling conditions, differentially expressed genes generally had |log_2_(fold-changes)| > 0.3, and unadjusted *p* values of < 0.02. The percentage of differentially expressed genes in each sample ranged from 1.4% (3 day, juvenile frog ONC eye) to 11.6% (3 day juvenile frog SCI hindbrain) of the total annotated *X. laevis* genome (Fig. 2b; 100% = 24,382 genes).

### Identifying shared features of injury-induced gene expression between axon-regenerative vs. non-regenerative CNS

The principal goal of this study was to identify cellular processes potentially shared between the two regenerative cases that were not shared with the non-regenerative case. The first indication that such processes existed came from the temporal patterns of numbers of differentially expressed genes (Fig. 2a). The numbers of such genes in the axon-regenerative tadpole SCI hindbrain and juvenile ONC eye both reached their maximum at the peak phase of regenerative axon outgrowth (7/11 days), whereas that of the non-regenerative, juvenile frog SCI hindbrain did so at 3 days.

To assess the degree to which these shared responses involved the same genes, we examined at each time point, the overlap among the three tissues (Fig. 2c,d,e). This analysis identified 324 genes that were differentially expressed in the two axon-regenerative CNS regions but not in the non-regenerative one. We termed these genes “Differentially Expressed in Successful Regeneration” (DESR genes: 253 up-regulated; 71 down-regulated, with some appearing at multiple time points). Although the differentially expressed genes in each individual tissue had a predicted FDR of < 0.05, because DESR genes were the result of combining data from two independent experiments, their expected FDR was actually much smaller (< 0.05^2^, which for 324 genes would be ~ 1 gene). Overall, DESR genes comprised approximately 2.7% (324/12,059) of all the injury-induced, differentially expressed genes (Fig. 2c,d,e). This relatively small fraction indicated that the vast majority of differentially expressed genes in each tissue represented tissue-specific responses to axotomy. The DESR expression data, segregated by time point and including information about related paralogs and homeologs, is provided in Additional_File4_DESR_Data.xlsm. Meta analysis conducted on DESeq2 data yielded very similar results (Additional_File3_DESeq2_Metadata.pdf), indicating that these findings were not simply an artifact of the differential expression algorithm.

**Fig. 3:**
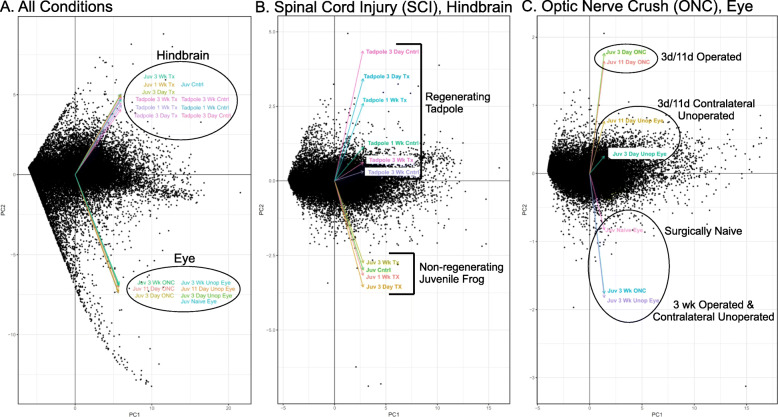
Eigenvector representation of the Principal Component Analyses (PCA) of gene expression profiles. Eigenvectors depict relative degrees of similarity among data sets, as indicated. Black points represent individual genes plotted against the principal axes of similarity (PC1, PC2). **a,** PCA of all 17 experimental conditions and controls. **b,** PCA of SCI hindbrain samples and their unoperated controls. **c,** PCA of ONC operated eye expression profiles, as well as those of their paired, contralateral unoperated control eyes and eyes of uninjured animals (surgically naive). Abbreviations: Cntrl, control hindbrain; Juv, juvenile (1–3 month post-metamorphic) frog; ONC, optic nerve crush; PC1, principal component axis 1; PC2, Principal Component axis 2; SCI, spinal cord injured; Tx, spinal cord transection; Unop, unoperated eye, contralateral to the ONC; Wk, week. Additional_File5_PCA_Scatterplot.pdf shows PCA scatterplots

We next used Principal Component Analysis to obtain an overview of the relationships among gene expression profiles of the various samples (Fig. 3). As implemented in the Tuxedo Protocol (of which CuffDiff2 is a part), PCA generates eigenvectors for each set of pooled biological replicates, with the angles between eigenvectors indicating how well gene expression profiles are correlated between conditions. Diminishing acute angles indicate more highly correlated expression profiles, and orthogonal and increasing divergent angles indicate poorly and negatively correlated profiles, respectively. When all 17 conditions were analyzed (Fig. 3a), the close clustering of the eigenvectors of the eye samples and their large divergence from the closely clustered hindbrain vectors confirmed the conclusion from the overlap analysis that tissue of origin exerted the strongest influence on expression profiles. Developmental stage, injury condition, and time elapsed since injury exerted much less influence. Separate PCA of only the SCI hindbrain expression profiles (Fig. 3b) revealed that within this tissue, regenerative capacity exerted the next strongest influence, with axon-regenerative tadpole eigenvectors clustering separately from those of the non-regenerative juvenile frog. As indicated by the angles between eigenvectors, the injury responses of the axon-regenerative tadpole hindbrains differed more between the operated samples and their age-matched, unoperated controls than was the case for the non-regenerative frog hindbrains, which were more tightly clustered. Moreover, the large divergence between the SCI eigenvectors of their respective peak time points for differential expression (7 days for tadpole and 3 days for frog) indicated that these peak responses involved mostly different genes rather than differences in the timing of differential expression among the same genes. Similarly to tadpole SCI, the eigenvectors of the operated frog ONC eye also diverged extensively from those of their paired, contralateral unoperated eye controls (Fig. 3c). Also similar to tadpole SCI, the close proximity of the 3-week operated and contralateral unoperated eigenvectors with that of eyes from unoperated animals (termed surgically naïve) further indicated that by 3 weeks, the injury response had nearly, but not quite, returned to the pre-injury state. In addition, the divergence of the eigenvectors of all the contralateral unoperated controls diverged from that of surgically naive eye, consistent with the expectation arising from biochemical studies demonstrating that the unoperated eye in a unilateral ONC also responds to the injury [3, 41, 127]. This observation further supports our decision to compare operated eye with its unoperated contralateral control to better emphasize changes directly associated with regenerating an axon, as opposed to more generalized responses associated with the traumatic disruption of visual neural circuitry [3]. Because the bilateral nature of SCI abrogated such a distinction in hindbrain, this comparison in eye acted as an additional filter in the three-way comparison, to resolve those injury-induced differences most directly related to axon regeneration from those related more generally to trauma. Scatterplot PCAs (Additional_File5_PCA_Scatterplot.pdf) generally confirmed the eigenvector PCA analyses, while revealing additional details about the variability of individual biological replicates. For example, the known variability of the ONC injury response at its beginning [3, 41, 151] was readily visible in the PCA scatterplots at 3 days. Similarly in the scatterplots of tadpole SCI hindbrain at 3 weeks, two of three replicates of the operated animals clustered closely with their age-matched unoperated controls, suggesting that animals in these two groups had recovered more fully than had those of the third. Such variability is consistent with the large amount of variability seen from behavioral studies of full recovery times from SCI in tadpoles [11, 42].

### Identifying functional processes in successful CNS axon regeneration

An initial examination of DESR genes supported their biological relevance to CNS axon regeneration, since many had been previously implicated in aspects of neural injury, including (but not exclusive to) axon regeneration [e.g., *socs3* (Suppressor of Cytokine Signaling 3 [32, 107, 134, 151]), *leptin* [9, 13], *fabp7* (fatty acid binding protein 7, a.k.a. brain-lipid binding protein [6, 42, 52, 111]), *mapk8* (JNK1 [51, 97]), *sox11* [95]; *sdcbp* (syntenin [158]), and *prph* (peripherin [41, 84], among others]. The hypothesis that CNS axon regeneration also shares functional processes with other forms of tissue regeneration was supported by some DESR genes having been previously implicated in successful regeneration in other contexts, including axolotl limb [e.g., *tmsb4x* [73, 109], *anxa5* [62], and *crabp2* [85])] and mammalian liver [e.g., *top2A* (DNA topoisomerase 2a [28], *C9* (complement C9 [112, 133]), and *rrm2* (ribonucleoside reductase regulatory subunit M2 [77])].

Thus, we felt justified in using DESR genes to further explore potential cellular processes in successful CNS axon regeneration. For this analysis, we combined two approaches. First, we conducted an automated gene ontology analysis (GO term; Metascape [141]) of the DESR genes at the peak period of regenerative axon outgrowth (1 week/ 11 days for SCI hindbrain and ONC eye, respectively), for significant enrichment (−log_10_(P) > 4) of processes associated with these genes. This analysis primarily yielded GO terms related to cell division and mitotic control, inflammatory responses, innate immunity, wound healing, and stress responses, as well as terms associated with RNA trafficking and turnover, JAK/STAT signaling, development and differentiation, myeloid cell activity, control of gene expression and chromatin remodeling, microfilament/microtubule dynamics, and cellular metabolism. Because relatively few DESR genes were represented at the early and late time points, GO term analysis for these time points was less fruitful, since many genes were omitted. Thus, we complemented the GO term analysis with a manual survey of every DESR gene’s known functions. For this stage of the analysis, we consulted PubMed and Gene Cards for the known functions related to each gene’s orthologs across species, paying special attention to functions related to injury (both traumatic and disease-related) and regeneration (both neural and non-neural), as well as processes identified by the GO term analysis. These functions are listed for each gene individually in Additional_File4_DESR_Data.xlsm. We then grouped DESR genes into categories. The results of this combined summary analysis, with genes arranged according to functional categories and time of differential expression in axon-regenerative CNS regions, are presented in Additional_File6_DESR_Functional_Categories.pdf.

**Fig. 4:**
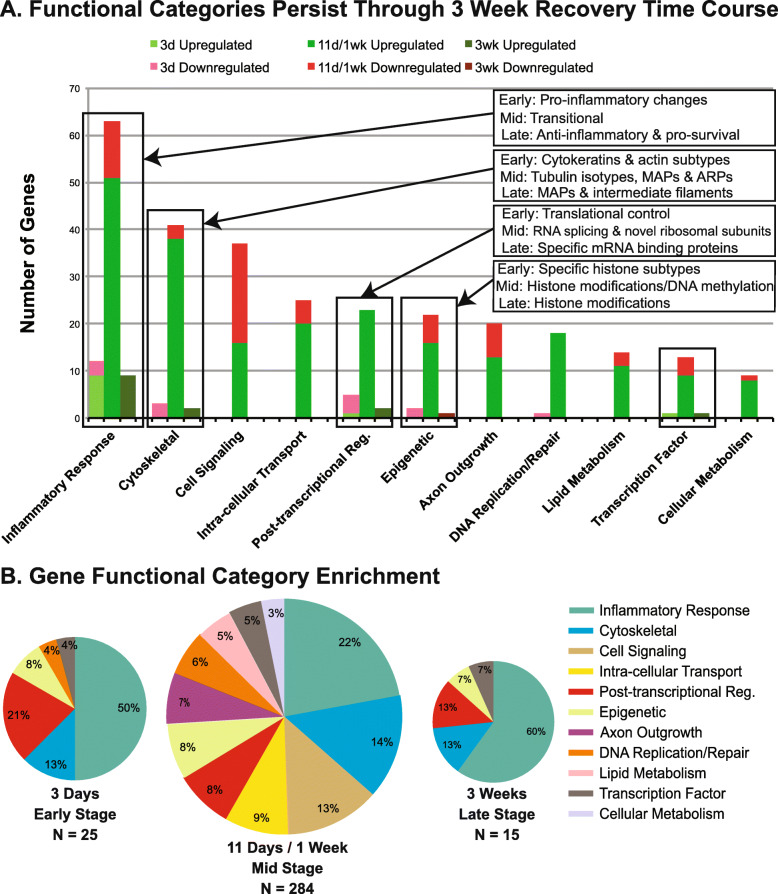
Grouping DESR genes by known functions provided insights into processes underlying successful CNS axon regeneration. **a** Curating DESR genes based on functions documented in the scientific literature (see text for details) parsed them into eleven categories. Vertical boxes outline functional categories present at all three time points. Boxes with arrows list prominent functional sub-categories at the different time points (see text for details). Green shades, upregulated genes; red shades, down-regulated genes (S & L homeologs tallied separately).**b** Pie charts display data from (**a**) according to each category’s relative contribution (%) to the total number of DESR genes and are scaled in size to reflect the total number of DESR genes at each time point (N). Additional_File6_DESR_Functional_Categories.pdf contains a list of the DESR genes, separated by time point and category, along with relevant literature citations supporting the functional categorization. Because functions are mostly based on mammalian studies, and *Xenopus* generally has more than one homeolog for each human gene, they are listed without regard to which homeolog is differentially expressed. Detailed data for individual homeologs are in Additional_File4_DESR_Data.xlsm

In this analysis, DESR genes fell into eleven such categories (Fig. 4; ordered from largest to smallest): Inflammatory Response and Wound Healing, Cytoskeletal, Cell Signaling, Intracellular Transport, Post-transcriptional Regulation of gene expression, Epigenetic control of gene expression, Axon Outgrowth (tropic and trophic effectors and inhibitors), DNA Replication/Repair, Lipid Metabolism, Transcription Factors, and Cellular Metabolism. All eleven categories were represented during the peak regenerative phase, reflecting the wide range of cellular processes that are active then. Five of these eleven were limited to just the peak phase, indicating these processes were relatively inactive at other time points: 1) Cell Signaling, 2) Intracellular Transport, 3) Axon Outgrowth, 4) Lipid Metabolism, and 5) Cellular Metabolism.

DNA Replication/Repair DESR genes were found at only the early and middle time points. At the early time point, one gene in this category was down-regulated in successful but up-regulated in unsuccessful CNS axon regeneration, suggesting it is important for determining regenerative success (*hp1bp3*, a mitotic checkpoint protein involved in maintaining heterochromatin integrity during the G1/S transition [30, 31]). At the middle time point, 5 additional mitotic checkpoint proteins were upregulated, suggesting mitotic checkpoint-related genes are especially important for promoting the peak phase of regenerative outgrowth. Because injured neurons are post-mitotic, these checkpoint genes may have other functions in regeneration than regulating mitosis. In that regard, mitotic checkpoint genes are increasingly understood as important for giving cells time to rearrange their chromosomes in preparation for major changes in gene expression [90]. The remaining twelve up-regulated genes at 7/11 days are typically considered to be more directly involved in DNA replication and repair. They may thus reflect the proliferation of glia and myeloid cells that influence the injury response, since the axotomized neurons themselves are post-mitotic. None of these DNA Replication/Repair genes were found at 3 weeks, indicating that by this time, such activities were no longer active.

The remaining five categories spanned all three time points, providing insights into how these functions evolved throughout recovery. The largest category comprised Inflammatory Response DESR genes. These genes represented functions related to regulating inflammation, innate immunity, wound healing, and cell survival responses, as well as tissue repair and regeneration. Such genes are known principally from mammalian studies, wherein inflammation is crucial for initiating and maintaining pro-regenerative responses to injury [16]. However, because inflammation also damages tissues, it has been difficult from mammalian studies to parse inflammation-related pro-regenerative genes from those detrimental to recovery. Their preferential up- or down-regulation in *Xenopus* axon-regenerative CNS tissues thus provides clues as to which genes are beneficial to regeneration. Moreover, the involvement of many of these genes in wound responses and inflammation is known from studies that either included the injury site or involved non-neural tissues, such as liver or fish tail fin. Thus, their identification in this study also shows their relevance extends more broadly, beyond tissue repair, to CNS axon regeneration. At the early time point, up-regulated Inflammatory Response DESR genes (i.e.*,* recovery promoting) were dominated by ones typically considered pro-inflammatory (7 genes) and genes activated by JAK/STAT signaling through cytokine receptors (3 genes). The two down-regulated genes in this category (i.e., recovery inhibiting) at this early time point were a cytokeratin that is otherwise up-regulated at mammalian wound sites (*krt6a*) and a pro-inflammatory, calcium-binding protein secreted by macrophages (*ocm2*). At the middle time point marking the peak axon-regenerative response, there were two up-regulated pro-inflammatory genes held over from 3 days, and seven new ones, along with many additional up-regulated genes implicated in other inflammatory and wound-healing processes: protein ubiquitination and turnover (11 genes), chaperones (4 genes), cell survival and tissue repair (10 genes), stress response (7 genes), myeloid cell activity (5 genes), and the transition from M1 to M2 macrophages (3 genes). Conversely, down-regulated DESR genes (i.e., regeneration inhibiting) at the middle time point included genes previously associated with exacerbating inflammation, cell death, and scar formation (10 genes), genes associated with oxidative stress (2 genes), myeloid cell activities (2 genes), and with maintaining the blood-brain barrier (1 genes), plus a heat-shock protein (1 gene). At the late time point (3 weeks), all nine Inflammatory Response DESR genes were up-regulated genes previously implicated in protecting cells from detrimental hyper-inflammation and in promoting cellular repair and regeneration, mostly in contexts not previously directly associated with CNS axon regeneration.

The second largest category of DESR genes found at all three time points were those associated with the cytoskeleton. At three days, all four cytoskeletal-related DESR genes were down-regulated: two cytokeratins and two specific actin subtypes. One of the cytokeratins, *krt6a* (previously mentioned in wound healing), has been previously associated with epithelial lesions in mice [153]. Knocking it out makes such wounds more fragile [153], suggesting that in the CNS, this gene helps remodel tissues to promote early stages of axon outgrowth. Moreover, because our sampled tissues did not include the lesion, this function is likely important even at distances removed from the injury site itself. At the peak phase of regenerative axon outgrowth, all the cytoskeletal-related DESR genes were specific tubulin and actin subtypes, along with proteins associated with regulating microtubule- and microfilament-mediated transport and dynamics. All but four were up-regulated (27 total). Collectively, they emphasized the importance of microfilament and microtubule-associated dynamics for axon outgrowth, intracellular transport, cell motility, and cellular proliferation for successful axon-regeneration, while at the same time confirming and extending the identification of specific genes associated with these processes in CNS axon regeneration. For example, *tuba1a* and *tubb2b,* which are neuronal α- and β-tubulin subtypes, had been previously associated with developing and regenerating CNS axons [88, 144], whereas *dynnl2*, a minus end-directed microtubule motor protein involved in the retrograde transport of proteasomes, is novel to axon regeneration. At the late time point, there were only two cytoskeletal-related DESR genes, both of which were up-regulated. They were an intermediate filament gene, *prph* (*peripherin*), which was already known to be up-regulated in both reactive glia and regenerating optic axons in *Xenopus* [41, 84]. *Prph* was also up-regulated in both regenerative and non-regenerative cases at earlier time points, but its preferential up-regulation at 3 weeks in axon-regenerative CNS over non-regenerative CNS suggests its importance in successful regeneration persists into late recovery. The remaining up-regulated cytoskeletal-related gene at 3 weeks, *ebf3*, has dual functions in promoting microtubule bundling and as a transcription co-factor that inhibits gliogenesis [17, 72], suggesting novel functions for promoting recovery during the late phase.

The third category of DESR genes represented at all three time points comprised genes involved in post-transcriptional control of gene expression. Post-transcriptional control of gene expression is already known to be important for successful CNS axon regeneration [51, 76], but the specific genes involved are still being discovered. This study suggests new genes. For example, at the early time point, DESR genes in this category primarily reflected changes in translational control of mRNAs. This is an understudied, potentially important function for promoting successful recovery from CNS injury, because stressed cells utilize cap-independent mRNA translation to ensure that proteins needed for survival are synthesized, while they simultaneously inhibit cap-dependent translation to facilitate cellular reprogramming in preparation for the next phase of the stress response [64]. Our data identified *eif5b*, a translation initiation factor that promotes IRES-dependent mRNA translation [38], as one such gene potentially important for CNS axon regeneration. Another Post-transcriptional Regulation DESR gene at 3 days was a 60S ribosomal protein, *rplp1*, which is essential for brain development due to its effects on cyclin and p63 protein expression [103]. During neural development, specific ribosomal proteins are often needed for selective translation of individual mRNAs [132], and this gene was down-regulated in the two regenerative tissues but up-regulated in non-regenerative frog SCI hindbrain, suggesting a purposeful role in determining regenerative success. The middle time point saw the up-regulation, and no down-regulation, of multiple Post-transcriptional Regulation DESR genes, spanning a range of RNA-related functions, including eight splicing factors, four additional ribosomal subunits, five mRNA translation initiation and elongation factors, and three regulators of mRNA trafficking and turnover. These most likely represent post-translational aspects of the onset of expression of the many genes involved in CNS axon regeneration. Most of these DESR genes were newly implicated here in CNS axon regeneration, although a few had been implicated previously in related contexts. For example, two up-regulated splicing factors, *snrpd3* and *snrpn*, have been associated with neurodegeneration in Spinal Muscular Atrophy [37] and with developmental axon outgrowth [157], respectively. *Prmt1* is the primary methylase targeting hnRNP K [20], which is an RNA-binding protein that regulates nuclear export and translation of cytoskeletal-related mRNAs essential for optic axon regeneration in *Xenopus* [76]. Its selective up-regulation in the two regenerative CNS regions suggests a previously unrecognized role for methylation of hnRNP K, as well as its histone targets, in recovery from CNS trauma. The late recovery phase saw only the up-regulation of two transcripts of RNA-binding proteins that regulate trafficking, translation, and turnover of specific mRNAs: *AldoA* stabilizes mRNAs of neurofilaments [19]), which consolidate axonal growth and expand axon caliber once growing axons contact their targets [118, 120, 146]. Thus, this a*ldoA* increase is consistent with 3 weeks being a phase of consolidation and refinement of synaptic connections [128]. The other up-regulated mRNA, *mex3a,* encodes an RNA-binding protein implicated in regulating neurogenesis and degeneration [10], suggestive of a previously unrecognized role for *mex3a* target-transcripts in CNS axon regeneration.

The fourth most numerous category with representation at all 3 time points (26 genes) comprised genes implicated in epigenetic control of gene expression, which is increasingly recognized as crucial for regeneration of organs and tissues in other contexts (reviewed in [83]). At the 3-day time point, the two DESR genes in this category were down-regulated, specialized histone variants: *hist1h4k*, an H4 gene variant implicated in protecting cells from DNA damage by facilitating DNA double strand break repair [71], suggesting it may play a role here in protecting injured cells; and *hist2h2ab*, an H2 gene variant involved in nucleosome repositioning in preparation for transcription [119]). Nucleosome repositioning is a theme that persisted into the peak phase of regenerative axon outgrowth, with *hist2hab* at this next phase now switching from a down-regulated to an up-regulated DESR gene, and five additional genes known to be involved in nucleosome repositioning (four up-regulated; one down-regulated) appearing. The remaining 19 DESR genes in the Epigenetic category (13 up-regulated, 6 down-regulated) are all known to play various roles in enzymatically modifying histones and DNA through acetylation and methylation. Two of these were down-regulated components of the Polycomb Repressive Complex (PRC), newly implicating it here in successful CNS axon regeneration, although the PRC has already been implicated in tissue regeneration in other contexts [23, 27, 81, 122]. One of these two PRC genes, *jarid2*, persisted as the sole epigenetic-related gene in the late recovery phase, when it continued to be down-regulated.

The final DESR category represented at all three time points comprised genes associated with transcriptional control. At the earliest time point, only one such gene was up-regulated, *ddit3,* which is a C/EBP-related transcription factor needed to activate pro-inflammatory signals [101]. *Ddit3* has been previously reported as up-regulated in response to optic nerve injury in mouse [34] but had not been previously implicated in successful CNS axon regeneration. No transcriptional control DESR genes were down-regulated at this early time point. The peak phase of regenerative axon outgrowth saw eight up-regulated and three down-regulated transcription factors and co-factors. Two of the up-regulated ones were already known from previous studies in mammals to play important roles in SCI – *hes5* and *sox11* [56, 95, 149]; two additional ones (*bcl6* and *mllt11*) were known from cancer studies as modulators of STAT3 [89, 99], which is a transcription factor known to play an important role in regulating inflammation-mediated responses during tissue regeneration. However, to our knowledge, neither of these two regulatory STAT3 co-factors had previously been implicated in successful CNS axon regeneration. Another down-regulated gene in this category at the middle time point was *znf395*, a zinc-finger transcription factor that activates pro-inflammatory cytokines [49], newly implicating it here in limiting aspects of inflammation that are detrimental to CNS axon regeneration. The two sole transcription factor DESR genes at 3 weeks were both up-regulated (*ebf3* and *irf8*). Each has known roles in regulating genes involved in apoptosis and in suppressing a hyper-immune response in macrophages, respectively [53, 72]. These functions are consistent with limiting inflammation and its detrimental effects continuing to be important well into the late stages of recovery.

### The KEGG Adipocytokine signaling pathway comprised multiple DESR genes

**Fig. 5:**
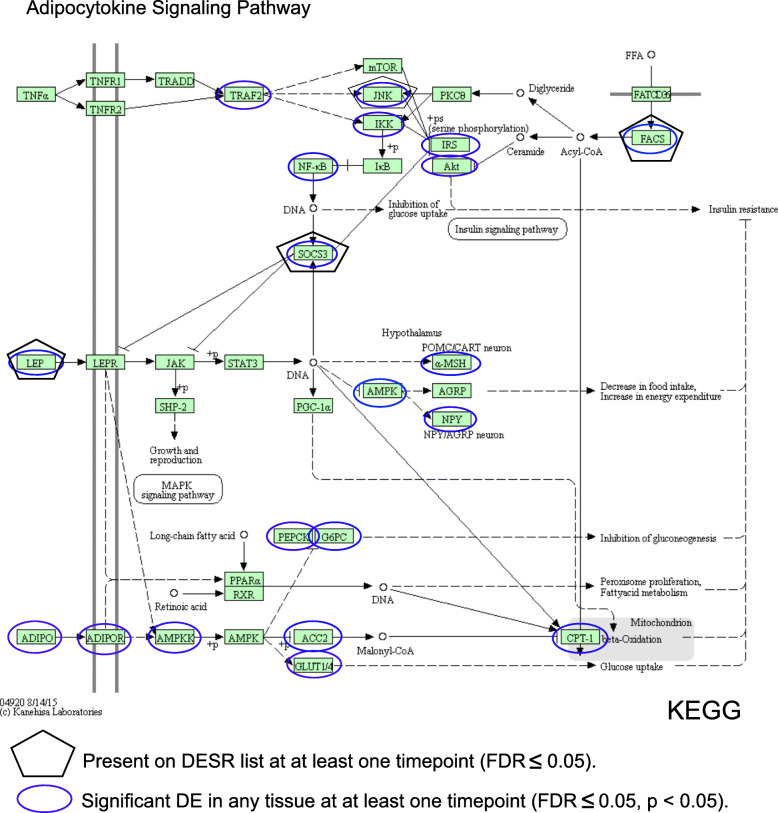
KEGG pathway analyses of DESR genes expressed across multiple time points implicated the Adipocytokine signaling pathway as playing a prominent role in successful CNS axon regeneration. Black pentagons identify DESR’s. Ellipses indicate differential expression (DE; FDR < 0.05) in response to injury (up or down) in at least one tissue, during at least one time point. The Adipocytokine signaling pathway image was obtained from KEGG (Kyoto Encyclopedia of Genes and Genomes). Additional_File7_Adipocytokine_Signaling_Pathway_Gene_Expression_Data.xlsm contains an Excel spreadsheet with all 115 genes (S&L homeologs are separate entries) belonging to this pathway, together with their ΔFPKM values [log_2_(fold change, injury/control)], and their *p* and *q* (FDR-adjusted *p*) values for differential expression, at each time point, for all three tissues

Gene regulatory networks often comprise genes with different cellular functions. Therefore, to identify gene regulatory networks potentially involved in successful CNS axon regeneration, we analyzed KEGG (Kyoto Encyclopedia of Genes and Genomes) pathways for enrichment in DESR genes. In one such analysis we input all the DESR genes found at more than one time point, with the aim of highlighting regulatory networks that were active throughout regeneration. This analysis identified a single network, the KEGG Adipocytokine signaling pathway (map04920; Fig. 5). This network contained four up-regulated DESR genes: *lep* (leptin), *socs3* (suppressor of cytokine signaling 3), *mapk8* (JNK1) and *acsbg2* (Acetyl-CoA synthetase bubblegum family member 2; labeled as FACS in Fig. 5). The up-regulation of these genes suggested they were all pro-regenerative in this context, which was surprising for *socs3*, because in mammalian studies, it is generally considered inhibitory to CNS axon regeneration [123] (but see [107] for a potential resolution to this paradox). In mammalian studies, this pathway interconnects the control of cellular metabolism with inflammatory and stress responses, integrating downstream pathways known from studies across different systems to be involved in various aspects of tissue regeneration. These sub-pathways include JAK/STAT signaling [15, 16, 107, 134], control of axonal transport and synthesis of axonal cytoskeletal proteins via JNK signaling [51, 97], cap-dependent mRNA translation through mTOR [1, 82, 100], and the metabolic regulation of both DNA methylation and histone acetylation/methylation [148, 150]. In addition to these four DESR genes, a further 60% (69/115) of the annotated *X. laevis* genes belonging to this pathway were found to be potentially differentially expressed (either up- or down-regulated) upon injury (*p* ≤ 0.02) during at least one time point, in at least one of the three tissues (Fig. 5). Such complex, temporally variable behavior of these additional network elements suggested that maintaining flexibility of this network throughout regeneration is an important feature for CNS axon regeneration. Collectively, these properties implicate this network as a key hub within the larger network of CNS injury response genes.

### The majority (55%) of DESR genes exhibiting opposing expression between successful and unsuccessful regeneration were functionally inter-connected

**Fig. 6:**
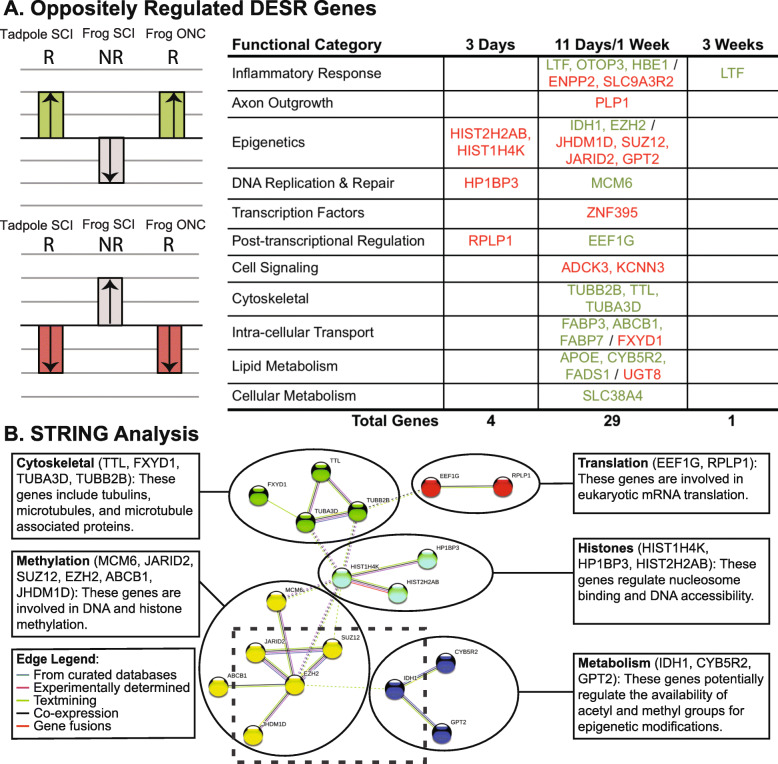
DESR genes exhibiting opposing expression changes in regenerative vs. non-regenerative CNS identified inter-relationships among genes involved in cellular metabolism, post-transcriptional and epigenetic gene regulation, and microtubule dynamics in successful vs. unsuccessful CNS axon regeneration. **a** DESR genes that exhibited opposing expression between the two regenerative tissues (R: tadpole SCI hindbrain & juvenile frog ONC eye) vs. the non-regenerative tissue (NR: juvenile frog SCI hindbrain), sorted by the functional categories of Fig. 4. Green and red indicate genes that were up- and down-regulated significantly, respectively, with injury in the two regenerative tissues (R); these also exhibited significant differential expression with injury in the non-regenerative, juvenile frog SCI hindbrain (NR), but in the opposing direction. Gene symbols correspond with their human orthologs, and S & L homeologs/paralogs are combined into a single listing (see the explanation in Fig. 5). **b** The protein-protein interaction network of the genes in (**a**), as predicted by Search Tool for the Retrieval of Interacting Genes/Proteins (STRING, v10.5). Fifty five per cent (18/33) of oppositely expressed genes between regenerative and non-regenerative tissues were interconnected through interacting functions and molecular interactions associated with the cytoskeleton, DNA methylation, mRNA translation, histones and their epigenetic modifications, and cellular metabolism. The image was generated by STRING (https: //string-db.org; confidence level, 0.300; K-means clustering, k = 5). There were 22 edges with a PPI (protein-protein interaction) enrichment *p*-value = 0.0005. Colors of edges refer to the type of evidence linking the corresponding proteins (see Edge Legend, lower left). Inter-cluster edges are represented as dashed-lines. The dotted square highlights genes directly involved in epigenetic regulation of gene expression at promoters and enhancers. JARID2, SUZ12, and EZH2 are components of Polycomb Repressive Complex 2 (PRC2). EZH2 also plays a role in targeting DNMT3 to DNA. JHDM1D (also known as KDM7) is a principal demethylase for H3K9 and H3K27, prominent sites that interact with PRC2. IDH1 is a metabolic enzyme that stimulates TET, the enzyme principally responsible for converting 5mC to 5hmC & 5C

We next reasoned that genes that were significantly, differentially expressed in opposing directions in the two regenerative cases (R) vs. the non-regenerative case (NR) (i.e., up-regulated in the first two and down-regulated in the last, and vice versa) would be especially instructive in providing insights into changes in gene expression that are purposeful for determining the success or failure CNS axon regeneration. Thirty-three DESR genes exhibited such opposing expression (Fig. 6a; also green and red fonts in Additional_File6_DESR_Functional_Categories.pdf). Genes up-regulated in the regenerative cases and down-regulated in the non-regenerative case, and vice versa, were classified as regeneration-promoting and -inhibiting, respectively. The identities and known functions of thirteen of these genes supported this classification. Nine regeneration-promoting genes were previously known to promote either tissue regeneration and axon outgrowth in general, or cell survival in trauma and degenerative diseases: *ltf* (lactoferrin, an iron-binding, neuroprotective gene [117]), *otop3* (otopetrin 3, a member of a gene family that down-regulates inflammation [147]), *mcm6* (minichromosome maintenance complex component 6, implicated in Müller cell-derived neurogenesis in retinal regeneration [92]), *tubb2b and tuba3d* (neuronal tubulins upregulated in axonogenesis [86, 88]), *ttl* (tubulin tyrosine ligase, required for retrograde transport of pro-regenerative signals after axotomy [124]), *fabp3* (fatty acid binding protein 3, linked to partially successful recovery from spinal cord injury in opossum neonates [94]), *fabp7* (brain lipid binding protein, a marker for radial glial endfeet, which guide growing axons [6]), and *fads1* (fatty acid desaturase 1, linked with suppressing inflammation in liver disease [46]). The functions of the previously known four inhibitory genes were all associated with aggravating inflammation, promoting degeneration, and inhibiting tissue regeneration in various contexts: *enpp2* (autotaxin, a stimulator of inflammation [22, 63]), *slc9a3R2* (a suppressor of STAT3, pro-healing signaling in colon cancer [156]), *plp1* (proteolipid protein 1, a major component of mature oligodendrocytes, which inhibit CNS axon regeneration [65, 155]), and *znf395* (zinc finger protein 395, a transcriptional activator of pro-inflammatory cytokines [49]). The remaining twenty oppositely expressed DESR genes had not previously been associated with processes potentially relevant for CNS injury, inviting further study.

To further explore potential functional inter-relationships among these 33 oppositely expressed DESR genes, we performed STRING analysis for known/predicted protein-protein interactions and functional inter-relationships [130] (Fig. 6b). Eighteen of the 33 genes had such interactions, generating a network of five interconnected clusters (confidence = 0.300): 1) two regulators of mRNA translation; 2) three specialized histones; 3) six other genes associated with epigenetic changes in gene expression, including one such regulated gene that is part of and therefore potentially connects this network to the adipocytokine signaling pathway, *abcb1* [154]; 4) three metabolic enzymes, one of which (*idh1*) regulates the TET enzymes that modulate DNA methylation states [110]; 5) and four microtubule-related genes, which serve to drive cell motility, axonal transport, and axon outgrowth (e.g., [61]). These genes thus form a nascent gene regulatory network potentially linking changes in cellular metabolism with epigenetic reprogramming and post-transcriptionally regulated changes in gene expression, converging upon cytoskeletal structural genes to drive axon outgrowth. At the regulatory heart of this network were genes involved in the epigenetic control of gene expression (dotted-lined box, Fig. 6).

### In situ hybridization of retina to identify cell types expressing key DESR genes

**Fig. 7:**
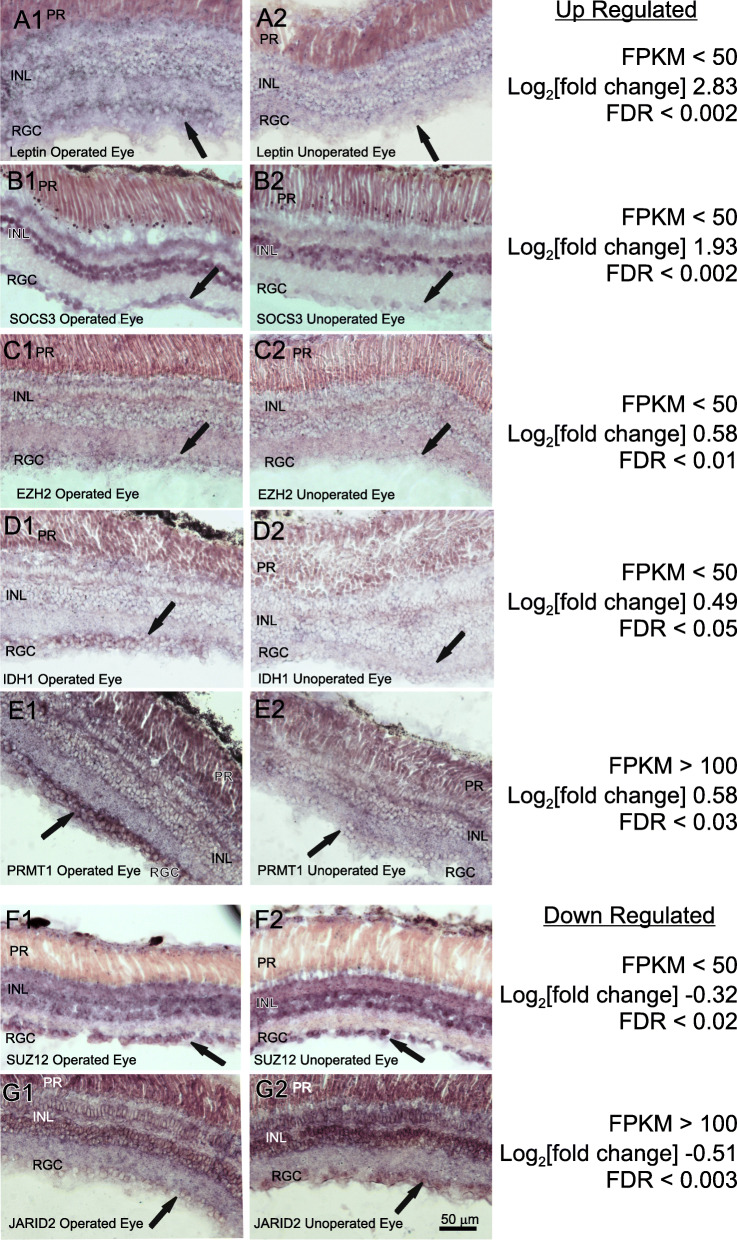
Cellular localization of select DESR genes by in situ hybridization of retina at the peak phase of regenerative axon outgrowth after optic nerve injury. Genes are as indicated in their respective panels and represent a range of fold-change values (0.03 < |log2(fold change)| < 3), and FDRs (0.002–0.05), as well as relatively low (FPKM < 50) and high (FPKM > 100) levels of expression. Examples of up-regulated (**a** – **e**) and down-regulated (**f**, **g**) genes are included. Column 1 (left), operated eye; column 2 (right), contralateral unoperated eye from the same animal and processed on the same slide as that of its adjacent column. Arrows indicate cells of the retinal ganglion cell layer, which comprises the neurons that regenerate an axon. Abbreviations: RGC, retinal ganglion cells; INL, inner nuclear layer; PR, photoreceptors. Scale bar in G2 applies to all panels

To begin to identify cell types responding to axotomy through expression changes in key DESR genes, we performed in situ hybridization on retina at the peak phase of regenerative axon outgrowth for select genes, focusing primarily, but not exclusively, on the two networks just discussed (Fig. 7). We chose retina over hindbrain for these studies because the anatomical organization of retina greatly simplifies identifying cell types. For example, because frog retina receives no external afferents, ONC damages only the optic axons, which originate from retinal ganglion cells. These cells constitute the vast majority of cells in the ganglion cell layer, making them anatomically distinct from other local circuitry neurons, such as bipolar cells, and Müller cell astroglia. Both cell types can be indirectly affected by optic nerve injury and their cell bodies occupy other retinal layers [2, 84]. The situation is more complex in hindbrain, which harbors not only the axotomized neuronal cell bodies but also ascending axons damaged by the SCI. In addition, the axotomized hindbrain neurons are anatomically intermixed with other local neurons, making it essential to distinguish them through applying retrograde tracers [44]. Finally, because the injured and control hindbrain are necessarily in different animals, it is harder to ensure that hybridization conditions are precisely matched than it is for retina, where both the operated and unoperated eye occupy the same section.

For key up-regulated DESR genes, we chose *leptin* and *socs3* from the Adipocytokine Signaling pathway, *prmt1*, which methylates both hnRNP K and histones, and *idh1* and *ezh2*, which are two epigenetic-related genes from the network of Fig. 6b. For down-regulated genes, we chose *jarid2* and *suz12,* which belong to the Polycomb Repressive Complex 2 and are also part of the same network as *idh1* and *ezh2* (Fig. 6b). These genes were also chosen because they represented a wide range of expression levels, from abundantly expressed (FPKM > 100) to moderately and poorly expressed genes (FPKM < 50), and because they spanned the range of FDRs from <.002 to 0.05. Relative differences in expression from experimental to control (|log_2_(fold-change)|) also spanned the full spectrum – from 0.3 (the smallest significant change) to 2.8 (one of the largest). (It should be noted here that because probes for in situ hybridization do not distinguish between homeologs, the RNA-seq data in Fig. 7 represent values combined for the two homeologs). In all cases, the overall intensity of labeling and the relative difference between operated and unoperated eyes reflected the magnitudes seen in the RNA-seq data. Increases in the two up-regulated adipocytokine signaling pathway genes, and in *idh1* and *ezh2* were confined to the retinal ganglion cell layer. The changes in expression of the remaining up-regulated gene, *prmt1*, as well as the two down-regulated components of the Polycomb Repressive Complex 2 (*jarid2, suz12*), included but also extended beyond the retinal ganglion cell layer, indicating that these changes in expression occurred more globally throughout the retina.

## Discussion

We used a novel three-way comparison between a region of the CNS that successfully regenerates axons throughout life (retina) against another that successfully regenerates axons only transiently in development (hindbrain), before and after the transition, to serve as a biological data filter for parsing core features of a successful CNS axon-regenerative response from the thousands of injury-induced changes in gene expression that otherwise represented region-specific responses. The first indication that the two regenerative cases uniquely shared core features was the temporal pattern of the number of injury-induced genes. Whereas this number reached a maximum in the two regenerative cases during the peak phase of axonal regrowth, it did so in the non-regenerative case at the earliest time point. PCA corroborated the conclusion that gene expression profiles were largely tissue-specific, but closer inspection identified shared features between the two regenerative tissues. For example, PCA eigenvectors (Fig. 3) and scatter plots (Additional_File5_PCA_Scatterplot.pdf) differed more between injury and controls for both regenerative cases than was the case for the non-regenerative case. The lack of overlap between regenerative tadpole and non-regenerative frog SCI hindbrain, even for different time points, further indicated that the genes induced by SCI were quite different between these two tissues throughout regeneration. Individual DESR genes (324) far out numbered injury-induced genes shared by all three tissues (19 genes) at all time points, as well as the number shared between regenerative and non-regenerative SCI hindbrain at the peak phase of axonal regrowth (284 DESR genes vs. 101 shared regenerative/non-regenerative SCI hindbrain genes). Collectively, these data supported the conclusion that beginning relatively soon after injury, having the capacity to mount a successful axon-regenerative response stimulates a markedly different injury response from that of a non-regenerative tissue, which in the latter, is more focused on attempting rapid repair for survival than on axon regeneration.

Further insights into the nature of the cellular processes uniquely shared between the two regenerative cases came from the identities and subsequent analyses of the DESR genes. In support of these genes being relevant to CNS axon regeneration, DESR genes included multiple genes previously implicated in CNS injury, although mostly in other contexts, such as studies that included the injury site, peripheral axon regeneration, and resistance to neurodegenerative disease. This observation emphasized the universality of those genes in other types of CNS injury, as well as the phylogenetic conservation of the processes they represented, thereby building confidence in the data set. The data also included a large number of DESR genes shared across a broad spectrum of non-neural regenerating tissues, including zebrafish tail fin, axolotl limbs, and mammalian liver, arguing further that their importance extends beyond CNS axon regeneration to vertebrate tissue regeneration in general, and providing clues into aspects of a successful regenerative response that are universally shared among all vertebrate regenerating tissues. The many additional, novel genes implicated here for the first time in CNS axon regeneration provide new targets for investigation.

Surveying the literature for what is known about each DESR gene’s function sorted them into eleven broad functional categories. Temporal differences in the composition of these categories provided further insights into how core processes associated with successful CNS axon regeneration shifted as axon regeneration progressed. For example, the onset of axon regeneration (3 days) was dominated by up-regulated pro-inflammatory genes, several down-regulated cytoskeletal genes potentially associated with scarring, and by genes indicative of cellular reprogramming events. The latter included an up-regulated gene associated with promoting non-cap dependent mRNA translation (*eif5b*) and several down-regulated ribosomal subunits, two down-regulated histone subtypes associated with transcriptional activation, a down-regulated mitotic check point protein (*hp1bp3*), and an up-regulated C/EBP-related transcription factor (*ddit3*) that is regulated by stress factors associated with bacterial infections and with mammalian optic nerve injury but which had not previously been implicated in successful CNS axon regeneration [34, 101]. At the peak phase of regenerative axon outgrowth, all eleven functional categories were represented, indicating high levels of activity associated with these functional categories at the most active time point. While the presence of DESR genes previously implicated in regenerative and developmental axon outgrowth helped to provide confidence in the relevancy of the data to CNS axon regeneration, our finding additional genes associated more generally with cellular processes previously associated with wound healing and tissue repair in other contexts provides new gene targets for studying how the cellular processes with which they are connected function specifically in CNS axon regeneration. These processes included modulators of Wnt-signaling [48], macrophage invasion and the complement cascade [35, 74, 104], and modulators of JAK/STAT signaling [54, 134]). Still more genes offered important clues about which aspects of an inflammatory response are pro-regenerative (i.e., up-regulated DESR genes) as opposed to detrimental (i.e., down-regulated DESR genes) to CNS axon regeneration, a distinction that has been difficult to make from mammalian studies [16]. Other DESR genes implicated cellular processes that are new to CNS recovery (e.g., regulators of mitotic checkpoints). The late time point, when axonal regrowth was largely finished but synaptic connections were still being refined, was dominated by up-regulated genes known to promote cellular repair, suppress hyper-inflammation, and stabilize the cytoskeleton. These functions are understandable during a time when the initial effects of trauma have been overcome, terminal axonal arbors are being refined and consolidated, and physiological gene functions are returning; the identification of new, specific genes involved in these processes provide new entry points for studies aimed at understanding and promoting these beneficial processes.

To begin to elucidate gene regulatory networks potentially occupying important hubs in the network of gene responses promoting successful CNS axon regeneration, we engaged in two exercises. In the first, we surveyed KEGG pathways enriched with DESR genes, especially genes that were differentially expressed at multiple time points. This analysis identified the KEGG Adipocytokine signaling pathway as enriched both for DESR genes and for injury-induced differentially expressed genes in general, independent of tissue regenerative capacity. The four DESR genes in this pathway were uniformly up-regulated, suggesting that their activation within this network promotes regeneration. However, multiple other differentially expressed genes belonging to this network exhibited both up- and down-regulation at various times in each of the tissues, further suggesting that the dynamic behavior of this network influences regenerative success. The importance of individual components of this pathway for wound repair and regeneration had already been noted across a range of systems, both neural and non-neural. For example, leptin in mammals has been implicated in the regenerative success of both liver and muscle [67, 91, 145], and in zebrafish, it has been implicated in both restoration of a damaged retina [159] and in recovery from heat-induced stress [78]. In *Xenopus*, up-regulation of leptin has also been noted in the regenerative response of the spinal cord itself to SCI [134], and during development, it is involved in activating canonical Wnt-signaling in neurogenic regions of the brain [13]. Our linking leptin to axon-regenerative regions of the CNS that are otherwise uninjured, and localizing the increased expression to the axotomized retinal ganglion cells, which do not themselves undergo active proliferation after axotomy, suggests that these cells use leptin to signal nearby support cells, such as invading macrophages [45, 152] and reactive Müller glia [84]. Canonically, leptin and the adipocytokine signaling pathway play a crucial role in thermoregulation and dietary intake in mammals [18, 26]. Thus, our data from *Xenopus* adds to a growing body of evidence supporting a phylogenetically ancient linkage in vertebrates between the regulation of cellular metabolism, the stress response, and the success or failure of organ and tissue regeneration both within and outside the CNS, where the axotomized neurons themselves may be actively involved in the signaling.

Next, we examined the subset of DESR genes that exhibited opposing expression patterns between regenerative and non-regenerative tissues, reasoning that these genes could provide insights into purposeful differences between the two. For example, genes that were up-regulated in the regenerative cases but down-regulated in the non-regenerative case would likely be pro-regenerative and vice versa. Of the 33 oppositely expressed DESR genes, nine pro-regenerative (i.e., upregulated in axon-regenerative CNS and down-regulated in non-regenerative CNS) and four regeneration inhibitory (i.e., down-regulated in axon-regenerative CNS and up-regulated in non-regenerative CNS) genes had previously been implicated in either promoting or inhibiting regeneration and cell survival, respectively, in one or more contexts, thereby helping to validate the list while expanding its relevance to include CNS axon regeneration. Whereas KEGG analysis fits gene sets into known biological pathways, STRING analysis can be used to discover new gene networks from known interactions, both physical and functional. STRING analysis of these 33 oppositely expressed DESR genes placed the majority (18) into five interconnected clusters, with three clusters comprising specialized histones and genes associated with epigenetic control of gene expression at the center. These five clusters could thus conceivably comprise the core of a nascent gene regulatory network for successful CNS axon regeneration. In situ hybridization of a subset of genes in this network found that all the pro-regenerative genes were specifically up-regulated in the axotomized retinal ganglion cells, whereas the two regeneration-inhibitory components of the network, which are part of the Polycomb Repressive Complex 2, were down-regulated in multiple retinal layers. Surprisingly, another member of Polycomb Repressive Complex 2, *ezh2* was up-regulated in axotomized retinal ganglion cells. Since *ezh2* functions extend beyond the complex to include methylation of DNA as well as histone sites [75], its pro-regenerative activity is therefore likely to be separate from its functions within the Polycomb Repressive Complex. Examination of the known functions of the genes in the central three clusters, together with other epigenetic-related DESR genes (25 total), strongly implicate increasing the accessibility of DNA for transcription as being of central importance to determining the success or failure of CNS axon regeneration.

## Conclusions

A novel three-way comparison identified candidate genes belonging to core processes shared by two CNS regions that successfully regenerate axons, that distinguished them from one that does not (i.e., juvenile frog ONC eye vs. tadpole SCI hindbrain vs. juvenile frog SCI hindbrain, respectively). Unlike earlier studies of SCI that included the lesion in the analyzed tissues, by analyzing the tissues of origin of the regenerating axons, this study emphasized genes relevant for CNS axon regeneration over wound repair and tissue restoration. Even so, many of the genes identified in our study were previously implicated in wound healing, tissue repair, and cell survival, demonstrating that these processes are shared with successful CNS axon regeneration. Because these studies were mostly conducted in other tissues and species, our study both demonstrated their phylogenetic conservation and identified many new individual genes associated with these processes as worthy targets of study in CNS axon regeneration. Finally, because many of these genes were implicated in mammals, where recovery is often incomplete, their identification in this study provides clues as to which are beneficial to recovery and which ones are detrimental. Network analyses of DESR genes that were up-regulated at multiple time points and of DESR genes that were oppositely expressed in axon-regenerative vs. non-regenerative CNS regions, respectively, identified the Adipocytokine signaling pathway and a novel gene regulatory network with epigenetic control of gene expression at its core, as important hubs in the larger network of injury-response genes associated with successful CNS axon regeneration. In situ hybridization in ONC retina of representative genes from these networks showed that the injury response of some elements of these networks were restricted to the axotomized neurons, whereas others extended to neighboring cells, demonstrating that a successful CNS axon-regenerative response to axotomy indirectly invokes responses in neighboring cells that could influence its success. This study thus provides both a resource and a starting point for future investigations into the molecular underpinnings of successful CNS axon regeneration.

## Methods

### Animal and surgical procedures

All animal procedures were approved by the Institutional Animal Care and Use Committees (IACUC) of the University at Albany (optic nerve crush) and Morehead State University (spinal cord transection), in accordance with the National Institutes of Health Guide for the Care and Use of Laboratory Animals. For the sake of consistency, *Xenopus laevis* tadpoles and juvenile frogs were all from an albino strain obtained from the same supplier (*Xenopus* Express, Brooksville, FL). Prior to surgery, animals were acclimated to the lab for at least a week, under a 12-h light:12-h dark photoperiod, in tanks of conditioned water at 22 °C, where they were fed every other day (*Xenopus* Express SFF and TP, for frogs and tadpoles, respectively). Optic nerve crush (ONC) surgeries were performed on fully anesthetized (0.1% MS222, Sigma-Aldrich), 1–3 month, post-metamorphic albino frogs (unsexed juveniles), as described [160]. Briefly, the surgically exposed optic nerve on the right side of the animal was crushed at the orbit, approximately 0.5–1 mm from the eye, using #5 Dumont forceps (Fig. 1d), 2–4 times, until the nerve turned visibly clear within the epineural sheath. The contralateral left eye served as the unoperated control. Spinal cord transections (SCI) were performed on fully anesthetized NF [93] stage 53 tadpoles (0.02–0.04% MS222) and 1–3 month post-metamorphic juvenile frogs (Fig. 1d) of the same age as those used for ONC [42, 44]. The surgically exposed spinal cord was completely transected at the mid-thoracic level, and the success of the surgery was confirmed at the time of surgery by passing a Minutien pin (Fine Science Tools) through the injury site while observing the spinal cord. To further confirm that spinal cord transections were complete, animals were tested the next day for residual movement. After surgery, animals were maintained in tanks filled with aerated, sterile-filtered, conditioned rearing water containing 4 mg/l gentamicin sulfate (Sigma-Aldrich) to guard against infection.

### RNA isolation

For each biological replicate, either five hindbrains (SCI) or six eyes (ONC) were rapidly dissected from fully anesthetized (0.1% MS22) animals [3, 41, 42]. To maximize RNA integrity, hindbrains were submerged in RNA*later* (ThermoFisher Scientific) at room temperature, and eyes were homogenized immediately. Tissues were homogenized (Polytron® PT 3000) at 23 °C in 500 μl of Lysis Buffer Q (Norgen Biotek Corp., Ontario, Canada), supplemented with 5 μl β-mercaptoethanol. Homogenates were then cleared by centrifugation (23 °C, 15,000 rpm, 15 min), and total RNA was isolated [RNA/DNA/Protein Purification Plus Kit, Norgen Biotek Corp (cat# 47700); RNase-free DNase I kit, Norgen Biotek (cat# 25710)]. A 2 μl aliquot of this RNA solution was quantified (Nanodrop® ND-1000 Spectrophotometer), and RNA quality was preliminarily assessed by agarose gel electrophoresis (E-Gel® EX Agarose, E-Gel® iBase™ Power System, ThermoFisher Scientific).

### Library preparation and sequencing

Purified total RNA from all 51 samples was simultaneously sent for library preparation and sequencing (Center for Genomics and Molecular Medicine, University of Louisville). There, RNA was again quantified (Qubit™ Fluorometer) and assessed for quality [2100 Bioanalyzer, Agilent Technologies; RINʼs (RNA Integrity Number): 9.0 ± 0.7 (mean ± SD; *n* = 51), range 7.6–10.0]. Ribosomal RNA-depleted RNA (0.5 μg) was subsequently used to make barcoded cDNA libraries [TruSeq Stranded Total RNA LT Sample Prep Kit - Set A and B (Illumina, cat# RS-122-2302), with Ribo-Zero® Gold (Illumina)], which were pooled for sequencing [NextSeq 500; NextSeq 500/550 75 cycle High Output Kit v. 2 (Illumina)] in two runs to nominally yield 30 × 10^6^, 75 base-pair (bp), single-end reads.

### Read alignment and differential expression analysis

After assessing the raw fastq files for quality (FastQC v0.11.5 [5]), reads were aligned against the *Xenopus laevis* genome (Xenbase v9.1; http://www.xenbase.org/ RRID:SCR_003280) using Bowtie2 (v2.2.9) [66] and TopHat (v2.1.1) [138], and then annotated using the Mayball gene model [24, 106, 108]. The alignments yielded 34.4 ± 3.1 (S.D.) million successfully aligned reads per sample (Fig. 1f1), with only 9.6 ± 2.9%, (S.D.) reads initially flagged as potentially duplicate alignments, which can occur in *Xenopus laevis* due to its ancestrally (~ 30 Mya) duplicated genome [121]. These potential duplicate alignments were resolved for the vast majority by using the alignment with the higher score to assign them to separate genes on different chromosomes (referred to in *X. laevis* as S and L homeologs). For the small remainder (< 10% of potentially duplicate alignments), reads were randomly distributed between the two homeologs. To confirm the accuracy of the alignments, a subset was visualized directly (Integrative Genomics Viewer (IGV) (v2.3.88) [115, 136]).

Differential expression and associated analyses [dispersion plots, gene density histograms, and eigenvector principal component analysis(PCA)] were carried out using Cufflinks/Cuffdiff2 (v.2.2.1; per-condition dispersion with a minimum count of 10) [113, 114, 137, 139, 140] and associated utilities of CummeRbund (v2.16.0) [139]. The gene density histograms were used to set the threshold (FPKM = 0.45) for active gene expression (Fig. 1f2), and the dispersion plots were used to affirm that samples being compared statistically had similar dispersions (Fig. 1f3). In CuffDiff2, reads are normalized for transcript length, which enables comparing expression levels among different genes, and a difference of means test is performed across the biological replicates on the log_2_(fold-changes) [i.e., log_2_(FPKM_injury_/FPKM_control_)] for each gene separately to obtain a *p* value. These *p* values are then ranked to calculate an FDR (*q*) [14]. Differences in individual tissues were considered significant for FDR < 0.05, without regard to the magnitude of the change.

The same read alignments and gene counts, after they were rlog-transformed, were also analyzed by DESeq2 [4, 79], and scatter-plot PCA representations were generated using the plotPCA function of DESeq2. For the latter, multiple PCA plots were generated over the range of 500 to 20,000 of the most highly-expressed genes, for which no qualitative differences were observed. DESeq2, unlike CuffDiff2, does not normalize genes for transcript length and uses a variance test, which incorporates the variance of all the genes in a sample, instead of a means test that treats the variance of each gene separately. Because the tissues being analyzed comprised a complex mix of cell types, each of which may respond differently to the lesion, we used the CuffDiff2 data sets for subsequent downstream analyses.

### Gene ontologies of differentially expressed genes

For each time point, overlapping differentially expressed genes across the three conditions were identified using Awk scripts. To identify DESR genes, the script first identified differentially expressed genes in both regenerative tissues, then removed any that were also present in the non-regenerative one. The final DESR gene list was then analyzed for gene ontology (GO term) enrichment (Metascape (v3.0) [141]). Then, a literature search (PubMed; GeneCards) was conducted for each DESR gene for additional information related to its function that was relevant to processes potentially involved in CNS regeneration [i.e., wound healing, inflammation, innate immunity, tissue regeneration or degeneration, and axon outgrowth], and as suggested by the GO term analysis, to yield the final classification of genes into 11 functional categories.

### KEGG pathway and STRING analysis

DESR genes appearing at multiple time points were entered into the KEGG pathway analysis tool (Kyoto Encyclopedia of Genes and Genomes [57–59]; https://www.genome.jp/kegg/). Oppositely regulated DESR genes (i.e.*,* up-regulated in both regenerative tissues but down-regulated in the non-regenerative tissue, and vice versa), were analyzed by the Search Tool for Retrieval of Interacting Genes/Proteins to identify potential functional interactions (STRING, v10.5 [129, 131]; https://string-db.org; confidence level, 0.300; K-means clustering = 5). The Human Protein Complex Map (hu.MAP; http://proteincomplexes.org/) was used to identify proteins that physically interact within macromolecular complexes [29].

### In situ hybridization

cDNA templates for in vitro transcription of antisense cRNA probes for in situ hybridization were prepared by reverse transcriptase (Super Script IV VILO, Invitrogen) polymerase chain reaction (Phusion High Fidelity PCR, New England Biolabs) from total RNA isolated from juvenile frog brain, with the following primers: Leptin (LEP.L; NCBI Accession number XM_018252815; length 278 nt) forward – GAT CCA AGG ACG AGC TAT AAA AAC T, reverse – GTA ACA GAC TGC GGA GGT TCT; SOCS3.S (NCBI Accession number NM_001087305; length 611) forward– ATG GTA ACG CAG AGC AAG TTC CCG, reverse– CGT TTT CTT TGT CTA CAC TGG GGA; EZH2.L (NCBI Accession number XM_018266394; length 251 nt) forward– GTA CAT GCG CTT ACG GCA AC, reverse– AGG CTA CAG CAG TGA GTG TT; IDH1.L (NCBI Accession number NM-001094553; length 250 nt) forward– AAC GCC AGG ATG TCC AAG AA, reverse– TCT CAT CAG GTG TAA TAG TGG CA; PRMT1.L (NCBI Accession number NM_001089302.1; length 275 nt) forward– GAG GCG AAG ACC TGC AAC AT, reverse– ACT TTC TTG GCA CCA GCC TT; SUZ12.S (NCBI Accession number NM_001130944; length 273 nt) forward– CTG TCA AAC CTG CAC AGA CAA, reverse– TGT CCT CTT TGG TCA CAT AGT TG; JARID2.L (NCBI Accession number BC086634.1; length 258 nt) forward – TGT GTT TTG CTT GGA GTG TGC, reverse – CAG GAT GAA GCA CTT TTG GAC A. Except for *socs3*, T7 RNA polymerase promoter sequences were added to the 5′ end of reverse primers for in vitro transcription directly from PCR products. PCR product sizes were confirmed on 1% agarose gels, and the products further purified (Monarch PCR and DNA Cleanup Kit, New England Biolabs). The *socs3* RT-PCR product was cloned into a plasmid having a T7 promoter [107] for in vitro transcription. Digoxigenin-labeled antisense cRNA probes were generated by in vitro transcription (DIG RNA Labeling Kit (SP6/T7); Roche-Millipore Sigma).

For in situ hybridization, (3) fully anesthetized frogs (0.1% MS-222) were intracardially perfused first with Ringer’s solution containing MS-222 (112 mM NaCl, 2 mM KCl, 1 mM CaCl_2_, 1.2 mM NaHCO_3_, 0.1% MS-222, pH 7.4) and then with 4% paraformaldehyde in 0.1 M sodium phosphate (pH 7.4; PB) for fixation. Heads were then removed, cryoprotected in increasing concentrations of sucrose solutions (10, 20, 30%) in PB, then embedded in Tissue Freezing Medium (Triangle Biomedical Sciences) and cryo-sectioned (Leica CM1950) to obtain 18 μm thick transverse sections, which contained both eyes. These were thaw-mounted onto (+)-charged glass slides (Tissue Tak, Polysciences), which were subsequently stored at − 20 °C. For staining, slides were post-fixed in 4% paraformaldehyde in PB (20 min; 4 °C), then treated with 10 μg/ml proteinase K (Sigma-Aldrich; 6 min; 37 °C) and re-fixed in 4% paraformaldehyde (20 min, 4 °C). Slides were then incubated in 0.2 M HCl (30 min, room temperature) to inactivate endogenous alkaline phosphatase. After several washes in PB, slides were treated with 0.1 M triethanolamine (pH 8)/0.25% acetic anhydride, to further block non-specific hybridization, and subsequently washed in 2x saline-sodium citrate (SSC) buffer. Following a pre-hybridization incubation [50% formamide, 1x Denhardts, 20 mM tris (pH 7.4), 1 mM EDTA, 0.3 M NaCl, 0.1 g/ml dextran sulfate, 0.1 mg/ml herring sperm DNA, and 0.25 mg/ml yeast RNA], slides were hybridized with digoxigenin-labeled cRNA probes (100 ng in 200 μl/slide, 20 h, 50 °C). After hybridization, slides were treated with RNase A (20 μg/ml RNase A, 0.5 M NaCl, 0.25 mM EDTA, and 10 mM tris, pH 8) to further remove unhybridized probe. Slides were next washed in 2x SSC (room temperature), followed by a high stringency wash in 0.1x SSC (65 °C). Digoxigenin signals were detected by immunostaining with alkaline phosphatase-conjugated Fab fragments (Roche-Millipore Sigma), then developed with 4-nitro blue tetrazolium chloride/5-bromo-4-chloro-3-indoyl phosphate (Roche Millipore Sigma). The stained sections were then mounted under #1 coverglass in Fluoromount G (ThermoFisher Scientific). They were subsequently imaged using a Leitz Laborlux S (25X PL Fluotar, 0.6NA) microscope and a Nikon DS-Ri1 camera, using NIS-Elements BR 4.5 software (Nikon).

## Supplementary information

**Additional file 1. **Cuffdiff2 differential expression data. Tabs are for each injury condition and time point vs. their relevant controls (*N* = 3 biological replicates, each). **Tabs PW1–3)** Tadpole spinal cord injury (SCI) hindbrain vs. age-matched, unoperated control hindbrain at 3 days, 1 week, and 3 weeks, respectively; **PW4–6)** Juvenile frog SCI hindbrain at 3 days, 1 week, and 3 weeks vs. unoperated control hindbrain; **PW7–9)** juvenile frog optic nerve crush (ONC) eye vs. the contralateral unoperated eye at 3 days, 11 days, and 3 weeks, respectively. Abbreviations: chr, chromosome; cntrl, control; FPKM, fragments per kilobase of transcript per million mapped reads; ONC, optic nerve crush; *p*, the calculated probability for a single given gene that the observed difference represented no change in expression (i.e., log_2_(fold change) = 0); *q*, false discovery rate-adjusted (FDR) *p*-value [14]; significant, “yes” if q < 0.05; Tx, spinal cord-transected; Unop, unoperated.

**Additional file 2. **DESeq2 differential expression data. Tabs are for each injury condition and time point vs. their relevant controls (N = 3 biological replicates, each). **Tabs PW1–3)** Tadpole spinal cord injury (SCI) hindbrain vs. age-matched, unoperated control hindbrain at 3 days, 1 week, and 3 weeks, respectively; **PW4–6)** Juvenile frog SCI hindbrain at 3 days, 1 week, and 3 weeks vs. unoperated control hindbrain; **PW7–9)** juvenile frog optic nerve crush (ONC) eye vs. the contralateral unoperated eye at 3 days, 11 days, and 3 weeks, respectively. Columns: A - Gene Name; B, baseMean - mean counts of all samples, normalized for sequencing depth. It does not take into account gene length and is used for estimating the dispersion of a gene; C, log2FoldChange - log_2_(control/experimental); in this analysis, negative values represent injury induced increases relative to control); D, lfcSE - Standard Error of the log_2_ fold change; E, stat - log_2_FoldChange/lfcSE; F, pvalue - the probability of the null hypothesis for an individual gene (i.e., variance among replicates = variance between conditions); G, padj - false discovery rate-adjusted (FDR) *p*-value.

**Additional file 3. **Temporal patterns of gene expression and shared injury-response genes between regenerative vs. non-regenerative tissues, as determined by DESeq2. **A)** Regenerative tissues [i.e.*,* SCI tadpole hindbrain (SCI Tadpole) and ONC juvenile frog eye (ONC Juvenile)] shared similar temporal patterns of numbers of significant (FDR < 0.05) differentially expressed genes, which differed markedly from that of the non-regenerative tissue [SCI juvenile frog hindbrain (SCI Juvenile)]. Whereas the expression response of the two regenerative tissues peaked during the mid recovery phase (1 week/11 days), that of the non-regenerative tissue peaked at the early, post trauma phase (3 days). Up- and down-regulated genes are shown in green and red, respectively; S & L gene homeologs were tallied separately. **B)** Plot illustrating the percentage of annotated genes that were significantly (FDR < 0.05) differentially expressed with injury (100% = 24,382 genes). Additional_File2_Differential_Expression_Analysis_by_DESeq2.xlsm contains the DESeq2 output files from which A and B were derived. **C - E)** UpSet plots illustrating the number of genes overlapping between the samples indicated by the circles below each bar at 3 days (**C**), 7/11 days (**D**), and 3 weeks (**E**) after injury. Numbers of shared up- and down-regulated genes are indicated above and below each bar, respectively. The maximum number of overlapping genes between the two successfully regenerative tissues (DESR: Differentially Expressed in Successful Regeneration) occurred during the peak phase of regenerative CNS axon outgrowth.

**Additional file 4. **Manually Curated DESR Gene Data (Cuffdiff2). DESR gene lists were manually curated and parsed into eleven functional categories using a literature search. **Tabs: 3 Day DESR, 11 Day 1 WK DESR, 3 WK DESR:** Manually curated spreadsheets for each time point, 3 days, 11 days/1 week, and 3 weeks, respectively, providing Cuffdiff2 data for each gene for tadpole SCI hindbrain (even numbered rows) and juvenile frog ONC (odd numbered rows). **Column A**, surgical condition; **Columns B - N,** relevant Cuffdiff2 data for each gene under each surgical condition (see Additional_File1_Differential_Expression_Analysis_by_Cuffdif.xlsm for abbreviations and column annotations). Gene symbols (**Column B**) in green and red were up-regulated and down-regulated, respectively, in successfully regenerative tissues relative to controls. **Column O,** primary functional category (NA, gene has not been assigned a human ortholog); **Column P,** secondary functional category, if relevant; **Column Q,** Manually curated notes, providing additional information on the expression behaviors of the other homeologs of this gene (even numbered rows), and a statement on the function of the gene that lead to its being placed in a particular functional category, followed by the literature reference supporting it.

**Additional file 5. **Scatterplot representation of the Principal Component Analyses (PCA) of gene expression profiles. Ellipses group biological replicates for each indicated condition (experimentals, solid squares; controls, empty squares), indicating variation among samples. **A-C,** PCA of tadpole and juvenile hindbrain after spinal cord injury (SCI), and of juvenile frog after optic nerve crush (ONC), respectively. In C, expression profiles from the operated eye were compared with those of the contralateral, unoperated eyes within the same animals. **D,** PCA of all 17 conditions combined, supporting the tissue-specific nature of gene expression profiles. Conditions were the same as in A-C, except that data from eyes of animals receiving no injury was included (open triangles, Frog Eye, Unop). **E,** PCA of tadpole and juvenile frog hindbrain samples after spinal cord injury, supporting the conclusion that the differences in gene expression profiles between the time points at which numbers of differentially expressed genes reached their peaks (3 days in juvenile frog hindbrain and 1 week in tadpole hindbrain) were more than just a kinetic difference in the timing of expression of the same differentially expressed genes. Abbreviations: ONC, optic nerve crush; PC1, principal component axis 1; PC2, Principal Component axis 2; SCI, spinal cord injured; TX, spinal cord transected; Unop ONC - unoperated eye, contralateral to the operated eye; Frog Eye, Unop - eyes from unoperated animals; wk., week.

**Additional file 6.** A summary list of all DESR genes, sorted by primary functional category and time point of differential expression, along with relevant references supporting the functional assignment. Note that genes were assigned their human gene ortholog symbols, without reference to whether they were L or S homeologs, or paralogs.

**Additional file 7. **KEGG pathway analysis of the DESR gene lists revealed that 60% (69/115) of the Adipocytokine signaling pathway genes were differentially expressed in at least one tissue at one time point (*p* < 0.02). **Tab. 1, Pathway:** the Adipocytokine Signaling Pathway (obtained from KEGG) and the complete associated gene list; **Tab. 2, Gene Expression Levels:** Column B provides the list of genes and their synonyms that are in this KEGG pathway (the underlined name is the one provided in the figure in Tab. 1. Column C provides the gene symbol and tissue for each gene. ΔFPKM [log_2_(fold change), injury condition/control, Column D] for each differentially expressed gene in the pathway (115 genes, S & L homeologs listed separately), as well as for other isoforms for each gene. Columns D – L, the ΔFPKMs for each tissue at all three time points are listed. For significant changes, either by *p* or *q* (or both), and the raw FPKMs are listed in a cell comment for the ΔFPKMs. The meanings of the different color highlights is given in the legend at the top of the table. **Tab. 3, Genes Not Found:** is a list of 10 genes (including synonyms) from the KEGG adipocytokine signaling pathway that were not found in the Mayball annotations of the *X. laevis* transcriptome. Abbreviations are as in Additional_File1_Differential_Expression_Analysis_by_Cuffdif.xlsm.

## Data Availability

The datasets generated and/or analyzed during the current study are available either in the Gene Expression Omnibus (GEO) repository (FASTQ files) [https://www.ncbi.nlm.nih.gov/gds]^[GSE 137844]^ or are included in this published article and its supplementary information files. Genomic resources for *Xenopus laevis* are available from Xenbase (http://www.xenbase.org, RRID:SCR_003280) and *Xenopus* Genome Project (http://www.marcottelab.org/index.php/Xenopus_Genome_Project).

## Abbreviations

CNS: Central nervous system
DESR: Differentially expressed in successful regeneration
FDR: False discovery rate
FPKM: Fragments per Kilobase Million
KEGG: Kyoto Encyclopedia of Genes and Genomes
Mya: million years ago
ONC: Optic nerve crush
ONI: Optic nerve injury
PCA: Principal component analysis
PNS: Peripheral nervous system
SCI: Spinal cord injury
*X. laevis*: *Xenopus laevis*
