# Supplementary material for: Comparative gene expression profiling between optic nerve and spinal cord injury in Xenopus laevis reveals a core set of genes inherent in successful regeneration of vertebrate central nervous system axons
Source: BMC Genomics. 2020 Aug 5;21:540. doi: 10.1186/s12864-020-06954-8 (PMC7430912; doi:10.1186/s12864-020-06954-8)
Supplement: Supplementary file 3 — Additional file 3. Temporal patterns of gene expression and shared injury-response genes between regenerative vs. non-regenerative tissues, as determined by DESeq2. A) Regenerative tissues [i.e., SCI tadpole hindbrain (SCI Tadpole) and ONC juvenile frog eye (ONC Juvenile)] shared similar temporal patterns of numbers of significant (FDR < 0.05) differentially expressed genes, which differed markedly from that of the non-regenerative tissue [SCI juvenile frog hindbrain (SCI Juvenile)]. Whereas the expression response of the two regenerative tissues peaked during the mid recovery phase (1 week/11 days), that of the non-regenerative tissue peaked at the early, post trauma phase (3 days). Up- and down-regulated genes are shown in green and red, respectively; S & L gene homeologs were tallied separately. B) Plot illustrating the percentage of annotated genes that were significantly (FDR < 0.05) differentially expressed with injury (100% = 24,382 genes). Additional_File2_Differential_Expression_Analysis_by_DESeq2.xlsm contains the DESeq2 output files from which A and B were derived. C - E) UpSet plots illustrating the number of genes overlapping between the samples indicated by the circles below each bar at 3 days (C), 7/11 days (D), and 3 weeks (E) after injury. Numbers of shared up- and down-regulated genes are indicated above and below each bar, respectively. The maximum number of overlapping genes between the two successfully regenerative tissues (DESR: Differentially Expressed in Successful Regeneration) occurred during the peak phase of regenerative CNS axon outgrowth. [file 12864_2020_6954_MOESM3_ESM.pdf]

Additional_File3_DESeq2_Metadata.pdf. Temporal patterns of gene expression and shared injury-response genes between regenerative vs. non-regenerative tissues, as determined by DESeq2. **A)** Regenerative tissues [*i.e.,* SCI tadpole hindbrain (SCI Tadpole) and ONC juvenile frog eye (ONC Juvenile)] shared similar temporal patterns of numbers of significant (FDR < 0.05) differentially expressed genes, which differed markedly from that of the non-regenerative tissue [SCI juvenile frog hindbrain (SCI Juvenile)]. Whereas the expression response of the two regenerative tissues peaked during the mid recovery phase (1 week/11 days), that of the non-regenerative tissue peaked at the early, post trauma phase (3 days). Up- and down-regulated genes are shown in green and red, respectively; S & L gene homeologs were tallied separately. **B)** Plot illustrating the percentage of annotated genes that were significantly (FDR < 0.05) differentially expressed with injury (100% = 24,382 genes). Additional_File2_Differential_Expression_Analysis_by_DESeq2.xlsm contains the DESeq2 output files from which A and B were derived. **C** – **E)** UpSet plots illustrating the number of genes overlapping between the samples indicated by the circles below each bar at 3 days (**C**), 7/11 days (**D**), and 3 weeks (**E**) after injury. Numbers of shared up- and down-regulated genes are indicated above and below each bar, respectively. The maximum number of overlapping genes between the two successfully regenerative tissues (DESR: Differentially Expressed in Successful Regeneration) occurred during the peak phase of regenerative CNS axon outgrowth.

DESeq2 Metadata

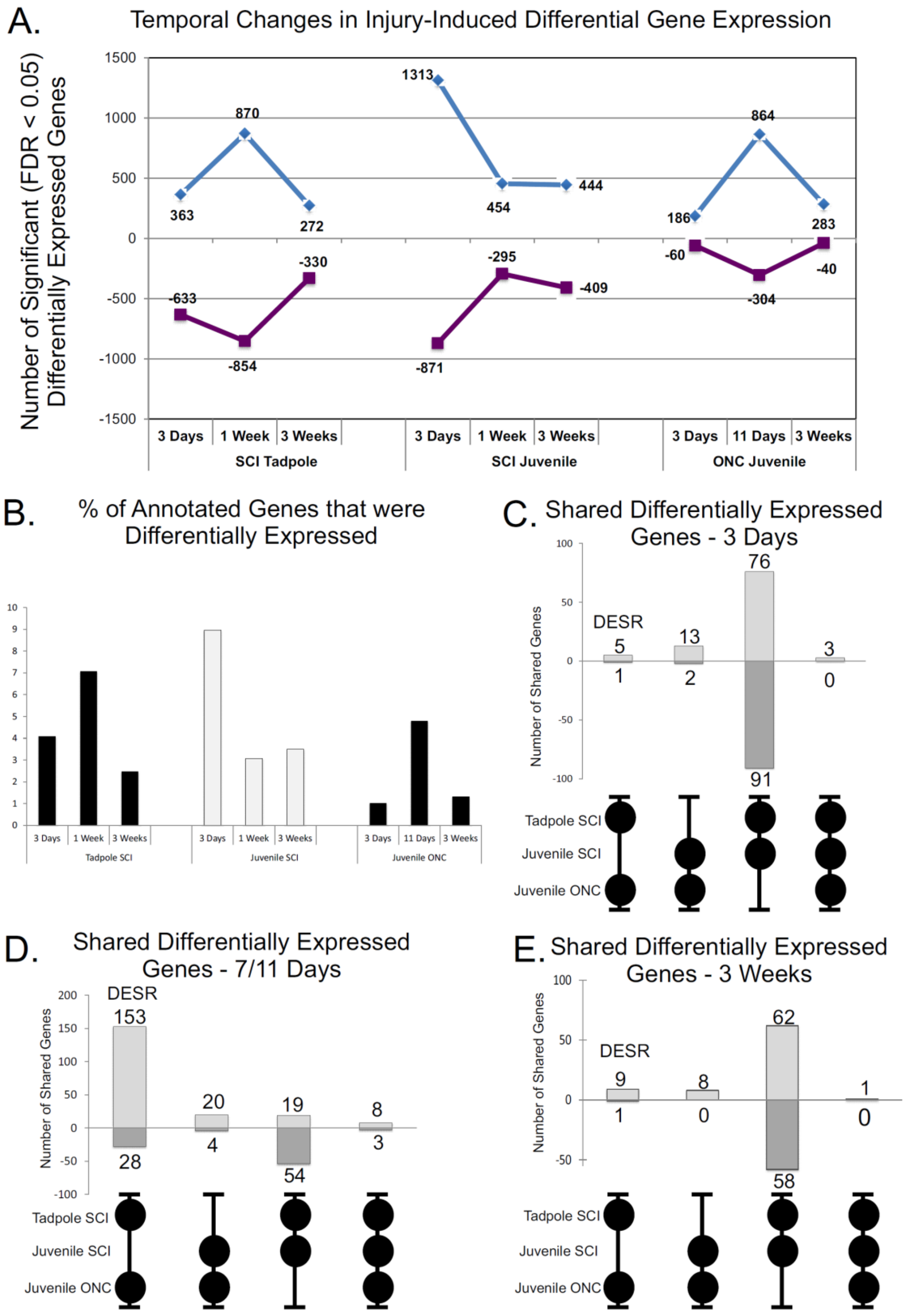

Supplemental Data Figure
