# Supplementary material for: Comparative gene expression profiling between optic nerve and spinal cord injury in Xenopus laevis reveals a core set of genes inherent in successful regeneration of vertebrate central nervous system axons
Source: BMC Genomics. 2020 Aug 5;21:540. doi: 10.1186/s12864-020-06954-8 (PMC7430912; doi:10.1186/s12864-020-06954-8)
Supplement: Supplementary file 5 — Additional file 5. Scatterplot representation of the Principal Component Analyses (PCA) of gene expression profiles. Ellipses group biological replicates for each indicated condition (experimentals, solid squares; controls, empty squares), indicating variation among samples. A-C, PCA of tadpole and juvenile hindbrain after spinal cord injury (SCI), and of juvenile frog after optic nerve crush (ONC), respectively. In C, expression profiles from the operated eye were compared with those of the contralateral, unoperated eyes within the same animals. D, PCA of all 17 conditions combined, supporting the tissue-specific nature of gene expression profiles. Conditions were the same as in A-C, except that data from eyes of animals receiving no injury was included (open triangles, Frog Eye, Unop). E, PCA of tadpole and juvenile frog hindbrain samples after spinal cord injury, supporting the conclusion that the differences in gene expression profiles between the time points at which numbers of differentially expressed genes reached their peaks (3 days in juvenile frog hindbrain and 1 week in tadpole hindbrain) were more than just a kinetic difference in the timing of expression of the same differentially expressed genes. Abbreviations: ONC, optic nerve crush; PC1, principal component axis 1; PC2, Principal Component axis 2; SCI, spinal cord injured; TX, spinal cord transected; Unop ONC - unoperated eye, contralateral to the operated eye; Frog Eye, Unop - eyes from unoperated animals; wk., week. [file 12864_2020_6954_MOESM5_ESM.pdf]

Additional_File5_PCA_Scatterplot.pdf. Scatterplot representation of the Principal Component Analyses (PCA) of gene expression profiles. Ellipses group biological replicates for each indicated condition (experimentals, solid squares; controls, empty squares), indicating variation among samples. **A–C,** PCA of tadpole and juvenile hindbrain after spinal cord injury (SCI), and of juvenile frog after optic nerve crush (ONC), respectively. In C, expression profiles from the operated eye were compared with those of the contralateral, unoperated eyes within the same animals. **D,** PCA of all 17 conditions combined, supporting the tissue-specific nature of gene expression profiles. Conditions were the same as in A–C, except that data from eyes of animals receiving no injury was included (open triangles, Frog Eye, Unop). **E,** PCA of tadpole and juvenile frog hindbrain samples after spinal cord injury, supporting the conclusion that the differences in gene expression profiles between the time points at which numbers of differentially expressed genes reached their peaks (3 days in juvenile frog hindbrain and 1 week in tadpole hindbrain) were more than just a kinetic difference in the timing of expression of the same differentially expressed genes. Abbreviations: ONC, optic nerve crush; PC1, principal component axis 1; PC2, Principal Component axis 2; SCI, spinal cord injured; TX, spinal cord transected; Unop ONC – unoperated eye, contralateral to the operated eye; Frog Eye, Unop – eyes from unoperated animals; wk, week.

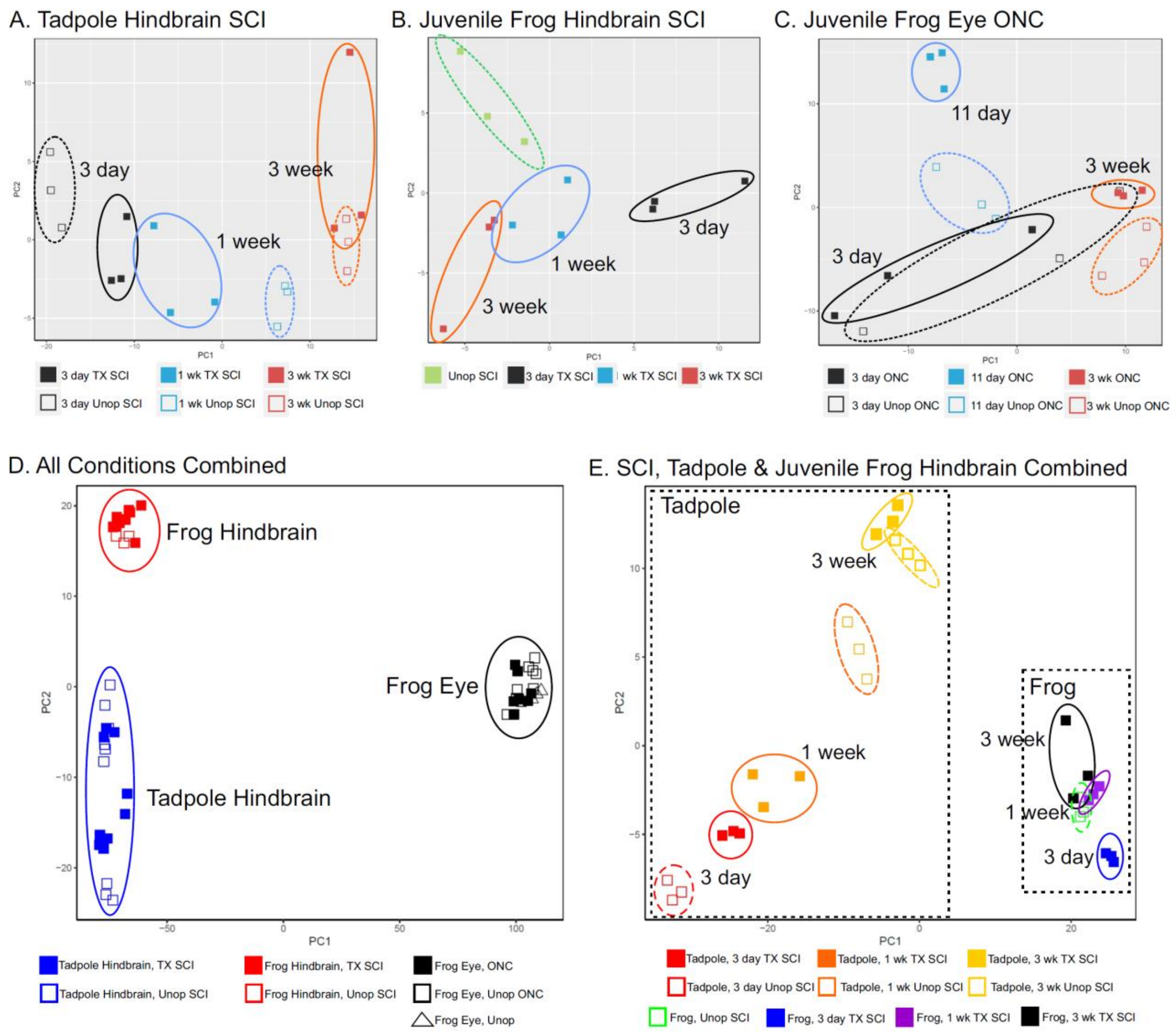

A. Tadpole Hindbrain SCI
3 day
1 week
3 week
3 day TX SCI
1 wk TX SCI
3 wk TX SCI
3 day Unop SCI
1 wk Unop SCI
3 wk Unop SCI
B. Juvenile Frog Hindbrain SCI
3 day
1 week
3 week
Unop SCI
3 day TX SCI
wk TX SCI
3 wk TX SCI
C. Juvenile Frog Eye ONC
11 day
3 week
3 day
3 day ONC
11 day ONC
3 wk ONC
3 day Unop ONC
11 day Unop ONC
3 wk Unop ONC
D. All Conditions Combined
Frog Hindbrain
Frog Eye
Tadpole Hindbrain
PC1
PC2
Tadpole Hindbrain, TX SCI
Tadpole Hindbrain, Unop SCI
Frog Hindbrain, TX SCI
Frog Hindbrain, Unop SCI
Frog Eye, ONC
Frog Eye, Unop ONC
Frog Eye, Unop
E. SCI, Tadpole & Juvenile Frog Hindbrain Combined
Tadpole
3 week
1 week
3 day
Frog
3 week
1 week
3 day
PC1
PC2
Tadpole, 3 day TX SCI
Tadpole, 1 wk TX SCI
Tadpole, 3 wk TX SCI
Tadpole, 3 day Unop SCI
Tadpole, 1 wk Unop SCI
Tadpole, 3 wk Unop SCI
Frog, Unop SCI
Frog, 3 day TX SCI
Frog, 1 wk TX SCI
Frog, 3 wk TX SCI
