# Supplementary material for: Comparative gene expression profiling between optic nerve and spinal cord injury in Xenopus laevis reveals a core set of genes inherent in successful regeneration of vertebrate central nervous system axons
Source: BMC Genomics. 2020 Aug 5;21:540. doi: 10.1186/s12864-020-06954-8 (PMC7430912; doi:10.1186/s12864-020-06954-8)
Supplement: Supplementary file 6 — Additional file 6. A summary list of all DESR genes, sorted by primary functional category and time point of differential expression, along with relevant references supporting the functional assignment. Note that genes were assigned their human gene ortholog symbols, without reference to whether they were L or S homeologs, or paralogs. [file 12864_2020_6954_MOESM6_ESM.pdf]

Appendix: Genes Implicated by Expression Analysis to be Involved in Successful CNS Axon Regeneration: Cellular Functions, Time of Differential Expression, and Key References

1. Inflammatory Response and Wound Healing – *functions in regulating inflammation, cell death/survival, stress and innate immune responses, wound healing, and microglial/myeloid cell activity*

Summary: This was largest DESR gene category, comprising functions related to regulating inflammation, innate immunity, wound healing, and cell survival responses, as well as tissue repair and regeneration. It totaled 50%, 22%, and 60% of all DESR genes at the early, middle, and late time points, respectively. Inflammation is crucial for initiating and maintaining pro-regenerative responses to injury [16], but because inflammation also damages tissues, distinguishing inflammation-related pro-regenerative genes from those detrimental to recovery has proved challenging in mammalian studies. These genes can provide insights into which are likely pro-regenerative vs. detrimental by comparing those that were up-regulated under the two regenerative conditions vs. those that were downregulated, respectively. At 3 days, DESR genes were dominated by increased expression of genes typically considered pro-inflammatory (*leptin*, *C9*, *ecm1, ddit3*), along with genes associated with increased activity and tissue penetrance of macrophages (*ceacam8, efemp1 epx*), and activation of JAK/STAT signaling through cytokine receptors (*mmp2, mmp13, socs3*). The two down-regulated DESR genes at 3 days were a cytokeratin associated with wound sites in mammals (*krt6a*) and a pro-inflammatory, calcium-binding protein secreted by macrophages (*ocm2*). At the peak phase of regenerative axon outgrowth, there were nine up-regulated pro-inflammatory genes [two holdovers from 3 days (*leptin, C9*) and 7 new ones (*ubclp1, hbe1, gng7 tmem2, fcrl4, lgals8, mst1*)], plus 32 genes that have been previously associated with promoting cell survival, tissue repair and regeneration, with dampening inflammation, and with mediating a stress response. Eight additional up-regulated genes were associated with myeloid cell activities, including three that were known to promote the transition from pro-inflammatory M1 to repair-promoting M2 macrophages (*lta4h, hmox1, mmp28*). Conversely, the down-regulated DESR genes at this time included ten genes previously associated with exacerbation of inflammation, cell death, and scar formation (*e.g.*, *enpp2, znf395, cal14a1*), along with six genes associated with oxidative stress (*scara3, higd1c*), myeloid cell activity (*ms4a4a*, *rasgrp3*) and maintaining the blood-brain barrier (*mxra8*), plus a heat-shock protein (*hspa8*). At the late time point (3 weeks), all nine DESR genes in this category were up-regulated genes previously implicated in protecting cells from detrimental aspects of inflammation and in promoting cellular repair and regeneration (*slc44a2*, *a2m, ifr8, syt11, cfh, plat, ifitm3, mst1,* and *ltf*).

3 days

Up-regulated[1]

Pro-Inflammatory Molecules –

C9*[,3]	The final component of the complement cascade [172; 198; 257].
CEACAM8	Adhesion molecule that increases vascular permeability to promote tissue invasion by macrophages and other myeloid cells [318].
DDIT3	(See also Transcription Factors) A C/EBP-related transcription factor that functions primarily as a transcriptional repressor by forming inactive partners with other C/EBP transcription factors; it is regulated by stress factors and is important for the activation of pro-inflammatory signals [239].
EFEMP1	A membrane protein that promotes macrophage migration [67].
ECM1	An activator of the C3 alternative complement cascade [33].
EPX	An enzyme that stimulates production and release of hydrogen peroxide and hypohalous acid, which act as macrophage chemoattractants [127; 128].
LEP[*]	Cytokine receptor ligand and activator of JAK/STAT signaling [11].

JAK/STAT-Activated Regulators of Inflammation and Wound Healing –

MMP2	A matrix metalloprotease that promotes CNS axon regeneration and axon guidance [204; 240; 256; 309]
MMP13	A matrix metalloprotease that regulates scarring [66]

SOCS3* A negative modulator of JAK/STAT signaling, which also has pro-regenerative effects [[201; 202], and [15] for review].

Down-regulated[2]

Cytokeratin Associated with Wound Healing –

KRT6A (See also Cytoskeletal, below) A cytokeratin associated with wound sites; in rodents, *krt6a* knockout increases epithelial wound site fragility [320].

Inflammatory Cell-secreted Factor –

OCM2 In rodents, oncomodulin stimulates optic axon regeneration [147; 330; 331].

1 wk/11 days

Up-regulated

Pro-Inflammatory Molecules –

HBE1[4] A major component of embryonic hemoglobins in mammals, with pro-inflammatory properties [86].

LEP* see LEP at 3 days.

C9* see C9 at 3 days.

UBLCP1 A ubiquitin-like-domain-containing phosphatase [311].

GNG7 A G-protein that plays a role in CCR3 signaling in eosinophils in stroke [95].

TMEM2 An interferon family member that encodes a hyaluronidase and activates JAK/STAT signaling [348].

FCRL4 A marker for pro-inflammatory B cells [329].

LGALS9 A lectin that promotes tissue invasion by inflammatory myeloid cells [22; 208].

MST1 A Hippo-protein that stimulates macrophages [344; 345].

Protein Ubiquitination and Turnover – linked to suppressing socs3's anti-regenerative effects [251; 290].

UBE2D4 A ubiquitin conjugating enzyme E2, also *see PSMA4.*

UCHL1 A ubiquitin esterase [88]; *also see PSMA4.*

FBXO2 A component of the neuronal ubiquitinating SCF complex [10].

PSMA4 A component of the proteasome [170; 290].

PSMA3 *see PSMA4.*

DKFZP686D09174 A proteasomal *psma2* homolog implicated in axon regeneration [87].

PSMB5 *see PSMA4.*

PSMB7 *see PSMA4.*

PSMC1 *see PSMA4.*

PSMC6 *see PSMA4.*

PSMD12 *see PSMA4.*

Chaperones –

CCT2 A chaperone that promotes folding of actin and tubulin and also plays a role in autophagy [244].

CRYAB A generally neuroprotective, potent inhibitor of inflammation [103].

CRYGB A stimulator of JAK/STAT signaling, macrophage activity, and axon regeneration [68].

AHSA1	A co-chaperone for HSP90AA1, involved in cellular stress response to MAP-tau aggregates [272].

Protective Genes that Promote Cell Survival, Tissue Repair and Regeneration –

FADS1	(See also Lipid Metabolism) A fatty acid desaturase that is directly implicated in down-regulating inflammation [92].
LTF*	A neuroprotective gene in Parkinson's disease [262]; see also 3 wk.
OTOP3	A member of a gene family that suppresses inflammation and promotes tissue repair [308].
ANXA5	Implicated in suppressing inflammation; it is upregulated in amphibian limb regeneration where it inhibits cell death and inflammation [53; 136; 327].
CASP9	Although typically thought of as an initiator of apoptosis, it also plays a role as an activator of axon guidance molecules [230; 233; 282].
CXCR4	A chemokine receptor associated with neuronal stem cell differentiation and axon guidance [258].
PLAT*	A neuroprotective modulator of inflammation [90; 90; 100], see also 3 weeks.
SOCS3*	Also at 3 days. In mammals, it is generally considered pro-inflammatory, but see [201; 251].
SLC25A1	(See also Intracellular Transport) Involved in transporting citrate across mitochondrial membranes. It regulates cellular metabolism and TP53 responses, and its expression is triggered by STAT. It is essential for NO and prostaglandin production in the inflammatory response [113].
TXN	Involved in protecting neurons from oxidative stress [156].

Stress Response Genes, Including Mitochondrial Response to Oxidative Stress –

MAPK8	A stress response kinase required for regenerative and developmental axon outgrowth, regulating both the mRNA translation and axonal transport of key cytoskeletal structural proteins needed for building the axon [112; 231].
HBD	Primarily known as a carrier of oxygen in red blood cells, but in other cells, it also plays a role in managing oxidative stress [50].
SH3BGRL3	A thioredoxin-like molecule that helps cells resist damage from free radicals [324].
TMEM14C	Essential gene for mitochondrial heme metabolism, implicated in autoimmune diseases such as multiple sclerosis [2].
AK2	A mitochondrial AKT kinase, generally considered protective for cells [206].
CASP3	Activates apoptosis, but is also neuroprotective in the presence of HSP70 and HSC70 [197].
PRDX1	Regulates cellular hydrogen peroxide levels in cells to protect them from oxidative stress, *e.g.*, [114].

Other Myeloid Cell-associated Genes –

SSR3	Associated with protein translocation across the ER membrane; it is upregulated in T-cells upon exposure to IL-2 [60].
RTKN2	A Rho-GTPase target implicated in lymphopoiesis [42].
GCA	An abundant calcium binding protein in macrophages [325].
LY9	A gene expressed in lymphocytes that is involved in a wide range of adaptive and innate immune responses, including suppressing inflammation [18].

SSSCA1	A gene of unknown function, associated with autoimmune diseases; it may be part of the centromere of cells involved in the immune response [215].

Genes that Promote/Mark the M1/M2 Transition –

LTA4H	[49].
HMOX1	[218].
MMP28	A matrix metalloprotease implicated in peripheral nerve repair in *Xenopus* and in promoting the M1 to M2 transition [79; 317].

<u>*Down-regulated*</u>

Genes that Exacerbate Inflammation, Cell Death, and Scar Formation –

ENPP2[5]	A stimulator of inflammation in cancer [40; 137].
SLC9A3R2	A suppressor of STAT3 [336].
UGT8	(See also Lipid Metabolism) An important enzyme for sphingolipid metabolism, which is important for making myelin; elevated UGT8 promotes neuroinflammation in humans [339].
ZNF395	(a.k.a Huntington Disease Regulatory Region Binding Protein 2; see also Transcription Factors): A zinc-finger transcription factor that activates pro-inflammatory cytokines [102].
BCL6	(See also Transcription Factors) A transcriptional repressor of STAT3 [213].
COL14A1	A collagen subtype, implicated in scar formation in liver disease [269].
CYR61	An angiogenic inducer [122].
PPT2	(See also Lipid Metabolism) A critical enzyme in the synthesis of sphingosine, which is especially important for the synthesis of sphingolipids in myelin. Elevated sphingolipid levels promote neuro-inflammation, and PPT2 knockdown in mice leads to neurodegeneration [89; 281; 339].
PDZD2	A relative of IL-16, a pro-inflammatory cytokine [181].
TXNIP	A thioredoxin-interacting protein that promotes apoptosis in brain injury [222; 343].

Genes Associated with Oxidative Stress –

SCARA3	A macrophage scavenger receptor involved in oxidative stress [23].
HIGD1C	A mitochondrial protein induced by oxidative stress [5; 75].

Chaperones –

HSPA8	A constitutively expressed chaperone protein, related to HSP70 [349].

Other Myeloid Cell-associated Genes –

MS4A4A	A membrane protein with multiple splice forms associated with a variety of myeloid cells, including macrophages [265].
RASGRP3	A GEF that plays a role in limiting toll-like receptor-triggered inflammatory responses in macrophages [288].

Gene Associated with Maintaining the Blood Brain Barrier –

MXRA8	A cell adhesion molecule expressed in astrocytic endfeet lining the vasculature [8; 333].

3 wk

Up-regulated

Protective Genes that Promote Cellular Repair and Regeneration –

LTF	See 1 wk/11 days [146].
SLC44A2	A choline transporter associated with neutrophil activation and increased membrane synthesis for cell growth and repair [13; 294].
A2M	An inhibitor of fibrinolysis and disruptor of cytokine-induced inflammation, with neuroprotective qualities [207].
IRF8	(See also Transcription Factors) A transcription factor that responds to interferons to suppress the hyper-inflammatory response in macrophages [115].
SYT11	An inhibitor of cytokine secretion and phagocytosis by macrophages [57; 306].
CFH	A complement factor that redirects the C3 pathway toward pathogens and away from tissue damage [259].
PLAT*	See 1 wk/11 days [100].
IFITM3	An anti-inflammatory modulator of interferon signaling [161].
MST1	A hippo kinase that suppresses macrophage infiltration [160; 344].

Down-regulated

–*None*–

---

2. Cytoskeletal – *structural and regulatory functions associated with the cytoskeleton*

Summary: Except for three intermediate filament genes, cytoskeletal-related DESR genes comprised tubulin and actin subtypes and genes associated with regulating microtubule and microfilament transport and dynamics (*e.g., mylk, dynlt1, dynll2, kif20b, ttl*). This is understandable given the importance of these genes for axon outgrowth, intracellular transport, cell motility, and cellular proliferation (27 in total). Two of the intermediate filament genes were cytokeratins that were down-regulated at 3 days (*krt75* and krt6a). *Krt6a* has been associated previously with wound sites in mammals. Our data suggest its downregulation is important to promote regenerative healing. The remaining intermediate filament gene was differentially up-regulated at 3 weeks (prph). It has been previously reported to be upregulated at all stages of regeneration in optic axons and reactive astrocytes. Although its precise function remains unknown, its preferential up-regulation in successful vs. unsuccessful regeneration at the latest stage examined, suggests it retains this importance into these stages.

3 days

Up-regulated

–*None*–

Down-regulated

KRT75	A cytokeratin normally associated in mammals with hair follicles; one of many KRT75 homo/paralogs in *Xenopus* [14; 84].
ACTA1	The predominant form of actin in skeletal muscle [151].
ACTC1	Cardiac actin, but in *Xenopus* it is associated with a wider range of tissue types [209; 261].
KRT6A	(See also Inflammatory Response and Wound Healing; 3 days, above) A cytokeratin associated with wound sites; in rodents, *krt6a* knockout increases epithelial wound site fragility [320].

Appendix: Genes Implicated by Expression Analysis to be Involved in Successful CNS Axon Regeneration: Cellular Functions, Time of Differential Expression, and Key References

1 wk/ 11 days

Up-regulated

Tubulin Subtypes –

TUBB2B	A neuron-specific tubulin subtype in *Xenopus*, present in early developing axons [211].
TUBA3D	*see TUBB3, below.*
TUBA1A	Tubulin subtype essential for optic axon regeneration in zebrafish [301].
TUBA1B	Tubulin subtype involved in mitosis, but not essential for optic axon regeneration in zebrafish [301].
TUBB3	Principally a neuronal tubulin subtype, implicated in axonogenesis [205].
TUBB	*see TUBB3.*

Motor Proteins –

DYNLT1	A neuronal minus end-directed microtubule motor protein, involved in axonal retrograde transport [41].
KIF20B	A plus end-directed microtubule motor protein required for cortical neuron polarization and for morphogenesis [199].
DYNLL2	A minus end-directed motor protein component required for the retrograde axonal transport of proteasomes [145].
KIF11	A plus end microtubule motor protein required for mitosis, as well as for transport of proteins and vesicles from the Golgi to the cell surface. It also transports thyroid receptor bound to thyroid hormone to the nucleus. Inhibiting it in cultured DRG neurons promotes neurite outgrowth [168].

Other Microtubule-Associated Proteins –

TTL	An enzyme that restores tyrosines on de-tyrosinated tubulins; required for increasing levels of tyrosinated tubulin at injury sites and for retrograde axonal transport of pro-regenerative signals, such as activators of c-Jun [278].
TPX2	A microtubule-associated mitotic spindle protein required for mitosis and neurite outgrowth; it also regulates TP53 activity [123].
STMN2	A microtubule associated protein important for growth cone motility and maintenance of axons after injury [274].
CKAP2	A microtubule-associated microtubule-stabilizing protein [334].
NCK2	A SOCS3-regulated, SH2/3 domain-interacting adaptor protein involved in cytoskeletal reorganization [275].
TCTEX1D1	A minus end-directed microtubule motor protein, potentially involved in retrograde axonal transport [55; 150].
KIFC1	A minus end-directed microtubule motor protein, potentially involved in retrograde axonal transport, but also regulates mitotic chromosome dynamics and macrophage podosome dynamics [143].

Actin Subtypes –

ACTB	A non-muscle beta actin thought to play a role in gene transcription during axon outgrowth [35; 283].

Microfilament-Associated Proteins –

TAGLN2	An actin-associated protein that inhibits ARP2/3 nucleated branching of microfilaments [135].

MYL12B	A myosin light chain required for axon outgrowth [24; 237].
TMSB4X	An actin sequestering protein that plays a major role in tissue regeneration, perhaps by lowering levels of pro-inflammatory cytokines [285].
ARPC5	A subunit of the ARP2/3 complex, which is important for axon branching in *Xenopus* [173].

Actin/Tubulin Chaperone Proteins–

CCT4	Chaperone protein important for proper folding of actins and tubulins. CCT's may play a role in microtubule dynamics in growth cones, and misregulation is associated with neurodegeneration [244].
CCT5	*see CCT4.*
CCT6A	*see CCT4.*
CCT6A	*see CCT4.*
TCP1	A chaperone protein essential for actin dynamics in the healing of wounded imaginal discs in fly [3].
PFDN4	A chaperone protein important for actin and tubulin synthesis with possible nuclear functions [203].

Down-regulated

Regulators of the Actin cytoskeleton–

MYLK	Myosin Light Chain Kinase essential for growth cone motility and cytokinesis; increased MYLK activity inhibits axon retraction [73; 119].
ANK1	An ankyrin protein, which links plasma membrane proteins to the underlying actin cytoskeleton [307].
ANKRD26	A second ankyrin-related protein associated with obesity and with defects in primary cilia in regions of the brain that control appetite and energy homeostasis [1].
SHANK2	(Also, Cell Signaling) A third ankyrin-related protein implicated in a wide range of plasticity disorders, including synapse development through Wnt signaling [96; 273].

3 wk

Up-regulated

EBF3	(See also Transcription Factors) A protein that binds microtubules to induce bundling, but also acts as a transcription factor to regulate cell survival, regulating genes involved in cell cycle arrest and apoptosis. For example, it inhibits glial cell proliferation and glioma-genesis [25; 164].
PRPH	In *Xenopus*, a principal intermediate filament protein of regenerating optic and peripheral axons, as well as reactive astrocytes [78; 191].
MAPT	Tau, a major axonal microtubule-associated protein associated with stabilizing microtubules [174; 279].

Down-regulated

–None–

---

3. Cell Signaling – *functions in cell signaling pathways and in synaptic transmission*

Summary: All the DESR genes in this category were differentially expressed at the peak phase of regenerative axon outgrowth. Up-regulated genes directly involved in receptor function included a cholinergic receptor (*chrng*), two ligands to Notch (*dll1*) and GABA receptors (*dbi*), and a range of modulators of cell signaling pathways, including Wnt, BMP, and G-protein coupled receptors. Several

additional genes are involved in intracellular aspects of cell signaling, including three kinases that regulate cell division (*plk1*), *tp53*-related functions (*aurk8*), and the synthesis and transport of axonal cytoskeletal proteins (*mapk8*), as well as two phosphatases associated with regulating cell division and axonal microtubule dynamics (*ppp2t1a,* and *ppp2r2a*). Down-regulated genes included multiple additional kinases (*e.g., adck3, rock2, erbb4, insr,* and others), a receptor tyrosine phosphatase (*ptprb*), a dopamine receptor-interacting GEF (*dock10*) and seven modulators of various signaling pathways [*i.e.,* calcium-related, phosphotidyl insositol-related, Wnt-related, and GPCR-related signaling]. In addition to these were five down-regulated ion channels (*kcnn3, kcnh5, grik2, clcn2,* and *kcnj2*), a sub-category not seen among the upregulated cell-signaling DESR genes.

3 days

Up-regulated

–*None*–

Down-regulated

–*None*–

1 wk/ 11 days

Up-regulated–

Kinases

PLK1	A polo-like kinase that regulates cell division [266].
AURKB	An Aurora kinase that regulates TP53 activity, microtubules, and intermediate filaments (vimentin) dynamics, as well as histone H3 during mitosis [91; 129].
MAPK8	(Also listed under Inflammatory Response and Wound Healing) A stress response kinase (also known as JNK) required for regenerative and developmental axon outgrowth, regulating both the mRNA translation and axonal transport of key cytoskeletal structural proteins needed for building the axon [112; 231].

Phosphatases–

PPP2R1A	A subunit of one of the major cellular serine/threonine protein phosphatases; generally associated with negative control of mitosis and cell growth, and more specifically with the dephosphorylation of MAP-tau [346].
PPP2R2A	A regulatory subunit of a major cellular phosphatase involved in negative control of cell growth and division and in regulating MAP-tau dynamics [287].

Receptors–

CHRNG	A subunit of the nicotinic cholinergic receptor, which in the CNS regulates communication between neurons and astrocytes in response to viral infection [169].

Receptor Ligands–

DLL1	A canonical Notch ligand that is broadly involved in tissue differentiation, neural crest development, and activation of stromal macrophages [32; 221].
DBI	An endogenous benzodiazepam receptor ligand, implicated in successful peripheral axon regeneration in mammals through its effects on steroidogenesis [149].

Intracellular/Transmembranous Cell Signaling Pathway Mediators and Modulators–

SDCBP	A membrane and nuclear protein that binds syndecan; associated with neuro and immunomodulation, and promotes spinal cord axon regeneration in zebrafish [338].
MEMO1	A protein that links cell membrane signaling (*e.g.*, ERBB2 and IGF1R) with microtubule dynamics [120].
COMT	A major enzyme mediating catecholamine neurotransmitters degradation with functions in neuronal synaptic plasticity, e.g., see [99].

(Wnt-related)

TMEM88	An inhibitor of the canonical Wnt/beta-catenin signaling pathway [234].

(BMP-related)

TMEM221	(a.k.a. Jiraiya) A transmembrane protein that attenuates BMP signaling in *Xenopus* development and interacts with multiple miRNAs [9].

(G-protein-related)

GNG3	A heterotrimeric G protein gamma subunit that regulates chemokine signaling in lymphocytes [132].

(14-3-3 protein related)

YWHAB	A 14-3-3 protein that suppresses apoptosis and mediates signal transduction for kinases and phosphatases associated with mitosis [45].
YWHAQ	A 14-3-3 protein, which mediates signal transduction by binding to phosphoserine-containing proteins; upregulated in ALS and peripheral nerve injury [185].

<u>*Down-regulated*</u>

Kinases –

ADCK3	A mitochondrial kinase; inhibiting its expression inhibits p53-induced apoptosis [47].
MYLK	(Myosin light chain kinase, also listed under Cytoskeletal DESR genes) An essential kinase for growth cone motility and cytokinesis; inhibiting MYLK inhibits axon retraction [73; 119].
CDK18	A cyclin-dependent kinase that also regulates cell migration and adhesion by negatively modulating FAK activity [194].
ROCK2	Kinase target of Rho-GTPase; it is involved in actin-mediated changes in the cytoskeleton. Inhibiting ROCK stimulates neurite outgrowth [139].
ERBB4	A member of the EGFR family of receptor tyrosine kinases, activated by neuregulins to induce a variety of cellular responses, including mitogenesis and differentiation, *e.g.*, see for review [85].
INSR	A receptor tyrosine kinase that regulates cellular functions in response to insulin; implicated in neuro-protection and synaptic plasticity [83].
MINK1	A MapKKK, STE family kinase-activator of MapK p38, JNK, and ERK [152].
CDK19	A cyclin dependent kinase belonging to a family required for transcriptional activation of specific genes, including the anti-inflammatory cytokine, IL-10 [121].

Phosphatases –

PTPRB	A receptor protein tyrosine phosphatase with a range of functions in cell adhesion, neurite growth and neuronal differentiation, *e.g.*, for review see [38].

Receptors –

DOCK10	A dopamine receptor interacting GEF essential for dendrite morphogenesis [118].
INSR	See kinases, above.
PTPRB	See phosphatases, above.

Ion Channels –

KCNN3	A small-conductance, calcium-activated potassium channel protein that contributes to neuronal after-hyperpolarization of the action potential; inhibiting its activity during diabetic ketoacidosis reduces brain inflammation [80].
KCNH5	(a.k.a., Ether-A-Go-Go-Related Gene Potassium Channel) An outward-rectifying, non-inactivating channel with a range of functions, including regulation of brain tumor growth, metastasis, and mitotic cell volume [111].
GRIK2	An ionotropic glutamate, Kainate type receptor linked with multiple neuropsychiatric and neurodevelopmental disorders [189].
CLCN2	A voltage-gated chloride channel associated with a range of disorders, including retinal degeneration, leukoencephalopathy, and epilepsy [310].
KCNJ2	A voltage-gated potassium channel essential for PDGF BB-stimulated vascular smooth muscle cell proliferation and migration [252].

Intracellular/Transmembranous Cell Signaling Pathway Mediators and Modulators –

PLCB4	A subunit of phospholipase C, which catalyzes IP3 and DAG production from PIP2; implicated in multiple processes important for axon regeneration and inhibiting it attenuates scar formation [131; 347].
TMTC1	An ER protein involved in maintaining calcium homeostasis [286].
MINK1	See kinases, above.

(Wnt-related)

CCDC136	An negative-regulator of Wnt/Beta-catenin signaling during zebrafish dorsal-ventral patterning [315].
DAAM2	A regulator of canonical Wnt signaling involved in stimulating re-myelination [155].
SHANK2	(See also, Cytoskeletal) An ankyrin-related protein implicated in a wide range of plasticity disorders, including synapse development through Wnt signaling [96; 273].

(G protein-related)

SYDE2	A rho GTPase homolog involved in p75 NTR receptor-mediated signaling, which stimulates cell death [312].
DOCK3	(See also Axon Outgrowth) A CNS-specific GEF that regulates axon outgrowth through activating Rac1 [220].

3 wk

<u>Up-regulated</u>

–*None* –

<u>Down-regulated</u>

–*None* –

<u>4. Intracellular Transport</u> – *functions in the intracellular trafficking of proteins and organelles*

Summary: Like those in the previous category, these genes were only found at the peak phase of regenerative axon outgrowth. Upregulated genes included five Golgi/endosome-related genes (*fabp7, atp6v1f, nipa1, rtn2, snx10*), seven nuclear transport-related genes (*nutf2, ranbp1, ppid, kpna2, crabp2, npm1, nup43*), and four genes associated with transport across the plasma membrane (*abcb1, slc38a4,*

nkain1, sfxn2). The six down-regulated DESR genes in this category included a regulator of ion transport (*fxyd1*), and two divalent metal cation transport mediators (*cnnm2, slc25a25*), among others.

3 days

Up-regulated

–*None*–

Down-regulated

–*None*–

1 wk/ 11 days

Up-regulated

Golgi/Endosome-related transport–

FABP7	(a.k.a. Brain Lipid Binding Protein) A transporter of fatty acids from extracellular to intracellular membranes; it is significantly upregulated in ependymal and radial glial cell endfeet during reactive gliosis in mammalian CNS injury, is stimulated by Notch signaling, and is important for radial glial cell development [6].
ATP6V1F	(See also Cellular Metabolism) A vesicular proton pump that acidifies vesicles for protein processing and sorting. It plays a functional role in eye development [227].
NIPA1	A magnesium transporter that associates with the early endosome and with cell membranes; mutations associated with upper motor neuron disease, *e.g.*, see for review [116].
RTN2	Essential for making tubular ER for vesicular transport; mutations cause axon degeneration [210].
SNX10	Involved in endosomal protein sorting and plays a critical role in matrix metalloproteinase secretion activated by stress [345].

Nuclear transport–

NUTF2	A transporter of proteins into the nucleus; it is upregulated in tail fin regeneration in zebrafish [267].
RANBP1	A small G protein involved in transporting proteins into the nucleus; its expression increases during axon regeneration [241].
PPID	A peptidylprolyl isomerase D activated by stress and essential for intracellular transport of activated glucocorticoid receptors into the nucleus [20].
KPNA2	Essential for nuclear import of key proteins; it is involved in regulation of TP53 activity [165].
CRABP2	Involved in nuclear uptake of RA-bound RAR; it is upregulated in limb regeneration in salamanders [195].
NPM1	A shuttling protein between the nucleolus and the nucleoplasm; it plays multiple roles in cells, including regulating TP53 responses, functioning as a histone chaperone, playing a role in ribosome synthesis, regulating centrosome duplication during the cell cycle, and destabilizing RNA-helices, *e.g.*, for review see [270].
NUP43	Regulates bidirectional transport across the nuclear membrane [323].

Mitochondrial transport–

FABP3	(a.k.a. Heart-type fatty acid binding protein) A protein that transports fatty acids from the cell membrane to mitochondria; it is released from cardiac myocytes after ischemic events and is a diagnostic marker for heart disease. Its increased

	expression has been linked to partially successful spinal cord regeneration in the neonatal opossum [225].
TOMM5	A mitochondrial transmembrane protein involved in transporting proteins into mitochondria [130].
SLC25A1	(See also Inflammatory Response and Wound Healing) Involved in transporting citrate across mitochondrial membranes. It regulates cellular metabolism and TP53 responses, and its expression is triggered by STAT. It is essential for NO and prostaglandin production in the inflammatory response [113].

Transmembrane transport–

ABCB1	An energy dependent efflux pump across intracellular membranes, implicated in a variety of age-related disorders [62].
SLC38A4	(See also General Metabolism) It functions as a neutral transmembrane amino acid transporter with a role in regulating protein synthesis in liver development [140].
NKAIN1	A regulator of sodium/potassium ATPase transporter; in Drosophila, mutants exhibit temperature-sensitive paralysis [82; 305].
SFXN2	A transmembrane transporter of tricarboxylic acids; little is known about SFXN2, but SFXN3 is implicated in regulating synaptic morphology [4].

Down-regulated

FXYD1	A regulator of ion transport; overexpression reduces neuronal dendritic arborization [26; 54].
ABCA2	(See also Lipid Metabolism) A gene involved in transporting lipids across membranes; it is highly expressed in brain and may play a role in macrophage lipid metabolism [51].
CNNM2	A divalent metal cation transport mediator thought to play a role in Mg^{++} homeostasis; down-regulation may play a neuroprotective role [167].
SLC25A25	A mitochondrial protein involved in ATP and divalent cation transport across mitochondrial membranes; it is important for maintaining metabolic efficiency [7].
SLC5A3	(a.k.a. Sodium/myo-inositol transporter) Important for regulating inositol mediated intracellular cell signaling and in regulating blood pressure [280].
SLC45A4	A sugar co-transporter [302].

3 wk

Up-regulated

–*None*–

Down-regulated

–*None*–

---

5. Post-Transcriptional Regulation – *functions in regulating RNA splicing, trafficking, translation, and decay*

Summary: Post-transcriptional control of gene expression is increasingly being seen as crucial for regenerative and developmental CNS axon outgrowth, as well as other forms of wound healing and regeneration. For example, cells under stress utilize cap-independent mRNA translation to ensure proteins needed for survival are synthesized, while cap-dependent mRNA translation decreases during the early phase of the stress response [148], and axonal cytoskeletal-related genes needed to rebuild the axon, such as the neurofilaments and tau, are under strong post-transcriptional control [175; 293]. During

neural development, selective translation of individual mRNAs requires specific ribosomal proteins [289]. Examples related to each of these three processes were: 1) *eif5b,* a translation initiation factor that promotes IRES-dependent mRNA translation [74], which was up-regulated at 3 days; 2) *aldoA,* an RNA-binding protein that stabilizes and increases translation of neurofilament mRNAs [28]), which was upregulated at 3 weeks; and 3) *rplp1,* a 60S ribosomal protein essential for brain development due to its effects on cyclin and p63 expression [248], which was down-regulated at 3 days in the two regenerative tissues and up-regulated in the non-regenerative one. Twenty-five additional DESR genes pointed to the importance of regulating RNA splicing (9 genes up-regulated at 7/11days), translation (five genes up-regulated at 7/11 days), mRNA trafficking and turnover (four genes up-regulated at 7/11 days and one up-regulated at 3 wk), and ribosomal composition (one genes down-regulated at 3 days; and four up-regulated at 7/11 days) for successful CNS axon regeneration. Particularly striking were examples from these 28 genes of ones that regulate specific transcripts and RNA-binding proteins already linked with regenerative axon outgrowth, neuronal survival, and other processes important for regeneration. They included the two up-regulated splicing factors *snrpd3* and *snrpn*, which have been implicated in Spinal Muscular Atrophy [69] and developmental axon outgrowth [337], respectively; *prmt1*, a methylase targeting hnRNP K [34], which is an RNA-binding protein essential for optic axon regeneration in *Xenopus* [175]; and *igf2bp3*, which binds and regulates trafficking of insulin-like growth factor mRNA in response to cytokine signaling [138] and *carhsp1*, which stabilizes TNFα [250].

3 days

<u>*Up-regulated*</u>

Translation Initiation

EIF5B	A eukaryotic translation initiation factor that helps position the ribosome on the mRNA for IRES-mediated, as opposed to cap-dependent, translation [74]. IRES-mediated translation initiation is important for cell survival during stress, when cap-dependent translation is suppressed [148].

<u>*Down-regulated*</u>

Ribosomal Subunits

RPLP1	An acidic 60S ribosomal protein with an important role in protein elongation during mRNA translation; while not essential for global protein synthesis, it is essential for embryonic brain development and cell proliferation due to its effects on the synthesis of key cell cycle and apoptosis regulators, including cyclins and p63 [248].
RPL37	A 60S ribosomal protein [176].

RNA Splicing

SRSF2	A splicing factor required for nuclear export and translation of mRNAs [63].

1 wk/ 11 days

<u>*Up-regulated*</u>

Splicing Factors

SNRPD3	A core component of the spliceosome; it forms a complex with Spinal Muscular Atrophy genes and is implicated in neurodegeneration [69].
SNRPN	A splicing co-factor implicated in axon outgrowth in zebrafish [337].
DDX39A	An ATP-dependent RNA helicase that associates with splicing speckles and with histone H2A.B [276].
SNRPF	A core component of the spliceosome, required for glioma cell migration [76].
LSM5	An Sm-like protein involved in nuclear mRNA splicing and mRNA decay; it facilitates the formation of the spliceosomal U4/U6 duplex [235].

U2AF2	Required for U2 binding to the branch site during pre-mRNA splicing; it is regulated by hnRNP A1 in alternative splicing [107].
PPIH	A peptidyl-prolyl Isomerase that is a component of the spliceosome; it may act as a chaperone for proteins joining the spliceosome [254].
UTP18	A gene involved in the processing of pre-18S ribosomal RNA and in RNA surveillance [292].

Ribosomal Subunits

RPL22L1	A ribosomal protein needed for translation of specific mRNAs. It can substitute for RPL22, which normally suppresses translation of RPL22L1, suggesting that ribosomal composition may have an impact on translation of specific mRNAs [228]. It plays a critical role in embryogenesis by working together with hnRNP A1 and in opposition to Rpl22 to regulate alternative splicing of smad2 pre-mRNA [228; 341].
RPL27A	Ribosomal protein L27A; it undergoes changes in gene expression in obesity [303].
RPS12	A 40S ribosomal protein thought to be involved in translation initiation. In Drosophila embryogenesis, cells expressing higher levels of RPS12 than their neighbors are more effectively eliminated in competition, giving it a specialized role in embryonic patterning [124].
MRPS17	A subunit of the mitochondrial 28S ribosome; in yeast, it promotes oxidative metabolism [94].

mRNA Translation Initiation and Elongation Factors

EEF1G	A translation elongation factor that participates in the delivery of aminoacylated tRNAs to the ribosome by anchoring it to other cell components. It binds specific mRNAs, such as vimentin, to escort them to their appropriate location in the cell for translation [46].
THOC7	Part of the TREX complex, involved in efficient translation and nuclear export of mRNAs with tandem polynucleotide repeats [64].
EEF1B2	A guanine nucleotide exchange factor needed to transfer aminoacylated tRNAs to the ribosome to promote peptide elongation; reduced expression is associated with cellular senescence [27].
DENR	A translation initiation factor that supports tissue growth by promoting re-initiation of translation downstream of uORFs [268].
EIF3I	An essential component of the apparatus needed to initiate mRNA translation; its overexpression enhances both cap-dependent and IRES-dependent translation of specific transcripts over and above that associated with general increases in translation [260].

Regulators of mRNA Trafficking and Turnover (mRNA binding proteins and their regulators)

IGF2BP3	An mRNA binding protein that regulates Insulin-like Growth Factor mRNA; it is required for cytokine signaling and is found in stress granules [138].
CARHSP1	An mRNA-binding protein that stabilizes TNF-α mRNA [250].
PRMT1	(See also Epigenetics) A protein arginine N-methyltransferase involved in methylation of certain hnRNP's and histones; *e.g.*, it methylates hnRNP K, an RNA-binding protein active in axon outgrowth [34].

<u>*Down-regulated*</u>

–None–

3 wk

Up-regulated

Regulators of mRNA Trafficking and Turnover (mRNA binding proteins and their regulators)

MEX3A	An RNA binding protein that regulates transcripts involved in tumorigenesis and aging in fish [12].
ALDOA	Although primarily known as a regulator of glycolysis, it is also an RNA-binding protein that binds to the 3'UTR of *nefl* mRNA. This binding stabilizes neurofilament mRNAs, enhancing their expression after axons reach their targets [28].

Down-regulated

–None–

---

6. Epigenetic gene regulation – ***functions in DNA-chromatin interactions, post-translational modifications to histones, DNA methylation/hydroxymethylation***

Summary: Genes associated with epigenetic gene regulation are increasingly being recognized as crucial for regeneration in multiple contexts [*e.g.*, [183]]. The 26 DESR genes in this category included specialized histone subclasses, chromatin proteins that associate with enzymatically modified histones and DNA, and the enzymes that make these modifications, as well as factors that regulate them. For example, at 3 days, two histone variants were down-regulated in the regenerative tissues: *hist2h2ab*, an H2 gene variant that interacts with the SWI/SNF complex to reposition nucleosomes for transcription [264]), and *hist1h4k*, an H4 gene variant whose induction is implicated in protecting cells from DNA damage by facilitating DNA double strand break repair [162]. At 7/11 days, there were sixteen up- and six down-regulated DESR genes. Two of the up-regulated genes were histone gene variants implicated in nucleosome repositioning: *h2afz*, an H2 gene variant [125; 253]] and *hist2h2ab*, which was down-regulated at 3 days. Three additional DESR genes associate with the SWI/SNF nucleosome repositioning complex: *smarca5*, which also interacts with the DNA methylase DNMT3B [77]), a*ctl6a*, an actin-like protein that antagonizes chromatin mediated transcriptional repression [335], and *smarca1* [232]. The first two were up-regulated and the last was down-regulated. Yet another gene has also been implicated in nucleosome repositioning, *hmgn2* [190]. Three DESR genes exhibited changes in expression that would be expected to promote histone deacetylation, which is increasingly recognized as crucial for axon regeneration [39], as well as regeneration of other tissues, such as cardiac muscle [277] and liver [110]. Two were up-regulated: a*np32a*, a histone acetyltransferase inhibitor [326], and *rbbp4,* a component of the Mi-2/NuRD complex [340] and one of several NuRD components required for successful fin regeneration in zebrafish [249]). A third was down-regulated: *gpt2* (Glutamic-pyruvic transaminase 2), an enzyme involved in pyruvate synthesis, which is needed for histone acetylation [104]. Two additional up-regulated DESR genes regulate acetyl CoA levels, which in turn metabolically regulate histone acetylation [313]: *acsbg2* and *acat2.* The remaining DESR genes from 7/11 days were associated with functions related to methylated histones, DNA or both. Related up-regulated genes were: *wdr77* (WD repeat domain 77), a histone methylase [297]); *uhrf2*, a protein required for 5-hydroxymethyl cytosine (5hmC) production [36]; *ahcy,* which metabolically regulates methyl group availability for DNA methylation [133]; *idh1*, which metabolically activates TET enzymes, which convert 5mC to 5hmC [299]; *cyb5a*, implicated in inducing global changes in CpG DNA methylation at promoters of inflammatory genes [186]; and *ezh2* (Enhancer Of Zeste 2 Polycomb Repressive Complex 2 subunit), a enzyme that regulates histone methylation and serves as a platform to recruit DNA methyltransferases [247]). Related down-regulated genes were: *jhdm1d* (KDM7A) a histone lysine demethylase [157]); *suz12*, involved in suppressing H3K9 and H3K27 methylation [271]; *apobec3a,* a cytidine deaminase that provides an alternative pathway for demethylating DNA [29]); and *jarid2,* a transcriptional repressor that recruits the Polycomb Repressive Complex 2 to chromatin to promote methylation of H3K9 and H3K27 [163; 245]. Notably, *jarid2* was the only one of these genes that persisted as a DESR gene at 3 weeks, when it continued to be down-regulated.

Appendix: Genes Implicated by Expression Analysis to be Involved in Successful CNS Axon Regeneration: Cellular Functions, Time of Differential Expression, and Key References

3 days

Up-regulated

–*None*–

Down-regulated

HIST2H2AB*	A histone H2 variant that interacts with the SWI/SNF chromatin remodeling complex, which repositions histones to allow transcription [264] (Note, this gene was up-regulated at 1 wk/ 11 days).
HIST1H4K	A subtype of histone H4 that has been implicated in protecting cells from DNA damage and in facilitating DNA double strand break repair [162].

1 wk/ 11 days

Up-regulated

Genes Involved in Nucleosome Repositioning during Transcription

(Histone Variants)

HIST2H2AB*	See 3 days, when this gene was down-regulated in successful regeneration; it was upregulated at this peak phase of regenerative axonal outgrowth.
H2AFZ	A specialized Histone 2 variant (a.k.a., H2A.Z) required for embryonic development in mammals; it marks the 5' ends of genes and is involved in nucleosome repositioning during transcription, contributing to Pol II pausing behavior, which potentially allows for more interactions with transcription factors and epigenetic modifiers at specific sites [37; 65; 125; 253].

(Other genes involved in nucleosome repositioning)

SMARCA5	A component of the SWI/SNF complex involved in repositioning histones to allow transcription; it is a helicase that promotes the open complex. It also interacts with DNMT3B and is therefore associated with changes in DNA methylation [77].
ACTL6A	A component of the BAF nucleosome remodeling complex, which antagonizes chromatin mediated transcriptional repression; implicated in learning and long-term memory consolidation [335].
HMGN2	(See also Transcription Factors) A transcription co-factor implicated in maintaining DNA in the open conformation for transcription by binding to and modulating histone H3 and removing H1 from promoters [190].

Regulators of Histone Acetylation /De-acetylation

ANP32A	An enzyme involved in the inhibition of histone acetyltransferases (up-regulation de-acetylates histones) [31; 255; 326].
RBBP4	A chromatin remodeling factor present in Mi-2/NuRD complexes involved in histone deacetylation, DNA methylation, and gene repression; it is required for successful fin regeneration in zebrafish [249; 340].
ACSBG2	An acyl-CoA synthetase, which helps to metabolically regulate histone acetylation through the availability of acyl groups to histone acetyltransferases [246; 313].
ACAT2	An enzyme (acetyl coA transferase) involved in pyruvate synthesis, which helps regulate levels of acyl-acetate in the cell. Such enzymes are metabolic regulators of the enzymes that acetylate histones by making the required substrates available [52; 313].

Appendix: Genes Implicated by Expression Analysis to be Involved in Successful CNS Axon Regeneration:
Cellular Functions, Time of Differential Expression, and Key References

Regulators of Histone and DNA Methylation (Hydroxy-methylation)/De-Methylation

EZH2	An enzyme that maintains and/or increases histone methylation levels, especially at H3K27. As a member of the Polycomb Repressive Complex; it also serves as a platform to recruit DNA methyltransferases to DNA and is functionally important for a range of developmental and physiological changes in gene expression, including responses to injury [247].
IDH1	A cytosolic isocitrate dehydrogenase: that metabolically regulates TET enzymes, which convert 5mC to 5hmC (hydroxy-methylation), which is needed for subsequent steps in the pathway that converts 5 mC to de-methylated C [299].
UHRF2	An E3 ubiquitin ligase that not only regulates nuclear protein ubiquitination but also binds methylated DNA and is required for the production of 5hmC [36].
AHCY	An enzyme that metabolically regulates the availability of methyl groups needed for DNA (and possibly histone) methylation [133].
WDR77	A chromatin remodeling enzyme that converts histone arginines to dimethylarginines [297].
CYB5A	An enzyme that regulates stearyl-CoA-desaturase activity, which in turn induces global changes in CpG DNA methylation at promoters to, for example, regulate inflammatory gene expression 3T3 adipocytes [186; 200].
PRMT1	(See also Post-transcriptional Regulation) A protein arginine N-methyltransferase involved in methylation of histones and certain hnRNP's; *e.g.*, it methylates hnRNP K, an RNA-binding protein active in axon outgrowth [34].
CMPK1	(See also DNA Replication/Repair) A cytidine/uridine monophosphate kinase that is required for nucleic acid biosynthesis; it converts CMP, UMP and dCMP into the di-phosphate forms. It also plays a role in determining the efficiency of DNA repair involving cytidine/uridine, and therefore may play a role in Base Excision Repair pathways involved in altering DNA methylation [296].

<u>*Down-regulated*</u>

Genes Involved in Nucleosome Repositioning during Transcription

SMARCA1	Part of the SWI/SNF nucleosome remodeling complex involved in repositioning histones for global gene regulation [232].

Regulators of Histone Acetylation /De-acetylation

GPT2	An enzyme involved in pyruvate synthesis, which metabolically regulates histone acetylation [104].

Regulators of Histone and DNA Methylation (Hydroxymethylation)/De-Methylation

JHDM1D	A histone lysine demethylase that specifically demethylates H3K9me2, H3K27me2, and H4K20me1, and also binds H3K4me3 to demethylate H3K27me2; it plays an important role in neural differentiation [108].
SUZ12	A Polycomb Repressive Complex 2 subunit involved in suppressing the methylation of H3K9 and H3K27 and also serves as a recruitment platform for DNA methyltransferases; it is downregulated in successful wound healing of murine skin [271].
JARID2*	A transcriptional repressor that recruits the Polycomb Repressive Complex to genes to promotes methylation of H3K9 and H3K27, *e.g.*, see [163; 245].
GPT2	An enzyme involved in pyruvate synthesis, which metabolically regulates histone acetylation [104].

APOBEC3A A cytidine deaminase, which converts cytidines in DNA and RNA to uridines. In cell lines, it can de-methylate 5mC (5-methyl-cytosine) in DNA. Up-regulation of this enzyme also promotes DNA double strand breaks and cell death [29; 216].

3 wk

Up-regulated

–None–

Down-regulated

JARID2* See 1 wk/11 days

---

7. Axon Outgrowth – *functions in supporting neurite/axon outgrowth and axon guidance*

Summary: This category included fifteen genes previously implicated in promoting and inhibiting axon outgrowth and in axon guidance, all of which were differentially expressed at the peak phase of regenerative axon outgrowth. The ten up-regulated genes included *lypla2*, which activates GAP-43 [141], *dpysl3*, *dbcbld2*, and *crmp1*, which are associated with sempahorin/neuropilin mediated axon guidance [21; 196; 224], and *st8sia4*, the principal enzyme mediating polysialation of NCAM [236], among others. The down-regulated DESR genes included two oligodendrocytic myelin components (*plp1*, *mbp*), two protocadherins (*pcdh1, pcdh10*), and a GEF that regulates axon outgrowth through Rac1, *dock3* [219; 220].

3 days

Up-regulated

–None–

Down-regulated

–None–

1 wk/ 11 days

Up-regulated

Genes that Regulate Molecules Involved in Axon Guidance and Outgrowth–

LYPLA2 A Lysophospholipase that hydrolyzes fatty acids from S-acylated cysteine residues on GAP43 to activate it [141].

FSCN1 An actin bundling protein that interacts with MAPK and has functions in neurite outgrowth and cell migration [144; 342].

DPYSL3 A dihydropyraminidase that plays a role in semaphorin 3-mediated axon guidance [196].

GDAP1L1 Plays a role in ganglioside-induced neuronal differentiation and neurite outgrowth. Mutations in this gene are associated with peripheral axonopathy, Charcot-Marie Tooth disease [171; 188].

DCBLD2 A neuropilin-like protein (neuropilins are axon guidance receptors for semaphorins); it is also involved in promoting angiogenesis in zebrafish [224].

ODC1 (see also Cellular Metabolism) An ornithine decarboxylase that is required for regenerative outgrowth of frog sciatic axons [61].

B3GNT2 A transmembrane protein involved in regulating extracellular, poly-N-acetyl-lactosamine chains on guidance adhesion molecules [101].

CRMP1 A nervous system-specific protein that is part of the semaphorin signal transduction pathways implicated in semaphorin-induced growth cone collapse active in axon guidance [21].

PVRL1 A cell adhesion molecule with roles in cell motility and axon guidance; for review see [187].

ST8SIA4 — The principal enzyme that adds polysialic acid (PSA) to cell adhesion molecules, such as N-CAM; increasing PSA-NCAM promotes functional recovery after SCI in mice [236].

<u>*Down-regulated*</u>

Myelin components –

PLP1 — A major component of oligodendrocytic myelin; PLP1 knockout in mice leads to CNS axon degeneration [179].

MBP — A major component of myelin both in the CNS and the PNS. MBP influences neurite outgrowth, neuronal cell migration and survival, and myelination [180].

Cell Adhesion Molecules –

PCDH1 — A protocadherin cell adhesion molecule involved in neural development and neurite outgrowth [98].

PCDH10 — A second protocadherin implicated in regulating neuronal connectivity; it also inhibits PI3K/akt signaling in liver cancer [284; 328].

Small G-protein Regulators –

DOCK3 — (See also Cell Signaling) A CNS-specific GEF that regulates axon outgrowth through activating Rac1 [220].

3 wk

<u>*Up-regulated*</u>

–None–

<u>*Down-regulated*</u>

–None–

<u>*8. DNA Replication/Repair*</u> – *functions in mitosis, DNA repair and mitotic checkpoints*

Summary: Of the seventeen genes, five have been associated with regulating mitotic checkpoints: one down-regulated gene at 3 days (*hp1bp3*), and four up-regulated genes at 7/11 days (*spc25, ccnb3, mad2l1, rprm*). Such genes are increasingly understood to be important to allow cells time to rearrange their chromosomes in preparation for major changes in gene expression [217]. The remaining twelve were up-regulated genes at 7/11 days that are more directly involved in DNA replication and repair (*mcm6, pcna, top2a, erh, ncaph2, rfc5, sycp2, smc2, kiaa0101, mcm7, cmpk1* (see also Epigenetic)*, and rrm2*).

3 days

<u>*Up-regulated*</u>

–None–

<u>*Down-regulated*</u>

Mitotic Checkpoint Protein–

HP1BP3 — A heterochromatin protein with structural similarity to H1, suggesting it binds DNA in the linker region between nucleosomes. It principally acts as a mitotic checkpoint protein, maintaining heterochromatin integrity during the G1/S phase; it also influences gene transcription and increases cell viability during hypoxia [58; 59].

Appendix: Genes Implicated by Expression Analysis to be Involved in Successful CNS Axon Regeneration:
Cellular Functions, Time of Differential Expression, and Key References

1 wk/ 11 days

Up-regulated

Mitotic Checkpoint Proteins –

SPC25	A component of the kinetochore complex, with mitotic checkpoint activity; it is upregulated when cancer stem cell proliferation is activated [17; 242].
CCNB3	A cyclin-related positive regulator of cell division kinases and mitotic checkpoints; it is necessary for the progression of cells out of M phase. It plays a critical role in the expression of the survival signal survivin [30].
MAD2L1	A mitotic checkpoint protein that prevents cells from passing from anaphase to telophase until all chromosomes are properly aligned; it is also potentially involved in DNA repair and ensuring genome stability by slowing down or halting mitosis until repair takes place [153].
RPRM	A TP53-dependent mitotic checkpoint protein that arrests the cell cycle at G2 [109; 291].

Genes Involved Directly in DNA Replication and Repair –

MCM6	An essential gene for the initiation of genome replication; is implicated in stimulating Müller glia cell division during limited retinal regeneration in mice [177; 223].
PCNA	The sliding clamp protein of DNA polymerase; it is predominantly used as a marker for proliferating cells and is found in microglia and astrocytes after spinal cord injury in rat [316].
TOP2A	A DNA topoisomerase essential for both DNA replication and DNA transcription; in zebrafish, it is required for both embryonic development and liver regeneration [56].
ERH	It is essential for chromosome alignment at metaphase and plays a role both in regulating the cell cycle and in the DNA damage response [70].
NCAPH2	It plays an essential role in mitotic chromosome assembly and is involved in chromatin architectural rearrangements associated with cellular senescence [105; 332].
RFC5	It is part of the PCNA sliding clamp loader and is required for DNA replication [182; 212].
SYCP2	An essential component for synapsis of sister homologous chromosomes in meiosis, with few known functions outside of meiosis; expression in cells not in meiosis may reflect changes in genome architecture [192].
SMC2	An essential component of the condensin complex, which condenses chromatin for mitosis; it also plays an important role in DNA repair [321].
KIAA0101	A PCNA-clamp associated factor, also involved in DNA repair (see PCNA for references).
MCM7	A DNA helicase that belongs to the same complex as MCM6; it is essential for the initiation of genome replication [158; 223].
CMPK1	(See also Epigenetic Gene Regulation) A cytidine/uridine monophosphate kinase that is required for nucleic acid biosynthesis; it converts CMP, UMP and dCMP into the di-phosphate forms. It also plays a role in determining the efficiency of DNA repair involving cytidine/uridine, and therefore may play a role in base excision repair pathways involved in altering DNA methylation [296].
RRM2	An enzyme that converts ribonucleotides to deoxyribonucleotides. It becomes more active during mitosis and is a rate limiting enzyme for both DNA replication and DNA repair; it is upregulated in liver regeneration [71; 178].

<u>Down-regulated</u>

–*None*–

3 wk

<u>Up-regulated</u>

–*None*–

<u>Down-regulated</u>

–*None*–

<u>9. Lipid Metabolism</u> – *functions in fatty acid and lipid biosynthesis and degradation*

Summary: These fifteen DESR genes have been implicated in processes related to lipid metabolism; all were differentially expressed at 7/11 days. Up-regulated genes had functions in cholesterol biosynthesis (*cyb5r2, nsdhl, mvk, c140rf1, dhcr7, hmgcr*), as well as the synthesis and modification of other lipids (*fads1, fads2, ecl2, gla, lppr3*). One additional up-regulated gene in this category was *apoE,* which is the major lipoprotein carrier protein in the brain and has been linked to Alzheimer's disease, as well as mammalian peripheral axon regeneration [44; 298]. All three down-regulated genes had functions in the metabolism of lipids in glia, especially sphingolipid biosynthesis (*ugt8, ppt2, abca2*).

3 days

<u>Up-regulated</u>

–*None*–

<u>Down-regulated</u>

–*None*–

1 wk/ 11 days

<u>Up-regulated</u>

Cholesterol Biosynthesis–

CYB5R2	A cytochrome B5 reductase implicated in cholesterol biosynthesis, fatty acid desaturation and elongation; it is associated with respiratory bursts in neutrophils [19].
NSDHL	An NAD(P)-dependent steroid dehydrogenase involved in cholesterol biosynthesis. In the developing CNS, NSDHL knockout in radial glia leads to loss of cortical, hippocampal, and cerebellar granule cells as a result of defective Sonic Hedgehog Signaling [48].
MVK	A key early enzyme in sterol and isoprenoid synthesis, thought to be a principal regulator of cholesterol biosynthesis. Deficiencies lead to severe auto-inflammation in the nervous system and other tissues [106; 295].
C14orf1	(a.k.a. Ergosterol Biosynthetic Protein 28 Homolog) An important enzyme in the biosynthesis of cholesterol and other sterols [72; 300].
DHCR7	A required enzyme for cholesterol biosynthesis. Disrupting cholesterol biosynthesis leads to neurodegeneration [81].
HMGCR	A rate limiting enzyme in cholesterol synthesis and a therapeutic target of statins; inhibiting this enzyme inhibits neurodegeneration [263].

Metabolism of other Lipids–

FADS1	(See also Inflammatory Response and Wound Healing) A fatty acid desaturase that is directly implicated in down-regulating inflammation [92].

FADS2	A second fatty acid desaturase; increased expression is a marker for the anti-inflammatory state [92].
ECI2	An enzyme that catalyzes the conversion of cis- or trans- double bonds of fatty acids at gamma-carbon positions to trans double bonds at beta-carbon positions. It is important for the metabolism of certain fatty acids for energy in mitochondria [117].
GLA	An important metabolic enzyme that hydrolyzes the terminal alpha-galactosyl moieties from glycolipids, and also glycoproteins. Mutations in GLA cause Fabry disease, a lysosomal storage disease involving dysfunctional metabolism of sphingolipids, which are abundant in the plasma membranes of neurons and glia [43].
LPPR3	A phospholipid phosphatase active during cell migration, neurite retraction, and mitogenesis [337].

Lipid Carrier Lipoprotein–

APOE	The principal lipoprotein carrier in the brain. Mutations in APOE are strongly linked to Alzheimer's disease in humans. Isoforms elicit varying effects on peripheral nerve regeneration in mammals [44; 298].

Down-regulated

Glial Lipid Metabolism –

UGT8	(See also Inflammatory Response and Wound Healing) An important enzyme for sphingolipid metabolism, which are important for making myelin; elevated UGT8 promotes neuroinflammation in humans [339].
PPT2	(See also Inflammatory Response and Wound Healing) A critical enzyme in the synthesis of sphingosine, which is especially important for the synthesis of sphingolipids in myelin. Elevated sphingolipid levels promote neuro-inflammation, and PPT2 knockdown in mice leads to neurodegeneration [89; 281; 339].
ABCA2	(See also Intracellular Transport) A gene involved in transporting lipids across membranes; it is highly expressed in brain and may play a role in macrophage lipid metabolism [51].

3 wk

Up-regulated

–*None*–

Down-regulated

–*None*–

---

10. Transcription Factors – *functions in regulating gene transcription by binding to DNA*

Summary: Transcription factors were found among DESR genes at all three time points. At the earliest time point, only a single transcription factor, *ddit3*, was up-regulated. *Ddit3* is a C/EBP-related transcriptional repressor involved in the activation of pro-inflammatory signals [239]. At 7/11 days, there were eight up-regulated and three down-regulated transcription factors and co-factors. Among the up-regulated DESR transcription factors at this time were two homeodomain-related genes [a LIM homeodomain protein (*fhl3*) and a triple-homeobox factor (*tgif1*) previously shown to be required for retinal regeneration in zebrafish [159]], two bHLH transcription factors [*hes5*, which is activated by Notch signaling after spinal cord injury in mammals [126], and *mycl1*, a proto-oncogene], a transcriptional co-factor involved in nucleosome repositioning (*hmgn2*), a transcription co-factor involved in activating STAT3 (*mllt11*), a Scratch/Snail transcriptional repressor (*scrt2*), and the SRY-box family member (*sox11*), which is already known to play a critical role in both spinal cord and optic nerve injury in mammal [226; 314]. The three down-regulated DESR transcription factor/co-factor genes were *znf395* (a zinc-

finger transcription factor that activates pro-inflammatory cytokines [102]), *bcl6* (a transcriptional repressor of STAT3 [214]), and *prr12*. Only two transcription factors were among DESR genes at 3 weeks (*ebf3* and *irf8*). Both were up-regulated and have roles in regulating genes involved in apoptosis and in suppressing a hyper-immune response in macrophages, respectively [115; 164].

3 days

Up-regulated

DDIT3	(See also Inflammatory Response and Wound Healing) A C/EBP-related transcription factor that functions primarily as a transcriptional repressor by forming inactive partners with other C/EBP transcription factors; it is regulated by stress factors and is important for the activation of pro-inflammatory signals [239].

Down-regulated

–*None*–

1 wk/ 11 days

Up-regulated

FHL3	A LIM domain, double-zinc-finger-motif transcriptional co-activator/co-repressor. It interacts with SMAD2, SMAD3, and SMAD4, MyoD, and the high-affinity IgE beta chain regulator MZF-1 [93].
HES5 (3)	A HES family bHLH transcription repressor of bHLH genes; it is activated by notch/delta signaling, and stimulation of Hes5 has been shown to promote spinal cord injury repair [126].
HMGN2	(See also Epigenetic Gene Regulation) A transcription co-factor implicated in maintaining DNA in the open conformation for transcription by binding to and modulating histone H3 and removing H1 from promoters [190].
MYCL1	The L-Myc proto-oncogene; a bHLH transcription factor implicated in cellular trans-differentiation, *e.g.*, fibroblasts into myoblasts [304].
MLLT11	A transcription co-factor that increases activation of STAT3, a transcription factor that is important for axon regeneration; exogenous expression in cell lines (HEK) also induces expression of TuJ1, a neuron-specific tubulin [166; 238].
SCRT2	A Scratch/Snail family transcriptional repressor that regulates neuronal differentiation and subsequent neuronal cellular migration in opposition to the bHLH transcription factors, Ngn/NeuroD1 [243].
SOX11	A member of the family of SRY-box family of transcription factors. SOX11 plays an important role in neural development, and overexpression in mouse SCI promotes corticospinal tract regeneration but interferes with functional recovery. In ONC, it promotes axon regeneration of a subset of RGCs, but kills others in mice [226; 314].
TGIF1	A transcription factor of the triple homeobox family; it inhibits RAREs and SMAD2. Its expression is required for regeneration of retina in zebrafish from Müller cells [159].

Down-regulated

ZNF395	(a.k.a Huntington Disease Regulatory Region Binding Protein 2; see also Inflammatory Response and Wound Healing): A zinc-finger transcription factor that activates pro-inflammatory cytokines [102].
BCL6	A transcriptional repressor of STAT3 [213].
PRR12	A DNA binding protein with a role in neurodevelopment [154].

3 wk

Up-regulated

EBF3	(See also Cytoskeletal) A transcription factor that regulates cell survival by regulating genes involved in cell cycle arrest and apoptosis. It inhibits glial cell proliferation and glioma-genesis and also binds directly to microtubules to induce bundling [25; 164].
IRF8	(See also Inflammatory Response and Wound Healing) A transcription factor that responds to interferons to suppress the hyper-inflammatory response in macrophages [115].

Down-regulated

–None–

---

11. Cellular Metabolism – *functions in regulating general aspects of cellular metabolism*

Summary: This group of DESR genes has known roles in regulating cellular metabolism. Eight were up-regulated, and one (*fndc7*) was down-regulated, all at 7/11 days. Up-regulated genes included an ornithine decarboxylase (*odc1*) required for peripheral axon regeneration [61], and *c2orf47*, which is a mitochondrial protein implicated in protecting cells from mitochondrial dysregulation in spinocerebellar ataxia [142].

3 days

Up-regulated

–None–

Down-regulated

–None–

1 wk/ 11 days

Up-regulated

SLC38A4	(See also Intracellular Transport) It functions as a neutral transmembrane amino acid transporter with a role in regulating protein synthesis in liver development [140].
ATP5E	An ATP synthase that is used as a mitochondrial marker for increased cellular metabolism; it co-localizes with galectin-3 and has been implicated in inflammation [97; 134; 229].
ATP6V1F	(see also Intracellular Transport) A vesicular proton pump that acidifies vesicles for protein processing and sorting. It plays a functional role in eye development [227].
ODC1	(See also Axon Outgrowth) Ornithine decarboxylase, which catalyzes conversion of ornithine to putrescine; it is required for regeneration of frog sciatic axons [61].
C2orf47	An uncharacterized mitochondrial protein; it protects cells from mitochondrial dysregulation in spinocerebellar ataxia [142].
C21orf33	(a.k.a. mitochondrial ES1 homolog, KPNI, and HES1) A member of the multi-functional DJ-1/Pfpl gene family found in mitochondria, where it regulates energy metabolism [193].
GPD1L	A glycerol phosphate dehydrogenase, which is a family of enzymes that catalyze conversion of dihydroxyacetone phosphate to glycerol 3-phosphate, forming an important link between carbohydrate and lipid metabolism. It is a genetic cause of Brugada syndrome [184].
GNPDA2	An enzyme that converts D-glucosamine-6-phosphate into D-fructose-6-phosphate and ammonium. It is a metabolic enzyme associated with regulating body weight and obesity [319].

Appendix: Genes Implicated by Expression Analysis to be Involved in Successful CNS Axon Regeneration: Cellular Functions, Time of Differential Expression, and Key References

<u>*Down-regulated*</u>

FNDC7	A fibronectin-domain-containing protein whose precise function is unknown, but a related gene (FNDC5) promotes energy expenditure by stimulating production of brown adipose tissue to attenuate inflammation [322]. It may therefore have some function in regulating cellular metabolism.

3 wk

<u>*Up-regulated*</u>

–None–

<u>*Down-regulated*</u>

–None–

**The same gene is listed at another time point in this functional category.*

[1]These genes' expression increased significantly (FDR < 0.05) in both regenerative tissues after injury (tadpole SCI hindbrain & frog ONC eye), but not in the non-regenerative frog SCI hindbrain.

[2]These genes' expression decreased significantly (FDR < 0.05) in both regenerative tissues after injury (tadpole SCI hindbrain & frog ONC eye), but not in the non-regenerative frog SCI hindbrain.

[3]Genes in Black: In using an FDR (Q) < 0.05 as the criterion for statistical significance, these genes changed significantly in expression (either up or down as indicated) in successful CNS regeneration, but not in unsuccessful regeneration.

[4]Genes in Green: These genes were significantly up-regulated in successful CNS regeneration and down-regulated with unsuccessful CNS regeneration. They are therefore likely to be particularly pro-regenerative.

[5]Genes in Red: These genes were significantly down-regulated in successful CNS regeneration and up-regulated with unsuccessful CNS regeneration. They are therefore likely to be particularly anti-regenerative.

References

1. Acs P, Bauer PO, Mayer B, Bera T, Macallister R, Mezey E, Pastan I. A novel form of ciliopathy underlies hyperphagia and obesity in Ankrd26 knockout mice. Brain Struct Funct. 2015;220:1511-28.
2. Altinoz MA, Ozcan EM, Ince B, Guloksuz S. Hemoglobins as new players in multiple sclerosis: metabolic and immune aspects. Metab Brain Dis. 2016;31:983-92.
3. Alvarez-Fernandez C, Tamirisa S, Prada F, Chernomoretz A, Podhajcer O, Blanco E, Martin-Blanco E. Identification and functional analysis of healing regulators in *Drosophila*. PLoS Genet. 2015;11:e1004965.
4. Amorim IS, Graham LC, Carter RN, Morton NM, Hammachi F, Kunath T, Pennetta G, Carpanini SM, Manson JC, Lamont DJ, Wishart TM, Gillingwater TH. Sideroflexin 3 is an alpha-synuclein-dependent mitochondrial protein that regulates synaptic morphology. J Cell Sci. 2017;130:325-31.
5. An HJ, Shin H, Jo SG, Kim YJ, Lee JO, Paik SG, Lee H. The survival effect of mitochondrial Higd-1a isassociated with suppression of cytochrome c release and prevention of caspase activation. Biochim Biophys Acta. 2011;1813:2088-98.
6. Anthony TE, Mason HA, Gridley T, Fishell G, Heintz N. Brain lipid-binding protein is a direct target of Notch signaling in radial glial cells. Genes Dev. 2005;19:1028-33.

7. Anunciado-Koza RP, Zhang J, Ukropec J, Bajpeyi S, Koza RA, Rogers RC, Cefalu WT, Mynatt RL, Kozak LP. Inactivation of the mitochondrial carrier SLC25A25 (ATP-Mg2+/Pi transporter) reduces physical endurance and metabolic efficiency in mice. J Biol Chem. 2011;286:11659-71.
8. Aoki K, Shimoada K, Oritani K, Matsuda Teal. Limitin, an interferon-like cytokine, transduces inhibitory signals on B-cell growth trhough activation of Tyk2, but not Stat1, followed by induction and nuclear translocation of Daxx. Exp Hematol. 2003;31:1317-22.
9. Aramaki T, Sasai N, Yakura R, Sasai Y. Jiraiya attenuates BMP signaling by interfering with type II BMP receptors in neuroectodermal patterning. Dev Cell. 2010;19:547-61.
10. Atkin G, Hunt J, Minakawa E, Sharkey L, Tipper N, Tennant W, Paulson HL. F-box only protein 2 (Fbxo2) regulates amyloid precursor protein levels and processing. J Biol Chem. 2014;289:7039-48.
11. Avraham Y, Davidi N, Lassri V, Vorobiev L, Kabesa M, Dayan M, Chernoguz D, Berry E, Leker RR. Leptin induces neuroprotection, neurogenesis, and angiogenesis after stroke. Curr Neurovasc Res. 2011;8:313-22.
12. Baumgart M, Groth M, Priebe S, Savino A, Testa G, Dix A, Ripa R, Spallotta F, Gaetano C, Ori M, Terzibasi Tozzini E, Guthke R, Platzer M, Cellerino A. RNA-seq of the aging brain in the short-lived fish *N. furzeri* - conserved pathways and novel genes associated with neurogenesis. Aging Cell. 2014;13:965-74.
13. Bayat B, Tjahjono Y, Berghöfer H, Werth S, Deckmyn H, De Meyer SF, Sachs UJ, Santoso S. Choline transporter-like protein-2: new von Willebrand factor-binding partner involved in antibody-mediated neutrophil activation and transfusion-related acute lung injury. Arterioscler Thromb Vasc Biol. 2015;35:1616-22.
14. Beniash E. Co-option of hair follicle keratins into amelogenesis is associated with the evolution of prismatic enamel: a hypothesis. Front Physiol. 2017;8:823.
15. Benowitz LI, He Z, Goldberg JL. Reaching the brain: advances in optic nerve regeneration. Exp Neurol. 2017;287 (Pt 3):365-73.
16. Benowitz LI, Popovich PG. Inflammation and axon regeneration. Curr Opin Neurol. 2011;24:577-83.
17. Bharadwaj R, Qi W, Yu H. Identification of two novel components of the human NDC80 kinetochore complex. J Biol Chem. 2004;279:13076-85.
18. Bhat R, Eissmann P, Endt J, Hoffman S, Watzl C. Fine-tuning of immune responses by SLAM-related receptors. J Leukoc Biol. 2006;79:417-24.
19. Borregaard N, Tauber AI. Subcellular localization of the human neutrophil NADPH oxidase b-cytochrome and associated flavoprotein. J Biol Chem. 1984;259:47-52.
20. Bourke CH, Raees MQ, Malviya S, Bradburn CA, Binder EB, Neigh GN. Blucocorticoid sensitizers Bag1 and Ppid are regulated by adolescent stress in a sex-dependent manner. Psychoneuroendocrinology. 2013;38:84-93.
21. Bretin S, Reibel S, Charrier E, Maus-Moatti M, Auvergnon N, Thevenoux A, Glowinski J, Rogemond V, Premont J, Honnorat J, Gauchy C. Differential expression of CRMP1, CRMP2A, CRMP2B, and CRMP5 in axons or dendrites of distinct neurons in the mouse brain. J Comp Neurol. 2005;486:1-17.
22. Brinchmann MF, Patel DM, Iversen MH. The role of galectins as modulators of metabolism and inflammation. Mediators Inflamm. 2018;2018:9186940.
23. Brown CO, Schibler J, Fitzgerald MP, Singh N, Salem K, Zhan F, Goel A. Scavenger receptor class A member 3 (SCARA3) in disease progresson and therapy resistance in multiple myeloma. Leuk Res. 2013;37:963-9.
24. Brown J, Bridgman PC. Role of myosin II in axon outgrowth. J Histochem Cytochem. 2003;51:421-8.
25. Bu W, Su LK. Regulation of microtubule assembly by human EB1 family proteins. Oncogene. 2001;20:3185-92.
26. Busch R, Qiu W, Lasky-Su J, Morrow J, Criner G, DeMeo D. Differential DNA methylation marks and gene comethylation of COPD in African-Americans with COPD exacerbations. Respir Res. 2016;17:143.

27. Byun HO, Han NK, Lee HJ, Kim KB, Ko YG, Yoon G, Lee YS, Hong SI, Lee JS. Cathepsin D and eukaryotic translation elongation factor 1 as promising markers of cellular senescence. Cancer Res. 2009;69:4638-7.
28. Cañete-Soler R, Reddy KS, Tolan DR, Zhai J. Aldolases A and C are ribonucleolytic components of a neuronal complex that regulates the stability of the light neurofilament mRNA. J Neurosci. 2005;25:4353-64.
29. Carpenter MA, Li M, Rathore A, Lackey L, Law EK, Land AM, Leonard B, Shandiva SM, Bohn MF, Schiffer CA, Brown WL, Harris RS. Methylcytosine and normal cytosine deamination by the foreign DNA restriction enzyme APOBEC3A. J Biol Chem. 2012;287:34801-8.
30. Castedo M, Perfettini JL, Rournier T, andreau K, Medema R, Kroemer G. Cell death by mitotic catastrophe: a molecular definition. Oncogene. 2004;23:2825-37.
31. Chai GS, Feng Q, Wang ZH, Hu Y, Sun DS, Li XG, Ke D, Li HL, Liu GP, Wang JZ. Downregulating ANP32A rescues synapse and memory loss via chromatin remodeling in Alzheimer model. Mol Neurodegener. 2017;12:34.
32. Chakrabarti R, Celia-Terrassa T, Kumar S, Hang X, Wei Y, Choudhury A, Hwang J, Peng J, Nixon B, Grady JJ, DeCoste C, Gao J, Van Es JH, Li MO, Aifantis I, Clevers H, Kang Y. Notch ligand Dll1 mediates cross-talk between mammary stem cells and the macrophage niche. Science. 2018;360:pii: eaan4153.
33. Chamoto K, Gibney BC, Lee GS, Ackermann M, Konerding MA, Tsuda A, Mentzer SJ. Migration of CD11b+ accessory cells during during lung regeneration. Stem Cell Res. 2013;10:267-77.
34. Chan JY, Hsieh TY, Liu ST, Chou WY, Chung MH, Huang SM. Physical and functional interactions between hnRNP K and PRMT family proteins. FEBS Lett. 2009;583:281-6.
35. Cheever TR, Olson EA, Ervasti JM. Axonal regeneration and neuronal function are preserved in motor neurons lacking β-actin in vivo. PLoS ONE. 2011;6:e17768.
36. Chen R, Zhang Q, Duan X, York P, Chen GD, Yin P, Zhu H, Xu M, Chen P, Wu Q, Li D, Samarut J, Xu G, Zhang Q, Duan X, Li J, Wong J. The 5-hydroxymethylcytosine (5hmC) reader UHRF2 is required for normal levels of 5hmC in mouse adult brain and spatial learning and memory. J Biol Chem. 2017;292:4533-43.
37. Chen Z, Gabizon R, Brown AI, Lee A, Song A, Diaz-Celis C, Kaplan CD, Koslover EF, Bustamante C. High-resolution and high-accuracy topographic and transcriptional map of the nucleosome barrier. eLife. 2019;8:pii.e48281.
38. Chicote JU, DeSalle R, Garcia-España A. Phosphotyrosine phosphatase R3 receptors: origin, evolution and structural diversification. PLoS ONE. 2017;12:e0172887.
39. Cho Y, Cavalli V. HDAC signaling in neuronal development and axon regeneration. Curr Opin Neurobiol. 2014;27:118-26.
40. Cholia RP, Nayyar H, Kumar R, Mantha AK. Understanding the multifaceted role of ectonucleotide pyrophosphatase/phosphodiesterase 2 (ENPP2) and its altered behaviour in human diseases. Curr Mol Med. 2015;15:932-43.
41. Chuang JZ, Milner TA, Sung CH. Subunit heterogeneity of cytoplasmic dynein: differential expression of 14 kDa dynein light chains in rat hippocampus. J Neurosci. 2001;21:5501-12.
42. Collier FM, Gregorio-King CC, Gough TJ, Talbot CD, Walder K, Kirkland MA. Identification and characterization of a lymphocytic Rho-GTPase effector: rhotekin-2. Biochem Biophys Res Commun. 2004;324:1360-9.
43. Columba P, Nucera A, Zizzo C, Albeggiani G, Francofonte D, Iemolo F, Tuttolomondo A, Pinto A, Duro G. Identification of a novel mutation in the alpha-galactosidase A gene in patients with Fabry disease. Clin Biochem. 2012;45:839-41.
44. Comley LH, Fuller HR, Wishart TM, Mutsaers CA, Thomson D, Wright AK, Ribchester RR, Morris GE, Parson SH, Horsburgh K, Gillingwater TH. ApoE isoform-specific regulation of regeneration in the peripheral nervous system. Hum Mol Genet. 2011;20:2406-21.
45. Conklin DS, Galaktionov K, Beach D. 14-3-3 proteins associate with cdc25 phosphatases. Proc Natl Acad Sci USA. 1995;92:7892-6.
46. Corbi N, Batassa EM, Pisani C, Onori A, Di Certo MG, Strimpakos G, Fanciulli M, Mattei E, Passananti C. The eEF1γ subunit contacts RNA polymerase II and binds vimentin promoter regions. PLoS ONE. 2010;5:e14481.

47. Cullen JK, Abdul Murad N, Yeo A, McKenzie M, Ward M, Chong KL, Schieber NL, Parton RG, Lim YC, Wolvetang E, Maghzal GJ, Stocker R, Lavin MF. AarF domain containing kinase 3 (ADCK3) mutant cells display signs of oxidative stress, defects in mitochondrial homeostasis and lysosomal accumulation. PLoS ONE. 2016;117:e0160162.
48. Cunningham D, DeBarber AE, Bir N, Binkley L, Merkens LS, Steiner RD, Herman GE. Analysis of hedgehog signaling in cerebellar granule cell precursors in a donditional *Nsdhl* allele demonstrates an essential role for cholesterol in postnatal CNS development. Hum Mol Genet. 2015;24:2808-25.
49. Dalli J, Zhu M, Vlasenko NA, Deng B, Haeggström JZ, Petasis NA, Serhan CN. The novel 13S, 14S-epoxy-maresin is converted by human macrophages to maresin 1 (MaR1), inhibits leukotriene A4 hydrolase (LTA4H), and shifts macrophage phenotype. FASEB J. 2013;27:2573-83.
50. Danielson K, Beshara S, Qureshi AR, Heimburger O, Lindholm B, Hansson M, Hylander B, Germanis G, Stenvinkel P, Barany P. Delta-He: a novel marker of inflammation predicting mortality and ESA response in peritoneal dialysis patients. Clinical Kidney J. 2014;7:275-81.
51. Davis WJr. TheATP-binding cassette transporter (ABCA2) regulates esterification of plasma membrane cholesterol by modulation of sphingolipid metabolism. Biochim Biophys Acta. 2014;184:168-79.
52. Davison EJ, Pennington K, Hung CC, Peng J, Fariq R, Ostareck-Lederer A, Ostareck DH, Ardley HC, Banks RE, Robinson PA. Proteomic analysis of increased Parkin expression and its interactants provides evidence for a role in modulation of mitochondrial function. Proteomics. 2009;9:4284-97.
53. de Jong RCM, Pluijmert NJ, De Vries MR, Pettersson K, Atsma DE, Jukema JW, Quax PHA. Annexin A5 reduces infarct size and improves cardiac function after myocardial ischemia-reperfusion injury by suppression of the cardiac inflammatory response. Sci Rep. 2018;8:6753.
54. Deng V, Matagne V, Banine F, Frerking M, Ohliger P, Budden S, Pevsner J, Dissen GA, Sherman LS, Ojeda SR. FXYD1 is an MeCP2 target gene overexpressed in the brains of Rett syndrome patients and Mecp2-null mice. Hum Mol Genet. 2007;16:640-50.
55. Douglas MW, Diefenbach RJ, Homa FL, Miranda-Saksena M, Rixon FJ, Vittone V, Byth K, Cunningham AL. Herpes simplex virus type 1 capsid protein VP26 interacts with dynein light chains RP3 and Tctex1 and plays a role in retrograde cellular transport. J Biol Chem. 2004;279:28522-30.
56. Dovey M, Patton EE, Bowman T, North T, Goessing W, Zhou Y, Zon LI. Topoisomerase II alpha is required for embryonic development and liver regeneration in zebrafish. Mol Cell Biol. 2018;29:3746-53.
57. Du C, Wang Y, Zhang F, Yan S, Guan Y, Gong X, Zhang T, Cui X, Wang X, Zhang CX. Synaptotagmin-11 inhibits cytokine secretion and phagocytosis in microglia. Glia. 2017;65:1656-67.
58. Dutta B, Ren Y, Hao P, Sim KH, Cheow E, Adav S, Tam JP, Sze SK. Profiling of the chromatin-associated proteome identifies HP1BP3 as a novel regulator of cell cycle progression. Mol Cell Proteomics. 2014;13:2183-97.
59. Dutta B, Ren Y, Lim SK, Tam JP, Sze SK. Quantitative profiling of chromatome dynamics reveals a novel role for HP1BP3 in hypoxia-induced oncogenesis. Mol Cell Proteomics. 2014;13:3236-49.
60. Dybkaer K, Iqbal J, Zhou G, Geng H, Xiao L, Schmitz A, D'Amore F, Chan WC. Genome wide transcriptional analysis of resting and IL2 activated human natural killer cells: gene expression signatures indicative of novel molecular sigaling pathways. BMC Genomics. 2007;8:230.
61. Edbladh M, Edström A, Persson L. The role of ornithine decarboxylase and polyamines in regeneration of the frog sciatic nerve. Exp Neurol. 1990;107:63-8.
62. Efferth T. Adenosine triphosphate-binding cassette transporter genes in ageing and age-related diseases. Ageing Res Rev. 2003;2:11-24.
63. El Bassit G, Patel RS, Carter G, Shibu V, Patel AA, Song S, Murr M, Cooper DR, Bickford PC, Patel NA. MALAT1 in human adipose stem cells modulates survival and alternative splicing of PKCδII in HT22 cells. Endocrinology. 2017;158:183-95.

64. El Bounkari O, Guria A, Klebba-Faerber S, Claussen M, Pieler T, Griffiths JR, Whelton AD, Koch A, Tamura T. Nuclear localization of the pre-mRNA associating protein THOC7 depends upon its direct interaction with Fms tyrosine kinase interacting protein (FMIP). FEBS Lett. 2009;583:13-8.
65. Faast R, Thonglairoam V, Schulz TC, Beall J, Wells JR, Taylor H, Matthaei K, Rathjen PD, Tremethick DJ, Lyons I. Histone variant H2A.Z is required for early mammalian development. Curr Biol. 2001;11:1183-7.
66. Fallowfield JA, Mizuno M, Kendall TJ, Constandinou CM, Benyon RC, Duffield JS, Iredale JP. Scar-associated macrophages are a major source of hepatic matrix metalloproteinase-13 and facilitate the resolution of murine hepatic fibrosis. J Immunol. 2007;178:5288-95.
67. Fernandez-Godino R, Bujakowska KM, Pierce EA. Changes in extracellular matrix cause RPE cells to make basal deposits and activate the alternative complement pathway. Hum Mol Genet. 2018;27:147-59.
68. Fischer D, Hauk TG, Müller A, Thanos S. Crystallins of the beta/gamma-superfamily mimic the effect of lens injury and promote axon regeneration. Mol Cell Neurosci. 2008;37:471-9.
69. Friesen WJ, Dreyfuss G. Specific sequences of the Sm and Sm-like (Lsm) proteins mediate their functions with the spinal muscular atrophy disease gene product (SMN). J Biol Chem. 2000;275:26370-5.
70. Fujimara A, Kishimoto H, Yanagisawa J, Kimura K. Enhancer of rudimentary homolog (ERH) plays an essential role in the progression of mitosis by promoting mitotic chromosome alignment. Biochem Biophys Res Commun. 2012;423:588-92.
71. Furuta E, Okuda H, Kobayashi A, Watabe K. Metabolic genes in cancer: their roles in tumor progression and clinical implications. Biochim Biophys Acta. 2010;1805:141-52.
72. Gachotte D, Eckstein J, Barbuch R, Hughes T, Roberts C, Bard M. A novel gene conserved from yeast to humans is involved in sterol biosynthesis. J Lipid Res. 2001;42:150-4.
73. Gallo G, Yee HFJr, Letourneau PC. Actin turnover is required to prevent axon retraction driven by endogenous actomyosin contractility. J Cell Biol. 2002;158:1219-28.
74. Galmozzi E, Aghemo A, Colombo M. Eukaryotic initiation factor 5B: a new player for the anti-hepatitis C virus effect of ribavirin? Med Hypotheses. 2012;79:471-3.
75. Garlich J, Strecker V, Wittig I, Stuart RA. Mutational analysis of the QRRQ motif in the yeast Hig1 Type2 protein Rcf1 reveals a regulatory role for the cytochrome c oxidase complex. J Biol Chem. 2017;292:5216-26.
76. Ge J, Hu w, Zhou H, Yu J, Sun C, Chen W. Ubiquitin carboxyl-terminal hydrolase isozyme L5 inhibits human glioma cell migration and invation via downregulating SNRPF. Oncotarget. 2017;8:113635-49.
77. Geiman TM, Sankpal UT, Robertson AK, Zhao Y, Zhao Y, Roberstson KD. DNMT3B interacts with hSNF2H chromatin remodeling enzyme, HDACs 1 and 2, and components of the histone methylation system. Biochem Biophys Res Commun. 2004;318:544-55.
78. Gervasi C, Thyagarajan A, Szaro BG. Increased expression of multiple neurofilament mRNAs during regeneration of vertebrate central nervous system axons. J Comp Neurol. 2003;461:262-75.
79. Gharib SA, Johnston LK, Huizar I, Birkland TP, Hanson J, Wang Y, Parks WC, Manicone AM. MMP28 promotes macrophage polarization toward M2 cells and augments pulmonary fibrosis. J Leukoc Biol. 2014;95:9-18.
80. Glaser N, Little C, Lo W, Cohen M, Tancredi D, Wulff H, O'Donnell M. Treatment with the KCa3.1 inhibitor TRAM-34 during diabetic ketoacidosis reduces inflammatory changes in the brain. Pediatr Diabetes. 2017;18:356-66.
81. Goedeke L, Fernandez-Hernando C. microRNAs: a connection between cholesterol metabolism and neurodegeneration. Neurobiol Dis. 2014;72:48-53.
82. Gorokhova S, Bibert S, Geering K, Heintz N. A novel family of transmembrane proteins interacting with beta subunits of the Na, K-ATPase. Hum Mol Genet. 2007;16:2394-410.
83. Gralle M. The neuronal insulin receptor in its environment. J Neurochem. 2017;140:359-67.
84. Greenwold MJ, Bao W, Jarvis ED, Hu H, Li C, Gilbert MT, Zhang G, Sawyer RH. Dynamic evolution of the alpha (α) and beta (β) keratins has accompanied integument diversification and the adaptation of birds into novel lifestyles. BMC Evol Biol. 2014;14:249.

85. Grieco SF, Holmes TC, Xu X. Neuregulin directed molecular mechanisms of visual cortical plasticity. J Comp Neurol. 2018;527:668-78.
86. Guarda CCD, Santiago RP, Fiuza LM, Aleluia MM, Ferreira JRD, Figueiredo CVB, Yahouedehou SCMA, Oliveira RM, Lyra IM, Goncalves MS. Heme-mediated cell activation: the inflammotory puzzle of sickle cell anemia. Expert Rev Hematol. 2017;10:533-41.
87. Gumy LF, Tan CL, Fawcett JW. The role of local protein synthesis and degradation in axon regeneration. Exp Neurol. 2010;223:28-37.
88. Guo YY, Lu Y, Zheng Y, Chen XR, Dong JL, Yuan RR, Huang SH, Yu H, Wang Y, Chen ZY, Su B. Ubiquitin C-terminal hydrolase L1 (UCH-L1) promotes hippocampus-dependent memory via its deubiquitinating effect on TrkB. J Neurosci. 2017;37:5978-95.
89. Gupta P, Soyombo AA, Shelton JM, Wilkofsky IG, Wisniewski KE, Richardson JA, Hofmann SL. Disruption of PPT2 in mice causes an unusual lysosomal storage disorder with neurovisceral features. Proc Natl Acad Sci USA. 2003;100:12325-30.
90. Gveric D, Herrera BM, Cuzner ML. tPA receptors and the fibrinolytic response in multiple sclerosis. Am J Pathol. 2005;166:1143-51.
91. Gwee SSL, Radford RAW, Chow S, Syal MD, Morsch M, Formella I, Lee A, Don EK, Badrock AP, Cole NJ, West AK, Cheung SNS, Chung RS. Aurora kinase B regulates axonal outgrowth and regeneration in the spinal motor neurons of developing zebrafish. Cell Mol Life Sci. 2018;in press.
92. Hall D, Poussin C, Velaqapudi VR, Empsen C, Joffraud M, Beckmann JS, Geerts AE, Ravussin Y, Ifferson M, Oresic M, Thorens B. Peroxisomal and microsomal lipid pathways associated with resistance to hepatic steatosis and reduced pro-inflammatory state. J Biol Chem. 2010;285:31011-23.
93. Han W, Hu P, Wu F, Wang S, Hu Y, Li S, Jiang T, Qiang B, Peng X. FHL3 links cell growth and self-renewal by modulating SOX4 in glioma. Cell Death Differ. 2018;in press.
94. Hanlon SE, Xu Z, Norris DN, Vershon AK. Analysis of the meiotic role of the mitochondrial proteins Mrps17 and Mrpl37 in Saccharomyces cerevisiae. Yeast. 2004;21:1241-52.
95. Harnby ME, Coppola G, Ao Y, Geschwind DH, Khakh BS, Sofroniew MV. Inflammatory mediators alter the astrocyte transcriptome and calcium signaling elicited by multiple G-protein-coupled receptors. J Neurosci. 2012;32:14489-510.
96. Harris KP, Akbergenova Y, Cho RW, Baas-Thomas MS, Littleton JT. Shank modulates postsynaptic Wnt signaling to regulate synapse development. J Neurosci. 2016;36:5820-32.
97. Havlickova V, Kaplanova V, Nuskova H, Drahota Z, Houstek J. Knockdown of F1 epsilon subunit decreases mitochondrial content of ATP synthase and leads to accumulation of subunit c. Biochim Biophys Acta. 2010;1797:1124-9.
98. Hayashi S, Inoue Y, Kiyonari H, Abe T, Misaki K, Moriguchi H, Tanaka Y, Takeichi M. Protocadherin-17 mediates collective axon extension by recruiting actin regulator complexesto interaxonal contacts. Dev Cell. 2014;30:673-87.
99. Heinzel S, Riemer TG, Schulte S, Onken J, Heinz A, Rapp MA. Catechol-O-methyltransferase (COM) genotype affects age-related changes in plasticity in working memory: a pilot study. Biomed Res Int. 2014;2014:414351.
100. Henderson SJ, Weitz JI, Kim PY. Fibrinolysis: strategies to enhance the treatment of acute ischemic stroke. J Thromb Heamost. 2018;in press.
101. Henion TR, Madany PA, Faden AA, Schwarting GA. β3GnT2 null mice exhibit defective accessory olfactory bulb innervation. Mol Cell Neurosci. 2013;52:73-86.
102. Herwartz C, Castillo-Juarez P, Schröder L, Barron BL, Steger G. The transcription factor ZNF395 is required for the maximal hypoxic induction of proinflammatory cytokines in U87-MG cells. Mediators Inflamm. 2015;2015:804264.
103. Holtman IR, Bsibsi M, Gerritsen WH, Boddeke HW, Eggen BJ, van der Valk P, Kipp M, van Noort JM, Amor S. Identification of highly connected hub genes in the protective response program of human macrophages and microglia activated by alpha B-crystallin. Glia. 2017;65:460-73.
104. Honma K, Kamikubo M, Mochizuki K, Goda T. Insulin-induced inhibition of gluconeogenesis genes, including glutamic pyruvic transaminase 2, is associated with reduced histone acetylation in a human liver cell line. Metabolism. 2017;71:118-24.
105. Houlard M, Godwin J, Metson J, Lee J, Hirano T, Nasmyth K. Condensin confers the longitudinal rigidity of chromosomes. Nat Cell Biol. 2015;17:771-81.

106. Houten SM, Wanders RJ, Waterham HR. Biochemical and genetic aspects of mevalonate kinase and its deficiency. Biochim Biophys Acta. 2000;1529:19-32.
107. Howard JM, Lin H, Wallace AJ, Kim G, Draper JM, Haeussler M, Katzman S, Toloue M, Liu Y, Sanford JR. hnRNP A1 promotes recognition of splice site decoys by U2AF2 *in vivo*. Genome Res. 2018;28:689-98.
108. Huang C, Xiang Y, Wang Y, Li X, Xu L, Zhu Z, Zhang K, Jing N, Chen CD. Dual-specificity histone demethylase KIAA1718 (KDM7A) regulates neural differentiation through FGF4. Cell Res. 2010;20:154-65.
109. Huang G, Meng L, Tsai RY. p53 Configures the G2/M arrest response of nucleostemin-deficient cells. Cell Death Discov. 2015;1:15060.
110. Huang J, Schriefer AE, Yang W, Cliften PF, Rudnick DA. Identification of an epigenetic signature of early mouse liver regeneration that is disrupted by Zn-HDAC inhibition. Epigenetics. 2014;9:1521-31.
111. Huang X, He Y, Dubuc AM, Hashizume R, Zhang W, Reimand J, Yang H, Wang TA, Stehbens SJ, Younger S, et al. EAG2 potssium channel with evolutionarily conserved function as a brain tumor target. Nat Neurosci. 2015;18:1236-46.
112. Hutchins EJ, Szaro BG. c-Jun N-terminal kinase phosphorylation of heterogeneous nuclear ribonucleoprotein K regulates vertebrate axon outgrowth via a posttranscriptional mechanism. J Neurosci. 2013;33:14666-80.
113. Infantino V, Iacobazzi V, Menga A, Avantaggiati ML, Palmieri F. A key role of the mitochondrial citrate carrier (SLC25A1) in TNF-α and IFN-γ triggered inflammation. Biochim Biophys Acta. 2014;1839:1217-25.
114. Ishii T. Close teamwork between Nrf2 and peroxiredoxins 1 and 6 for the regulation of prostaglandin D2 and E2 production in macrophages in acute inflammation. Free Radic Biol Med. 2015;88 (Pt. B):189-96.
115. Ivashkiv LB, Zhao B, Park-Min KH, Takami M. Feedback inhibition of osteoclastogenesis during inflammation by IL-10, M-CSF receptor shedding, and induction of IRF8. Ann N Y Acad Sci. 2011;1237:88-94.
116. James PA, Talbot K. The molecular genetics of non-ALS motor neuron diseases. Biochim Biophys Acta. 2006;1762:986-1000.
117. Janssen U, Stoffel W. Disruption of mitochondrial beta-oxidation of unsaturated fatty acids in the 3,2-trans-enoyl-CoA isomerase-deficient mouse. J Biol Chem. 2002;277:19579-84.
118. Jaudon F, Raynaud F, Wehrle R, Bellanger JM, Doulazmi M, Vodjdani G, Gasman S, Fagni L, Dusart I, Debant A, Schmidt S. The RhoGEF DOCK10 is essential for dendritic spine morphogenesis. Mol Cell Biol. 2015;26:2112-27.
119. Jian X, Hidaka H, Schmidt JT. Kinase requirement for retinal growth cone motility. J Neurobiol. 1994;25:1310-28.
120. Jiang K, Yang Z, Cheng L, Wang S, Ning K, Zhou L, Lin J, Zhong H, Wang L, Li Y, Huang J, Zhang H, Ye Q. Mediator of ERBB2-driven cell motility (MEMO) promotes extranuclear estrogen receptor signaling involving the growth factor receptors IGF1R and ERBB2. J Biol Chem. 2013;288:24590-9.
121. Johannessen L, Sundberg TB, O'Connell DJ, Kolde R, Berstler J, Billins KJ, Khow B, et al. Small-molecule studies identify CDK8 as a regulator of IL-10 in myeloid cells. Nat Chem Biol. 2017;13:1102-8.
122. Jones EV, Bouvier DS. Astrocyte-secreted matricellular proteins in CNS remodelling during development and disease. Neural Plast. 2014;2014:321209.
123. Kahn OI, Ha N, Baird MA, Davidson MW, Baas PW. TPX2 regulates neuronal morphology through kinesin-5 interaction. Cytoskeleton (Hoboken). 2015;72:340-8.
124. Kale A, Ji Z, Kiparaki M, Blanco J, Rimesso G, Flibote S, Baker NE. Ribosomal protein S12e has a distinct function in cell competition. Dev Cell. 2018;44:42-55.
125. Kamakaka RT, Biggins S. Histone variants: deviants? Genes Dev. 2005;19:295-310.
126. Kamei N, Kwon SM, Ishikawa M, Li M, Nakanishi K, Yamada K, Hozumi K, Kawamoto A, Ochi M, Asahara T. Endothelial progenitor cells promote astrogliosis following spinal cord injury through Jagged1-dependent Notch signaling. J Neurotrauma. 2012;29:1758-69.

127. Kano G, Almanan M, Bochner BS, Zimmermann N. Mechanism of Siglec-8-mediated cell death in IL-5-activated eosinophils: role for reactive oxygen species-enhanced MEK/ERK activation. J Allergy Clin Immunol. 2013;132:437-45.
128. Kanta J. The role of hydrogen peroxide and other reactive oxygen species in wound healing. Acta Medica (Hradec Kralove). 2011;54:97-101.
129. Katayama H, Brinkley WR, Sen S. The Aurora kinases: role in cell transformation and tumorigenesis. Cancer Metastasis Rev. 2003;22:451-64.
130. Kato H, Mihara K. Identification of Tom5 and Tom6 in the preprotein translocase complex of human mitochondrial outer membrane. Biochem Biophys Res Commun. 2008;369:958-63.
131. Kawakami T, Xiao W. Phospholipase C-β in immune cells. Adv Biol Regul. 2013;53:249-57.
132. Kerr JS, Jacques RO, Moyano Cardaba C, Tse T, Sexton D, Mueller A. Differential regulation of chemotaxis: role of Gβγ in chemokine receptor-induced cell migration. Cell Signal. 2013;25:729-35.
133. Khot VV, Chavan-Gautam P, Mehendale S, Joshi SR. Variable methylation potential in preterm placenta: implication for epigenetic programming of the offspring. Reprod Sci. 2017;24:891-901.
134. Kim DW, Kim KH, Yoo BC, Hong SH, Lim YC, Shin YK, Park JG. Identification of mitochondrial F(1)F(0)-ATP synthase interacting with galectin-3 in colon cancer cells. Cancer Sci. 2008;99:1884-91.
135. Kim HR, Kwon MS, Lee S, Mun Y, Kee KS, Kim CH, Na BR, Kim BNR, Piragyte I, Lee HS, Jun Y, Jin MS, Hyun YM, Jung HS, Mun JY, Jun CD. TAGLN2 polymerizes G-actin in a low ionic state but blocks Arp2/3-nucleated actin branching in physiologic conditions. Sci Rep. 2018;8:5503.
136. King MW, Neff AW, Mescher AL. Proteomics analysis of regenerating amphibian limbs: changes during the onset of regeneration. Int J Dev Biol. 2009;53:955-69.
137. Knowlden S, Georas SN. The autotaxin-LPA axis emerges as a novel regulator of lymphocyte homing and differentiation. J Immunol. 2014;192:851-7.
138. Kobayashi T, Winslow S, Sunesson L, Hellman U, Larsson C. PKCα binds G3BP2 and regulates stress granule formation following cellular stress. PLoS ONE. 2012;7:e35820.
139. Koch JC, Tonges L, Barski E, Michel U, Bahr M, Lingor P. ROCK2 is a major regulator of axonal degeneration, neuronal death and axonal regeneration in the CNS. Cell Death Dis. 2014;5:e1225.
140. Kondou H, Kawai M, Tachikawa K, Kimoto A, Yamagata M, Koinuma T, Yamazaki M, Nakayama M, Mushiake S, Ozono K, Michigami T. Sodium-coupled neutral amino acid transporter 4 functions as a regulator of protein synthesis during liver development. Hepatol Res. 2013;43:1211-23.
141. Kong E, Peng s, Chandra G, Sarkar C, Zhang Z, Bagh MB, Mukherjee AB. Dynamic palmitoylation links cytosol-membrane shuttling of acyl-protein thioesterase-1 and acyl protein thioesterase-2 with that of proto-oncogene H-ras product and growth-associated protein-43. J Biol Chem. 2013;288:9112-25.
142. König T, Tröder SE, Bakka K, Korwitz A, Richter-Dennerlein R, Lampe PA, Patron M, Mühlmeister M, Guerrero-Castillo S, Brandt U, Decker T, Lauria I, Paggio A, Rizzuto R, Rugarli E, De Stefani D, Langer T. The m-AAA protease associated with neurodegeneration limits MCU activity in mitochondria. Mol Cell. 2016;64:148-62.
143. Kopp P, Lammers R, Aepfelbacher M, Woehlke G, Rudel T, Machuy N, Steffen W, Linder S. The kinesin KIF1C and microtubule plus ends regulate podosome dynamics in macrophages. Mol Biol Cell. 2006;17:2811-23.
144. Kraft R, Escobar MM, Narro ML, Kurtis JL, Efrat A, Barnard K, Restifo LL. Phenotypes of *Drosophila* brain neurons in primary culture reveal a role for fascin in neurite shape and trajectory. J Neurosci. 2006;26:8734-47.
145. Kreko-Pierce T, Eaton BA. The *Drosophila* LC8 homolog *cut up* specifies the axonal transport of proteasomes. J Cell Sci. 2017;130:3388-98.
146. Kruzel ML, Zimecki M, Actor JK. Lactoferrin in a context of inflammation-induced pathology. Front Immunol. 2017;8:1438.
147. Kurimoto T, Yin Y, Habboub G, Gilbert HY, Li Y, Nakao S, Hafezi-Moghadam A, Benowitz LJ. Neutrophils express oncomodulin and promote optic nerve regeneration. J Neurosci. 2013;33:14816-24.

148. Lacerda R, Menezes J, Romão L. More than just scanning: the importance of cap-independent mRNA translation initiation for cellular stress response and cancer. Cell Mol Life Sci. 2017;74:1659-80.
149. Lacor P, Gandolfo P, Tonon MC, Brault E, Dalbert I, Schumacher M, Benavides J, Ferzaz B. Regulation of the expression of peripheral benzodiazepine receptors and their endogenous ligands during rat sciatic nerve degeneration and regeneration: a role for PBR in neurosteroidogenesis. Brain Res. 1999;815:70-80.
150. Lai M, Wang F, Rohan JG, Maeno-Hikichi Y, Chen Y, Zhou Y, Gao G, Sather WA, Zhang JF. A tctex1-Ca^{2+} channel complex for selective surface expression of Ca^{2+} channels in neurons. Nat Neurosci. 2005;8:435-42.
151. Laing NG, Dye DE, Wallgren-Pettersson C, Richard G, Monnier N, Lillis S, Winder TL, Lochmuller H, Graziano C, Mitrani-Rosenbaum S, Twomey D, Sparrow JC, Beggs AH, Nowak KJ. Mutations and polymorphisms of the skeletal muscle alpha-actin gene (ACTA1). Hum Mutat. 2009;30:1267-77.
152. Larhammar M, Huntwork-Rodriguez S, Rudhard Y, Sengupta-Ghosh A, Lewcock JW. The Ste20 family kinases MAP4K4, MINK1, and TNIK converge to regulate stress-induced JNK signaling in neurons. J Neurosci. 2017;37:11074-84.
153. Lawrence KS, Chau T, Engebrecht J. DNA damage response and spindle assembly checkpoint function throughout the cell cycle to ensure genomic integrity. PLoS Genet. 2015;11:e1005150.
154. Leduc MS, Mcguire M, Madan-Khetarpal S, Ortiz D, Hayflick S, Keller K, Eng CM, Yang Y, Bi W. De novo apparent loss-of-function mutations in PRR12 in three patients with intellectual disability and iris abnormalities. Hum Genet. 2018;137:257-64.
155. Lee HK, Chaboub LS, Zhu W, Zollinger D, Rasband MN, Fancy SP, Deneen B. Daam2-PIP5K is a regulatory pathway for Wnt signaling and therapeutic target for remyelination in the CNS. Neuron. 2015;85:1227-43.
156. Lee JC, Park JH, Kim IH, Cho GS, Ahn JH, Tae HJ, Choi SY, Cho JH, Kim DW, Kwon YG, Kang IJ, Won MH, Kim YM. Neuroprotection of ischemic preconditioning is mediated by thioredoxin 2 in the hippocampus CA1 region following a subsequent transient cerebral ischemia. Brain Pathol. 2017;27:276-91.
157. Lee PT, Chao PK, Ou LC, Chuang JY, Lin YC, Chen SC, Chang HF, Law PY, Loh HH, Chao YS, Su TP, Yeh SH. Morphine drives internal ribosome entry site-mediated hnRNP K translation in neurons through opioid receptor-dependent signaling. Nucl Acids Res. 2014;42:13012-25.
158. Lei M. The MCM complex: its role in DNA replication and implications for cancer therapy. Curr Cancer Drug Targets. 2005;5:365-80.
159. Lenkowski JR, Qin Z, Sifuentes CJ, Thummel R, Soto CM, Moens CB, Raymond PA. Retinal regeneration in adult zebrafish requires regulation of TGFβ signaling. Glia. 2013;61:1687-97.
160. Li D, Ni H, Rui Q, Gao R, Chen G. Mst1: function and mechanism in brain and myocardial ischemia reperfusion injury. Curr Neuropharmacol. 2018;in press:-zhao.
161. Li HP, Chen PG, Liu FT, Zhu HS, Jiao XQ, Zhong K, Guo YJ, Zha GM, Han LQ, Lu WF, Wang YY, Yang GY. Characterization and anti-inflammation role of swine IFITM3 gene. Oncotarget. 2017;8:73579-89.
162. Li X, Tian r, Gao R, Yang Y, Williams BRG, Gantier MP, McMillan NAJ, Xu D, Hu Y, Gao Y. Identification of a histone family gene signature for predicting the prognosis of cervical cancer patients. Sci Rep. 2017;7:16495.
163. Liang D, Li J, Wu Y, Zhen L, Li C, Qi M, Wang L, Deng F, Huang J, Lv F, Liu Y, Ma X, Yu Z, Zhang Y, Chen YH. miRNA-204 drives cardiomyocyte proliferation via targeting JARID2. Int J Cardiol. 2015;201:38-48.
164. Liao D. Emerging roles of the EBF family of transcription factors in tumor suppression. Mol Cancer Res. 2009;7:1893-901.
165. Lin F, Gao L, Su Z, Cao X, Zhan Y, Li Y, Zhang B. Knockdown of KPNA2 inhibits autophagy in oral squamous cell carcinoma cell lines by blocking p53 nuclear translocation. Oncol Rep. 2018;40:179-94.
166. Lin HJ, Shaffer KM, Sun Z, Jay G, He WW, Ma W. Af1q, a differentially expressed gene during neuronal differentiation, transforms HEK cells into neuron-like cells. Brain Res Mol Brain Res. 2004;131:126-30.

167. Lin L, Ke Z, Kv M, Lin R, Wu B, Zheng B. Effects of $MgSO_4$ and magnesium transporters on 6-hydroxydopamine-induced SH-SY5Y cells. Life Sci. 2017;172:48-54.
168. Lin S, Liu M, Son YJ, Himes BT, Yu W, Baas PW. Inhibition of kinesin-5, a microtubule-based motor protein, as a strategy for enhancing regeneration of adult axons. Traffic. 2011;12:269-86.
169. Lin X, Wang R, Zhang J, Sun X, Zou Z, Wang S, Jin M. Insights into human astrocyte response to H5N1 infection by microarray analysis. Viruses. 2015;7:2618-40.
170. Linossi EM, Nicholson SE. The SOCS box – adapting proteins for ubiquitination and proteasomal degradation. IUBMB Life. 2012;64:316-23.
171. Liu H, Nakagawa T, Kanematsu T, Uchida T, Tsuji S. Isolation of 10 differentially expressed cDNAs in differentiated Neuro2a cells induced through contolled expression of the GD3 synthase gene. J Neurochem. 1999;72:1781-90.
172. Liu L, Aldskogius H, Svensson M. Ultrastructural localization of immunoglobulin G and complement c9 in the brain stem and spinal cord following peripheral nerve injury: an immunoelectron microscopic study. J Neurocytol. 1998;27:737-48.
173. Liu Y, Szaro BG. hnRNP K post-transcriptionally co-regulates multiple cytoskeletal genes needed for axonogenesis. Development. 2011;138:3079-90.
174. Liu Y, Wang C, Destin G, Szaro BG. Microtubule-associated protein tau promotes neuronal class II β-tubulin microtubule formation and axon elongation in embryonic *Xenopus laevis*. Eur J Neurosci. 2015;41:1263-75.
175. Liu Y, Yu H, Deaton SK, Szaro BG. Heterogeneous ribonucleoprotein K, an RNA-binding protein, is required for optic axon regeneration in *Xenopus laevis*. J Neurosci. 2012;32:3563-74.
176. LLanos S, Serrano M. Depletion of ribosomal protein L37 occurs in response to DNA damage and activates p53 through the L11/MDM2 pathway. Cell Cycle. 2010;9:4005-12.
177. Löffler K, Schäfer P, Völkner M, Holdt T, Karl MO. Age-dependent Müller glia neurogenic competence in the mouse retina. Glia. 2015;63:1809-24.
178. Lonati C, Carlin A, Leonardi P, Valenza F, Bosari S, Catania A, Gatti S. Modulatory effects of NDP-MSH in the regenerating liver after partial hepatectomy in rats. Peptides. 2013;50:145-52.
179. Lüders KA, Patzig J, Simons M, Nave KA, Werner HB. Genetic dissection of oligodendroglial and neuronal Plp1 function in a novel mouse model of spastic paraplegia type 2. Glia. 2017;65:1762-76.
180. Lutz D, Kararia H, Kleene R, Loers G, Chaudhary H, Guseva D, Wu B, Jakovcevski I, Schachner M. Myelin basic protein cleaves cell adhesion molecule L1 and improves regeneration after injury. Mol Neurobiol. 2016;53:3360-76.
181. Ma RY, Tam TS, Suen AP, Yeung PM, Tsang SW, Chung SK, Thomas MK, Leung PS, Yao KM. Secreted PDZD2 exerts concentration-dependent effects on the proliferation of INS-1E cells. Int J Biochem Cell Biol. 2006;38:1015-22.
182. Majka J, Burgers PM. The PCNA-RFC families of DNA clamps and clamp loaders. Prog Nucl Acid Res Mol Biol. 2004;78:227-60.
183. Maki N, Kimura H. Epigenetics and regeneration. Curr Top Microbiol Immunol. 2013;367:237-52.
184. Makiyama T, Akao M, Haruna Y, Tsuji K, Doi T, Ohno S, Nishio Y, Kita T, Horie M. Mutation analysis of the glycerol-3 phosphate dehydrogenase-1 like (GPD1L) gene in Japanese patients with Brugada syndrome. Circ J. 2008;72:1705-6.
185. Malaspina A, Kaushik N, de Belleroche J. A 14-3-3 mRNA is up-regulated in amyotrophic lateral sclerosis spinal cord. J Neurochem. 2000;75:2511-20.
186. Malodobra-Mazur M, Dziewulska A, Kozinski K, Dobrzyn P, Kolczynska K, Janikiewicz J, Dobrzyn A. Stearoyl-CoA desaturase regulates inflammatory gene expression by changing DNA methylation level in 3T3 adipocytes. Int J Biochem Cell Biol. 2014;55:40-50.
187. Mandai K, Rikitake Y, Mori M, Takai Y. Nectins and nectin-like molecules in development and disease. Curr Top Dev Biol. 2015;112:197-231.
188. Marco A, Cuesta A, Pedrola L, Palau F, Marin I. Evolutionary and structural analyses of GDAP1, involved in Charcot-Marie-Tooth disease, characterize a novel class of glutathione transferase-related genes. Mol Biol Evol. 2004;21:176-87.
189. Marshall JJ, Xu J, Contractor A. Kainate receptors inhibit glutamate release via mobilization of endocannabinoids in striatal direct pathway spinal projection neurons. J Neurosci. 2018;38:3901-10.

190. Martinez de Paz A, Ausio J. HMGNs: the enhancer charmers. BioEssays. 2016;38:226-31.
191. Martinez-De Luna RI, Ku RY, Aruck AM, Santiago F, Viczian AS, San Mauro D, Zuber ME. Müller glia reactivity follows retinal injury despite the absence of the glial fibrillary acidic protein gene in *Xenopus*. Dev Biol. 2017;426:219-35.
192. Masterson L, Sorgeloos F, Winder D, Lechner M, Marker A, Malhotra S, Sudhoff H, Jani P, Goon P, Sterling J. Deregulation of SYCP2 predicts early stage human papillomavirus-positive oropharyngeal carcinoma: a prospective whole transcriptome analysis. Cancer Sci. 2015;106:1568-75.
193. Masuda T, Wada Y, Kawamura S. ES1 is a mitochondrial enlarging factor contributing to form mega-mitochondria in zebrafish cones. Sci Rep. 2016;6:22360.
194. Matsuda S, Kawamoto K, Miyamoto K, Tsuji A, Yuasa K. PCTK3/CDK18 regulates cell migration and adhesion by negatively modulating FAK activity. Sci Rep. 2017;7:45545.
195. McEwan J, Lynch J, Beck CW. Expression of key retinoic acid modulating genes suggests active regulation during development and regeneration of the amphibian limb. Dev Dyn. 2011;240:1259-70.
196. McIntyre JC, Titlow WB, McClintock TS. Axon growth and guidance genes identify nascent, immature, and mature olfactory sensory neurons. J Neurosci Res. 2010;88:3243-56.
197. McLaughlin B, Hartnett KA, Erhardt JA, Legos JJ, White RF, Barone FC, Aizenman E. Caspase 3 activation is essential for neuroprotection in preconditioning. Proc Natl Acad Sci U S A. 2003;100:715-20.
198. McLin VA, Hu CH, Shah R, Jamrich M. Expression of complement components coincides with early patterning and organogenesis in *Xenopus laevis*. Int J Dev Biol. 2008;52:1123-33.
199. McNeely KC, Cupp TD, Little JN, Janish KM, Shrestha A, Dwyer ND. Mutation of Kinesin-6 Kif20b causes defects in cortical neuron polarization and morphogenesis. Neural Dev. 2017;12:5.
200. Menoret A, Kumar S, Vella AT. Cytochrome b5 and cytokeratin 17 are biomarkers in bronchoalveolar fluid signifying onset of acute lung injury. PLoS ONE. 2012;7:e40184.
201. Miao T, Wu D, Wheeler A, Wang P, Zhang Y, Bo X, Yeh JS, Subang MC, Richardson PM. Two cytokine signaling molecules co-operate to promote axonal transport and growth. Exp Neurol. 2011;228:165-72.
202. Miao T, Wu D, Zhang Y, Bo X, Subang MC, Wang P, Richardson PM. Suppressor of cytokine signaling-3 suppresses the ability of activated signal transducer and activator of transcription-3 to stimulate neurite growth in rat primary sensory neurons. J Neurosci. 2006;26:9512-9.
203. Milan-Zambrano G, Chavez S. Nuclear functions of prefoldin. Open Biol. 2014;4:140085.
204. Miller CM, Liu N, Page-McCaw A. Drosophila MMP2 regulates the matrix molecule faulty attraction (Frac) to promote motor axon targeting in *Drosophila*. J Neurosci. 2011;31:5335-47.
205. Minoura I. Towards an understanding of the isotype-specific functions of tubulin in neurons: technical advances in tubulin expression and purification. Neurosci Res. 2017;122:1-8.
206. Miyamoto S, Murphy AN, Brown JH. Akt mediates mitochondrial protection in cardiomyocytes through phosphorylation of mitochondrial hexokinase-II. Cell Death Diff. 2007;15:521-9.
207. Mocchegiani E, Malavolta M. Zinc dyshomeostasis, ageing and neurodegeneration: implications of A2M and inflammatory gene polymorphisms. J Alzheimers Dis. 2007;12:101-109mo.
208. Mohammed TO, Chagan-Yasutan H, Ashino Y, Nakayama W, Takahashi Y, Shimomura T, Fujimoto T, Watanabe Y, Niki T, Suzushima H, Hattori T. Galectin-9 as a predictive marker for the onset of immune-related adverse effects associated with anti-CCR4 MoAb therapy in patients with adult T cell leukemia. Tohuku J Exp Med. 2017;241:201-8.
209. Mohun TJ, Garrett N, Gurdon JB. Upstream sequences required for tissue-specific activation of the cardiac actin gene in *Xenopus laevis* embryos. EMBO J. 1986;5:3185-93.
210. Montenegro G, Rebelo AP, Connell J, Allison R, Babalini C, D'Aloia M, Montieri P, Schule R, Ishiura H, Price J, Strickland A, Gonzalez MA, Baumbach-Reardon L, Deconinck T, Huang J, Bernardi G, Vance JM, Rogers MT, Tsuji S, De Jonghe P, Pericak-Vance MA, Schols L, Orjaccio A, Reid E, Zuchner S. Mutations in the ER-shaping protein reticulon 2 cause the axon-degenerative disorder hereditary spastic paraplegia type 12. J Clin Invest. 2012;122:538-44.
211. Moody SA, Miller V, Spanos A, Frankfurter A. Developmental expression of a neuron-specific beta-tubulin in frog (*Xenopus laevis*): a marker for growing axons during the embryonic period. J Comp Neurol. 1996;364:219-30.

212. Mossi R, Jonsson ZO, Allen BL, Hardin SH, Hübscher U. Replication factor C interacts with the C-terminal side of proliferating cell nuclear antigen. J Biol Chem. 1997;272:1769-76.
213. Munoz J, Dhillon N, Janku F, Watowich SS, Hong DS. STAT3 inhibitors: finding a home in lymphoma and leukemia. Oncologist. 2014;19:536-44.
214. Munoz J, Dhillon N, Janku F, Watowich SS, Hong DS. STAT3 inhibitors: finding a home in lymphoma and leukemia. Oncologist. 2014;19:536-44.
215. Muro Y, Yamada T, Himeno M, Sugimoto K. cDNA cloning of a novel autoantigen targeted by a minor subset of anti-centromere antibodies. Clin Exp Immunol. 1998;111:372-6.
216. Mussil B, Suspene R, Aynaud MM, Gauvrit A, Vartanian JP, Wain-Hobson S. Human APOBEC3A isoforms translocate to the nucleus and induce DNA double strand breaks leading to cell stress and death. PLoS ONE. 2013;8:e73641.
217. Myung K, Smith S, Kolodner RD. Mitotic checkpoint function in the formation of gross chromosomal rearrangements in *Saccharomyces cerevisiae*. Proc Natl Acad Sci U S A. 2004;101:15980-5.
218. Naito Y, Takagi T, Higashimura Y. Heme oxygenase-1 and anti-inflammatory M2 macrophages. Arch Biochem Biophys. 2014;564:83-8.
219. Namekata K, Harada C, Tava C, Guo X, Kimura H, Parada LF, Harada T. Dock3 induces axonal outgrowth by stimulating membrane recruitment of the WAVE complex. Proc Natl Acad Sci USA. 2010;107:7586-91.
220. Namekata K, Kimura A, Kawamura K, Harada C, Harada T. Dock GEFs and their therapeutic potential: neuroprotection and axon regeneration. Prog Retin Eye Res. 2014;43:1-16.
221. Nandagopal N, Santat LA, LeBon L, Sprinzak D, Bronner ME, Elowitz MB. Dynamic ligand discrimination in the Notch signaling pathway. Cell. 2018;172:869-80.
222. Nasoohi S, Ismael S, Ishrat T. Thioredoxin-interacting protein (TXNIP) in cerebrovascular and neurodegenerative diseases: regulation and implication. Mol Neurobiol. 2018;55:7900-20.
223. Nguyen VQ, Co C, Irie K, Li JJ. Clb/Cdc28 kinases promote nuclear export of the replication initiator proteins Mcm2-7. Curr Biol. 2000;10:195-205.
224. Nie L, Guo X, Esmailzadeh L, Zhang J, Asadi A, Collinge M, Li X, Kim JD, Wools M, Jin SW, Dubrac A, Eichmann A, Simons M, Bender JR, Sadeghi MM. Transmembrane protein ESDN promotes endothelial VEGF signaling and regulates angiogenesis. J Clin Invest. 2013;123:5082-97.
225. Noor NM, Steer DL, Wheaton BJ, Ek CJ, Truettner JS, Dietrich WD, Dziegielewska KM, Richardson SJ, Smith AI, BandeBerg JL, Saunders NR. Age-dependent changes in the proteome following complete spinal cord transection in a postnatal South American opossum (*Monodelphis domestica*). PLoS ONE. 2011;6:e27465.
226. Norsworthy MW, Bei F, Kawaguchi R, Sanes JR, Coppola G, He Z. Sox11 expression promotes regeneration of some retinal ganglion cell types but kills others. Neuron. 2017;94:1112-20.
227. Nuckels RJ, Ng A, Darland T, Gross JM. The vacuolar-ATPase complex regulates retinoblast proliferation and survival, photoreceptor morphogenesis, and pigmentation in the zebrafish eye. Invest Ophthalmol Vis Sci. 2009;50:893-905.
228. O'Leary MN, Schreiber KH, Zhang Y, Duc AC, rao S, Hale JS, Academia EC, Shah SR, Morton JF, Holstein CA, Martin DB, Kaeberlein M, Ladiges WC, Fink PJ, MacKay VL, Wiest DL, Kennedy BK. The ribosomal protein Rpl22 controls ribosome composition by directly repressing expression of its own paralog, Rpl221L1. PLoS Genet. 2013;9:e1003708.
229. Oghumu S, Terrazas CA, Varikuti S, Kimble J, Vadia S, Yu L, Seveau S, Satoskar AR. CXCR3 expression defines a novel subset of innate CD8+ T cells that enhance immunity against bacterial infection and cancer upon stimulation with IL-15. FASEB J. 2015;29:1019-28.
230. Ohsawa S, Hamada S, Kuida K, Yoshida H, Igaki T, Miura M. Maturation of sensory neurons by Apaf-1/caspase-9-mediated caspase activity. Proc Natl Acad Sci USA. 2010;107:13366-71.
231. Oliva AA, Jr., Atkins CM, Copenagle L, Banker GA. Activated c-Jun N-terminal kinase is required for axon formation. J Neurosci. 2006;26:9462-70.
232. Oppikofer M, Bai T, Gan Y, Haley B, Liu P, Sandoval W, Ciferri C, Cochran AG. Expansion of the ISWI chromatin remodeler family with new active complexes. EMBO Reports. 2017;18:1697-706.

233. Otsmane B, Moumen A, Aebischer J, Coque E, Sar C, Sunyach C, Salsac C, Valmier J, Salinas S, Bowerman M, Raoul C. Somatic and axonal LIGHT signaling elicit degenerative and regenerative responses in motoneurons, respectively. EMBO Rep. 2014;15:540-7.
234. Palpant NJ, Pabon L, Rabinowitz JS, Hadland BK, Stoick-Cooper CL, Paige SL, Bernstein ID, Moon RT, Murry CE. Transmembrane protein 88: a Wnt regulatory protein that specifies cardiomyocyte development. Development. 2013;140:3799-808.
235. Pannone BK, Kim SD, Noe DA, Wolin SL. Mutiple functional interactions between components of the Lsm2-8 complex, U6 snRNA, and the yeast La protein. Genetics. 2001;158:187-96.
236. Papastefanaki F, Chen J, Lavdas AA, Thomaidou D, Schachner M, Matsas R. Graft of Schwann cells engineered to express PSA-NCAM promote functional recovery after spinal cord injury. Brain. 2007;130 (Pt 8):2159-74.
237. Park I, Han C, Jin S, Lee B, Choi H, Kwon JT, Kim D, Kim J, Lifirsu E, Park WJ, Park ZY, Kim DH, Cho C. Myosin regulatory light chains are required to maintain the stability of myosin II and cellular integrity. Biochem J. 2011;434:171-80.
238. Park J, Kim S, Joh J, remick SC, Miller DM, Yan J, Kanaan Z, Chao JH, Krem MM, Basu SK, Hagiwara S, Kenner L, Moriggl R, Bunting KD, Tse W. MLLT11/AF1q boosts oncogenic STAT3 activity through Src-PDGFR tyrosine kinase signaling. Oncotarget. 2018;2016:43960-73.
239. Park SH, Yu M, Kim J, Moon Y. C/EBP homologous protein promotes NSAID-activated gene 1-linked pro-inflammatory signals and enterocyte invasion by enteropathogenic *Escherichia col*. Microbes Infect. 2017;19:110-21.
240. Pastrana E, Moreno-Flores MT, Gurzov EN, Avila J, Wandosell F, Diaz-Nido J. Genes associated with adult axon regeneration promoted by olfactory ensheathing cells: a new role for matrix metalloproteinase 2. J Neurosci. 2006;26:5347-59.
241. Pathak GK, Ornstein H, Aranda-Espinoza H, Karlsson AJ, Shah SB. Increases in retrograde injury signaling complex-related transcripts in central axons following injury. Neural Plast. 2016;2016:3572506.
242. Pathania R, Ramachandran S, Mariappan G, Thakur P, Shi H, Choi JH, Manicassamy S, Kolhe R, Prassad PD, Sharma S, Lokeshwar BL, Ganapathy V, Thangaraju M. Combined inhibition of DNMT and HDAC blocks the tumorigenicity of cancer stem-like cells and attenuates mammary tumor growth. Cancer Res. 2016;76:3224-35.
243. Paul V, Tonchev AB, Henningfeld KA, Pavlakis E, Rust B, Pieler T, Stoykova A. Scratch2 modulates neurogenesis and cell migration through antagonism of bHLH proteins in the developing neocortex. Cereb Cortex. 2014;24:754-72.
244. Pavel M, Imarisio S, Menzies FM, Jimenez-Sanchez M, Siddiqi FH, Wu X, Renna M, O'Kane CJ, Crowther DC, Rubinsztein DC. CCT complex restricts neuropathogenic protein aggregation via autophagy. Nat Commun. 2016;7:13821.
245. Pedrosa E, Ye K, Nolan KA, Morrell L, Okun JM, Persky AD, Saito T, Lachman HM. Positive association of schizophrenia to JARID2 gene. Am J Med Genet B Neuropsychiatr Genet. 2007;144B:45-51.
246. Pei Z, Jia Z, Watkins PA. The second member of the human and murine bubblegum family is a testis- and brainstem-specific acyl-CoA sythetase. J Biol Chem. 2006;281:6632-41.
247. Penas C, Navarro X. Epigenetic modifications associated to neuroinflammation and neuropathic pain after neural trauma. Front Cell Neurosci. 2018;12:158.
248. Perucho L, Artero-Castro A, Guerrero S, Ramon y Cajal S, Lleonart ME, Wang ZQ. RPLP1, a crucial ribosomal protein for embryonic development of the nervous system. PLoS ONE. 2014;9:e99956.
249. Pfefferli C, Müller F, Jazwinska A, Wicky C. Specific NuRD components are required for fin regeneration in zebrafish. BMC Biol. 2014;12:30.
250. Pfeiffer JR, McAvoy BL, Fecteau RE, Deleault KM, Brooks SA. CARHSP1 is required for effective tumor necrosis factor alpha mRNA stabilization and localizes to processing bodies and exosomes. Mol Cell Biol. 2011;31:277-86.
251. Priscilla R, Szaro BG. Comparisons of SOCS mRNA and protein levels in *Xenopus* provide insights into optic nerve regenerative success. Brain Res. 2019;1704:150-60.

252. Qiao Y, Tang C, Wang Q, Wang D, Yan G, Zhu B. Kir2.1 regulates rat smooth muscle cell proliferation, migration, and post-injury carotid neointimal formation. Biochem Biophys Res Commun. 2016;477:774-80.
253. Raisner RM, Hartley PD, Meneghini MD, Bao MZ, Liu CL, Schreiber SL, Rando OJ, Madhani HD. Histone variant H2A.Z marks the 5' ends of both active and inactive genes in euchromatin. Cell. 2005;123:223-48.
254. Rajiv C, Jackson SR, Cocklin S, Eisenmesser EZ, Davis TL. The spliceosomal proteins PPIH and PRPF4 exhibit bi-partite binding. Biochem J. 2017;474:3689-704.
255. Reilly PT, Yu Y, Hamiche A, Wang L. Cracking the ANP32 whips: important functions, unequal requirement, and hints at disease implications. BioEssays. 2014;36:1062-71.
256. Renault-Mihara F, Mukaino M, Shinozaki M, Kumamaru H, Kawase S, Baudoux M, Ishibashi T, Kawabata S, Nishiyama Y, Sugai K, Yasutake K, Okada S, Nakamura M, Okano H. Regulation of RhoA by STAT3 coordinates glial scar formation. J Cell Biol. 2017;216:2533-50.
257. Rensen SS, Slaats Y, Driessen A, Peutz-Koostra CJ, Nijhuis J, Steffensen R, Greve JW, Buurman WA. Activation of the complement system in human nonalcoholic fatty liver disease. Hepatology. 2009;50:1809-17.
258. Roberson S, Halpern ME. Convergence of signaling pathways underlying habenular formation and axonal outgrowth in zebrafish. Development. 2017;144:2652-62.
259. Rodriguez de Cordoba S, Esparza-Gordillo J, Goicoechea de Jorge E, Lopez-Trascasa M, Sanchez-Corral P. The human complement factor H: functional roles, genetic variations and disease associations. Mol Immunol. 2004;41:355-67.
260. Roobol A, Roobol J, Carden MJ, Smith ME, Hershey JW, Bastide A, Knight JR, Willis AE, Smales CM. The chaperonin CCT interacts with and mediates the correct folding and activity of three subunits of translation initiation factor eIF3: b, i, and h. Biochem J. 2014;458:213-24.
261. Rot I, Baguma-Nibasheka M, Costain WJ, Hong P, Tafra R, Mardesic-Brakus S, Mrduljas-Djujic N, Saraga-Babic M, Kablar B. Role of skeletal muscle in ear development. Histol Histopathol. 2017;32:987-1000.
262. Rousseau E, Michel PP, Hirsch EC. The iron-binding protein lactoferrin protects vulnerable dopamine neurons from degeneration by preserving mitochondrial calcium homeostasis. Mol Pharmacol. 2013;84:888-98.
263. Saeedi Saravi SS, Saeedi Saravi SS, Arefidoust A, Dehpour AR. The benefits of HMG-CoA reductase inhibitors in the processes of neurodegeneration. Metab Brain Dis. 2017;32:949-65.
264. Sandgren J, Holm S, Marino AM, Asmundsson J, Grillner P, Nister M, Diaz de Stahl T. Whole exome- and mRNA-sequencing of an AT/RT case reveals few somatic mutations and several deregulated signalling pathways in the context of SMARCB1 deficiency. Biomed Res Int. 2015;2015:862039.
265. Sanyal R, Polyak MJ, Zuccolo J, Puri M, Deng L, Roberts L, Zuba A, Storek J, Luider JM, Sundberg EM, Mansoor A, Baigorri E, Chu MP, Belch AR, Pilarski LM, Deans JP. MS4A4A: a novel cell surface marker for M2 macrophages and plasma cells. Immunol Cell Biol. 2017;95:611-9.
266. Saurin AT. Kinase and phosphatase cross-talk at the kinetechore. Front Cell Dev Biol. 2018;6:62.
267. Saxena S, Singh SK, Lakshmi MG, Meghah V, Bhatti B, Swamy CV, Sundaram CS, Idris MM. Proteomic analysis of zebrafish caudal fin regeneration. Mol Cell Proteomics. 2012;11:M111.014118.
268. Schleich S, Strassburger K, Janiesch PC, Koledachkina T, Miller KK, Haneke K, Cheng Ys, Kuechler K, Stoecklin G, Duncan KE, Teleman AA. DENR-MCT-1 promotes translation re-initiation downstream of uORFs to control tissue growth. Nature. 2014;512:208-12.
269. Schuppan D, Ruehl M, Somasundaram R, Hahn EG. Matrix as a modulator of hepatic fibrogenesis. Semin Liver Dis. 2001;21:351-72.
270. Scott DD, Oeffinger M. Nucleolin and nucleophosmin: nucleolar proteins with multiple functions in DNA repair. Biochem Cell Biol. 2016;94:419-32.
271. Shaw T, Martin P. Epigenetic reprogramming during wound healing: loss of polycomb-mediated silencing may enable upregulation of repair genes. EMBO Rep. 2009;10:881-6.

272. Shelton LB, Baker JD, Zheng D, Sullivan LE, Solanki PK, Webster JM, Sun Z, Sabbagh JJ, Nordhues BA, Koren J3, Ghosh S, Blagg BSJ, Blair LJ, Dickey CA. Hsp90 activator Aha1 drives production of pathological tau aggregates. Proc Natl Acad Sci U S A. 2017;114:9707-12.
273. Shi R, Redman P, Ghose D, Liu Y, Ren X, Ding L, Liu M, Jones KJ, Xu W. Shank proteins differentially regulate synaptic transmission. eNeuro. 2017;4:ENEURO0.163-15.2017.
274. Shin JE, Miller BR, Babetto E, Cho Y, Sasaki Y, Qayum S, Russler EV, Cavalli V, Milbrandt J, DiAntonio A. SCG10 is a JNK target in the axonal degeneration pathway. Proc Natl Acad Sci U S A. 2012;109:E3696-E3705.
275. Sitko JC, Guevara CI, Cacalano NA. Tyrosine-phosphorylated SOCS3 interacts with the Nck and Crk-L adapter proteins and regultes Nck activation. J Biol Chem. 2004;279:37662-9.
276. Soboleva TA, Parker BJ, Nekrasov M, Hart-Smith G, Tay YJ, Tnq WQ, Wilkins M, Ryan D, Tremethick DJ. A new link between transcriptional initiation and pre-mRNA splicing: the RNA binding histone variant H2A.B. PLoS Genet. 2017;13:e1006633.
277. Soler-Botija C, Forcales SV, Genis AB. Spotlight on epigenetic reprogramming in cardiac regeneration. Semin Cell Dev Biol. 2019;S1084-9521:30240-4.
278. Song W, Cho Y, Watt D, Cavalli V. Tubulin-tyrosine Ligase (TTL)-mediated increase in tyrosinated α-tubulin in injured axons is required for retrograde injury signaling and axon regeneration. J Biol Chem. 2015;290:13344-53.
279. Sotiropoulos I, Galas MC, Silva JM, Skoulakis E, Wegmann S, Maina MB, Blum D, Savas CL, Mandelkow EM, Mandelkow E, Spillantini MG, Soussa N, Avila J, Medina M, Mucher A, Buee L. Atypical, non-standard functions of the microtubule associated Tau protein. Acta Neuropathol Commun. 2017;5:91.
280. Sotoda Y, Negoro M, Wakabayashi I. Involvement of decreased myo-inositol transport in lipopolysaccharide-induced depression of phosphoinositide hydrolysis in vascular smooth muscle. FEBS Lett. 2002;519:227-30.
281. Soyombo AA, Hofmann SL. Molecular cloning and expression of palmitoyl-protein thioesterase 2 (PPT2), a homolog of lysosomal palmitoyl-protein thioesterase with a distinct substrate specificity. J Biol Chem. 1997;272:27456-63.
282. Spead O, Verreet T, Donelson CJ, Poulain FE. Characterization of the caspase family in zebrafish. PLoS ONE. 2018;13:e0197966.
283. Stern S, Debre E, Stritt C, Berger J, Posern G, Knoll B. A nuclear actin function regulates neuronal motility by serum response factor-dependent gene transcription. J Neurosci. 2009;29:4512-8.
284. Stoeckli ET. Protocadherins: not just neuron glue, more too! Dev Cell. 2014;30:643-4.
285. Sun M, Ahmad N, Zhang R, Graw J. *Crybb2* associates with *Tmsb4x* and is crucial for dendrite morphogenesis. Biochem Biophys Res Commun. 2018;in press.
286. Sunryd JC, Cheon B, Graham JB, Giora KM, Fissore RA, Hebert DN. TMTC1 and TMTC2 are novel endoplasmic reticulum tetratricopeptide repeat-containing adpater proteins involved in calcium homeostasis. J Biol Chem. 2014;289:16085-99.
287. Taleski G, Sontag E. Protein phosphatase 2A and tau: an orchestrated 'Pas de Deux'. FEBS Lett. 2018;592:1079-95.
288. Tang S, Chen T, Yu Z, Zhu X, Yang M, Xie B, Li N, Cao X, Wang J. RasGRP3 limits Toll-like receptor-triggered inflammatory response in macrophages by activating Rap1 small GTPase. Nat Commun. 2014;5:4657.
289. Tang YP, Wade J. Sexually dimorphic expression of the genes encoding ribosomal proteins L17 and L37 in the song control nuclei of juvenile zebra finches. Bran Res. 2006;1126:102-8.
290. Tannahill GM, Elliott J, Barry AC, Hibbert L, Cacalano NA, Johnston JA. SOCS2 can enhance interleukin-2 (IL-2) and IL-3 signaling by accelerating SOCS3 degradation. Mol Cell Biol. 2005;25:9115-26.
291. Taylor WR, Stark GR. Regulation of the G2/M transition by p53. Oncogene. 2001;20:1803-15.
292. Thoms M, Thomson E, Baßler J, Gnädig M, Griesel S, Hurt E. The exosome is recruited to RNA substrates through specific adaptor proteins. Cell. 2015;162:1029-38.
293. Thyagarajan A, Strong MJ, Szaro BG. Post-transcriptional control of neurofilaments in development and disease. Exp Cell Res. 2007;313:2088-97.

294. Traiffort E, O'Regan S, Ruat M. The choline transporter-like family SLC44: properties and roles in human diseases. Mol Aspects Med. 2013;34:646-54.
295. Tricarico PM, Marcuzzi A, Piscianz E, Monasta L, Crovella S, Kleiner G. Mevalonate kinase deficiency and neuroinflammation: balance between apoptosis and pyroptosis. Int J Mol Sci. 2013;14:23274-8.
296. Tsao N, Lee MH, Zhang W, Cheng YC, Chang ZF. The contribution of CMP kinase to the efficiency of DNA repair. Cell Cycle. 2015;14:354-403.
297. Tsutsui T, Kukasawa R, Shinmyouzu K, Nakagawa R, Tobe K, Tanaka A, Ohkuma Y. Mediator complex recruits epigenetic regulators via its two cyclin-dependent kinase subunits to repress transcription of immune response genes. J Biol Chem. 2013;288:20955-65.
298. Vance JE, Campenot RB, Vance DE. The synthesis and transport of lipids for axonal growth and nerve regeneration. Biochim Biophys Acta. 2000;1486:84-96.
299. Vasanthakumar A, Godley LA. 5-hydroxymethylcytosine in cancer: significance in diagnosis and therapy. Cancer Genet. 2015;208:167-77.
300. Veitia RA, Hurst LD. Accelerated molecular evolution of insect orthologues of ERG28/C14orf1: a link with ecdysteroid metabolism? J Genet. 2001;80:17-21.
301. Veldman MB, Bemberi MA, Goldman D. Tuab1a gene expression is regulated by KLF7/7 and is necessary for CNS development and regeneration in zebrafish. Mol Cell Neurosci. 2010;43:370-83.
302. Vitavska O, Wieczorek H. Putative role of an SLC45 H^+/sugar cotransporter in mammalian spermatazoa. Pfugers Arch. 2017;469:1433-42.
303. Volkov P, Olsson AH, Gillberg L, Jorgensen SW, Brons C, Eriksson KF, Groop L, Jansson PA, Nilsson E, Ronn T, Vaag A, Ling C. A genome-wide mQTL analysis in human adipose tissue identifies genetic variants associated with DNA methylation, gene expression and metabolic traits. PLoS ONE. 2016;11:e015776.
304. Wakao J, Kishida T, Fumino S, Kimura K, Yamamoto K, Kotani SI, Mizushima K, Naito Y, Yoshikawa T, Tajiri T, Mazda O. Efficient direct conversion of human fibroblasts into myogenic lineage induced by co-transduction with *MYCL* and *MYOD1*. Biochem Biophys Res Commun. 2017;488:368-73.
305. Walker JA, Gouzi JY, Long JB, Huang S, Maher RC, Xia H, Khalil K, Ray A, Van Vactor D, Bernards R, Bernards A. Genetic and functional studies implicate synaptic overgrowth and ring gland cAMP/PKA signaling defects in the *Drosophila melanogaster* neurofibromatosis-1 growth deficiency. PLoS Genet. 2013;9:e1003958.
306. Wang C, Kang X, Zhou L, Chai Z, Wu Q, Huang R, Xu H, Hu M, Sun X, Sun S, Li J, Jiao R, Zuo P, Zheng L, Yue Z, Zhou Z. Synaptotagmin-11 is a critical mediator of parkin-linked neurotoxicity and Parkinson's disease-like pathology. Nat Commun. 2018;9:81.
307. Wang C, Wei Z, Chen K, Ye F, Yu C, Bennett V, Zhang M. Structural basis of diverse membrane target recognitions by ankyrins. eLife. 2014;3:e04353.
308. Wang GX, Cho KW, Uhm M, Hu CR, Li S, Cozacov Z, Xu AE, Cheng JX, Saltiel AR, Lumenq CN, Lin JD. Otopetrin 1 protects mice from obesity-associated metabolic dysfunction through attenuating adipose tissue inflammation. Diabetes. 2014;63:1340-52.
309. Wang H, Wang L, Cao L, Zhang Q, Meng Z, Wu X, Xu K. Inhibition of autophagy potentiates the anti-metastasis effect of phenethyl isothiocyanate through JAK2/STAT3 pathway in lung cancer cells. Mol Carcinog. 2018;57:522-35.
310. Wang H, Xu M, Kong Q, Sun P, Yan F, Tian W, Wang X. Research and progress on ClC-2 (Review). Mol Med Rep. 2017;16:11-22.
311. Wang J, Maldonado MA. The ubiquitin-proteasome system and its role in inflammatory and autoimmune diseases. Cell Mol Immunol. 2006;3:255-61.
312. Wang X, Carre W, Saxton AM, Cogburn LA. Manipulation of thyroid status and/or GH injection alters hepatic gene expression in the juvenile chicken. Cytogenet Genome Res. 2007;117:174-88.
313. Wang Z, Long H, Chang C, Zhao M, Lu Q. Crosstalk between metabolism and epigenetic modifications in autoimmune diseases: a comprehensive review. Cell Mol Life Sci. 2018;75(18):3353-69.

314. Wang Z, Reynolds A, Kirry A, Nienhaus C, Blackmore MG. Overexpression of Sox11 promotes corticospinal tract regeneration after spinal injury while interfering with functional recovery. J Neurosci. 2015;35:3139-45.
315. Wei S, Shang H, Cao Y, Wang Q. The coiled-coil domain containing protein Ccdc136b antagonizes maternal Wnt/β-catenin activity during zebrafish dorsoventral axial patterning. J Genet Genomics. 2016;43:431-8.
316. Wen H, Cao J, Yu X, Sun B, Ding T, Li M, Li D, Wu H, Long L, Xu G, Zhang F. Spatiotemporal patterns of Gem expression after rat spinal cord injury. Brain Res. 2013;1516:11-9.
317. Werner SR, Mescher AL, Neff AW, King MW, Chaturvedi S, Duffin KL, Harty MW, Smith RC. Neural MMP-28 expression precedes myelination during development and peripheral nerve repair. Dev Dyn. 2007;236:2852-64.
318. Wikberg ML, Ling A, Li X, Oberg A, Edin S, Palmqvist R. Neutrophil infiltration is a favorable prognostic factor in early stages of colon cancer. Hum Pathol. 2017;68:193-202.
319. Willer CJ, Speliotes EK, Loos RJ, Li S, Lindgren CM, Heid IM, Berndt SI, Elliott AL, Jackson AU, Lamina C, Lettre G, et al. Six new loci associated with body mass index highlight a neuronal influence on body weight regulation. Nat Genet. 2009;41:25-34.
320. Wong P, Coulombe PA. Loss of keratin 6 (K6) proteins reveals a function for intermediate filaments during wound repair. J Cell Biol. 2003;163:327-37.
321. Wu N, Yu H. The Smc complexes in DNA damage response. Cell Biosci. 2012;2:5.
322. Xiong XQ, Geng Z, Zhou B, Zhang F, Han Y, Zhou YB, Wang JJ, Gao XY, Chen Q, Li YH, Kang YM, Zhu GQ. FNDC5 attenutates adipose tissue inflammation and insulin resistance via AMPK-mediated macrophage polarization in obesity. Metabolism. 2018;83:31-41.
323. Xu C, Li Z, He H, Wernimont A, Li Y, Loppnau P, Min J. Crystal structure of human nuclear pore complex component NUP43. FEBS Lett. 2015;589:3247-53.
324. Xu C, Zheng P, Shen S, Xu Y, Wei L, Gao H, Wang S, Zhu C, Tang Y, Wu J, Zhang Q, Shi Y. NMR structure and regulated expression in APL cell of human SH3BGRL3. FEBS Lett. 2005;579:2788-94.
325. Xu P, Roes J, Segal AW, Radulovic M. The role of grancalcin in adhesion of neutrophils. Cell Immunol. 2006;240:116-21.
326. Yang X, Lu B, Sun X, Han C, Fu C, Xu K, Wang M, Li D, Chen Z, Opal P, Wen Q, Crispino JD, Wang QF, Huang Z. ANP32A regulates histone H3 acetylation and promotes leukemogenesis. Leukemia. 2018;32:1587-97.
327. Yap TE, Donna P, Almonte MT, Cordeiro MF. Real-time imaging of retinal ganglion cell apoptosis. Cells. 2018;7:E60.
328. Ye M, Li J, Gong J. PCDH10 gene inhibits cell proliferation and induces cell apoptosis by inhibiting the PI3K/Akt signaling pathway in hepatocellular carcinoma cells. Oncol Rep. 2017;37:3167-74.
329. Yeo L, Lom H, Juarez M, Snow M, Buckley CD, Filer A, Raza K, Scheel-Toellner D. Expression of FcRL4 defines a pro-inflammatory, RANKL-producing B cell subset in rheumatoid arthritis. Ann Rheum Dis. 2015;74:928-35.
330. Yin Y, Cui G, Gilbert HYY, Yang Y, Yang Z, Berlinicke C, Li Z, Zaverucha-do-Valle C, He H, Petkova V, Zack DJ, Benowitz LI. Oncomodulin links inflammation to optic nerve regeneration. Proc Natl Acad Sci USA. 2009;106:19587-92.
331. Yin Y, Henzl MT, Lorber B, Nakazawa T, Thomas TT, Jiang F, Langer R, Benowitz LI. Oncomodulin is a macrophage-derived signal for axon regeneration in retinal ganglion cells. Nature Neurosci. 2006;9:843-52.
332. Yokoyama Y, Zhu H, Zhang R, Noma K. A novel role for the condensin II complex in cellular senescence. Cell Cycle. 2015;14:2160-70.
333. Yonezawa T, Ohtsuka A, Yoshitaka T, Hirano S, Nomoto H, Yamamoto K, Ninomiya Y. Limitrin, a novel immunoglobulin superfamily protein localized to glia limitans formed by astrocyte endfeet. Glia. 2003;44:190-204.
334. Yoo BH, Park CH, Kim HJ, Kang DS, Bae CD. CKAP2 is necessary to ensure the faithful spinde bipolarity in a dividing diploid hepatocyte. Biochem Biophys Res Commun. 2016;473:886-93.

335. Yoo M, Choi KY, Kim J, Kim M, Shim J, Choi JH, Cho HY, Oh JP, Kim HS, Kaang BK, Han JH. BAF53b, a neuron-specific nucleosome remodeling factor, is induced after learning and facilitates long-term memory consolidation. J Neurosci. 2017;37:3686-97.
336. Yoshida M, Zhao L, Grigoryan G, Shim H, He P, Yun CC. Deletion of Na+/H+ exchanger regulatory factor 2 represses colon cancer progress by suppression of Stat3 and CD24. Am J Physiol Gastrointest Liver Physiol. 2016;10:G576-G598.
337. Yu Y, Chi B, Xia W, Gangopadhyay J, Yamazaki T, Winkelbauer-Hurt ME, Yin S, Eliasse Y, Adams E, Shaw CE, Reed R. U1 snRNP is mislocalized in ALS patient fibroblasts bearing NLS mutations in FUS and is required for motor neuron outgrowth in zebrafish. Nucleic Acids Res. 2015;43:3208-18.
338. Yu Y, Schachner M. Syntenin-a promotes spinal cord regeneration following injury in adult zebrafish. Eur J Neurosci. 2013;38:2280-9.
339. Yueh MF, Chen S, Nguyen N, Tukey RH. Developmental onset of bilirubin-induced neurotoxicity involves Toll-like receptor 2-dependent signaling in humanized UDP-glucuronosyltransferase1 mice. J Biol Chem. 2014;289:4699-709.
340. Zhang Y, Ng HH, Erdjument-Bromage H, Tempst P, Bird A, Reinberg D. Analysis of the NuRD subunits reveals a histone deacetylase core complex and a connection with DNA methylation. Genes Dev. 1999;13:1924-35.
341. Zhang Y, O'Leary MN, Peris S, Wang M, Zha J, Melov S, Kappes DJ, Feng Q, Rhodes J, Amieux PS, Morris DR, Kennedy BK, West DL. Ribosomal proteins Rpl22 and Rpl22l1 control morphogenesis by regulating pre-mRNA splicing. Cell Rep. 2017;18:545-56.
342. Zhao D, Zhang T, Hou XM, Ling XL. Knockdown of fascin-1 expression suppresses cell migration and invasion of non-small cell lung cancer by regulating the MAPK pathway. Biochem Biophys Res Commun. 2018;497:694-9.
343. Zhao Q, Che X, Zhang H, Tan G, Liu L, Jiang D, Zhao J, Xiang X, Sun X, He Z. Thioredoxin-interacting protein mediates apoptosis in early brain injury after subarachnoid haemorrhage. Int J Mol Sci. 2017;18:pii: E854.
344. Zhao S, Yin J, Zhou L, Yan F, He Q, Huang L, Peng s, Jia J, Cheng J, Chen H, Tao W, Ji X, Xu Y, Yuan Z. Hippo/MST1 signaling mediates microglial activation following acute cerebral ischemia-reperfusion injury. Brain Behav Evol. 2016;55:236-48.
345. Zhou C, Wang Y, Peng J, Li C, Liu P, Shen X. SNX10 plays a critical role in MMP9 secretion via JNK-p38-ERK signaling pathway. J Cell Biochem. 2017;118:4664-71.
346. Zhu D, Kosik KS, Meigs TE, Yanamadala V, Denker BM. Gα_{12} directly interacts with PP2A: evidence for Gα_{12}-stimulated PP2A phosphatase activity and dephosphorylation of microtubule-associated protein, tau. J Biol Chem. 2004;279:54983-6.
347. Zhu X, Sun Y, Mu X, Guo P, Gao F, Zhang J, Zhu Y, Zhang X, Chen L, Ning Z, Bai Y, Ren J, Man M, Liu P, Hu L. Phospholipase Cε deficiency delays the early stage of cutaneous wound healing and attenuates scar formation in mice. Biochem Biophys Res Commun. 2017;484:144-51.
348. Zhu X, Xie C, Li YM, Huang ZL, Zhao QY, Hu ZX, Wang PP, Gu YR, Gao ZL, Peng L. TMEM2 inhibits hepatitis B virus infection in HepG2 and HepG2.2.15 cells by activating the JAK-STAT signaling pathway. Cell Death Dis. 2016;7:e2239.
349. Zuiderweg ER, Hightower LE, Gestwicki JE. The remarkable multivalency of the Hsp70 chaperones. Cell Stress Chaperones. 2017;22:173-89.
